# Muraymycin Nucleoside Antibiotics: Structure-Activity Relationship for Variations in the Nucleoside Unit

**DOI:** 10.3390/molecules25010022

**Published:** 2019-12-19

**Authors:** Anna Heib, Giuliana Niro, Stefanie C. Weck, Stefan Koppermann, Christian Ducho

**Affiliations:** Department of Pharmacy, Pharmaceutical and Medicinal Chemistry, Saarland University, Campus C2 3, 66123 Saarbrücken, Germany; s9aahei2@stud.uni-saarland.de (A.H.); giuliana.niro@uni-saarland.de (G.N.); stefanie.weck@uni-saarland.de (S.C.W.); stefan.koppermann@uni-saarland.de (S.K.)

**Keywords:** antibiotics, natural products, nucleoside analogues, structure–activity relationships

## Abstract

Muraymycins are a subclass of naturally occurring nucleoside antibiotics with promising antibacterial activity. They inhibit the bacterial enzyme translocase I (MraY), a clinically yet unexploited target mediating an essential intracellular step of bacterial peptidoglycan biosynthesis. Several structurally simplified muraymycin analogues have already been synthesized for structure–activity relationship (SAR) studies. We now report on novel derivatives with unprecedented variations in the nucleoside unit. For the synthesis of these new muraymycin analogues, we employed a bipartite approach facilitating the introduction of different nucleosyl amino acid motifs. This also included thymidine- and 5-fluorouridine-derived nucleoside core structures. Using an in vitro assay for MraY activity, it was found that the introduction of substituents in the 5-position of the pyrimidine nucleobase led to a significant loss of inhibitory activity towards MraY. The loss of nucleobase aromaticity (by reduction of the uracil C5-C6 double bond) resulted in a ca. tenfold decrease in inhibitory potency. In contrast, removal of the 2′-hydroxy group furnished retained activity, thus demonstrating that modifications of the ribose moiety might be well-tolerated. Overall, these new SAR insights will guide the future design of novel muraymycin analogues for their potential development towards antibacterial drug candidates.

## 1. Introduction 

**Scheme 1:**
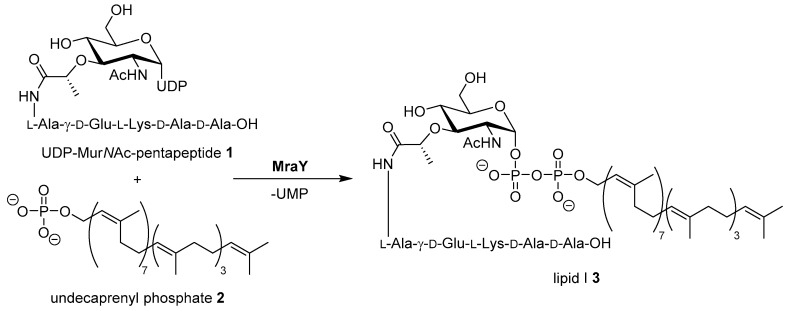
Reaction of Park’s nucleotide **1** with undecaprenyl phosphate **2** yielding lipid I **3**, catalyzed by MraY (translocase I). UDP = uridine diphosphate, UMP = uridine monophosphate. The exact composition of the pentapeptide moiety varies among different bacteria [12].

The increasing number of bacterial infections with strains that are resistant against established antibiotics are a major challenge in current and future clinical healthcare [1,2]. For the development of new antimicrobial drug candidates, cross resistances with established antibiotics are a central concern. Therefore, novel targets and yet unexploited modes of action need to be identified [3]. A promising target not yet addressed by clinically established classes of antibiotics is the bacterial enzyme MraY (translocase I) which mediates a key step in the intracellular stages of bacterial cell wall biosynthesis [4,5,6]. MraY catalyzes the first membrane-associated step in the cytosolic stage of peptidoglycan synthesis, i.e., the formation of the membrane-bound intermediate lipid I by reaction of UDP-Mur*N*Ac pentapeptide (’Park’s nucleotide’) with the isoprenoid membrane anchor undecaprenyl phosphate (Scheme 1) [7,8,9,10,11,12,13].

**Figure 1:**
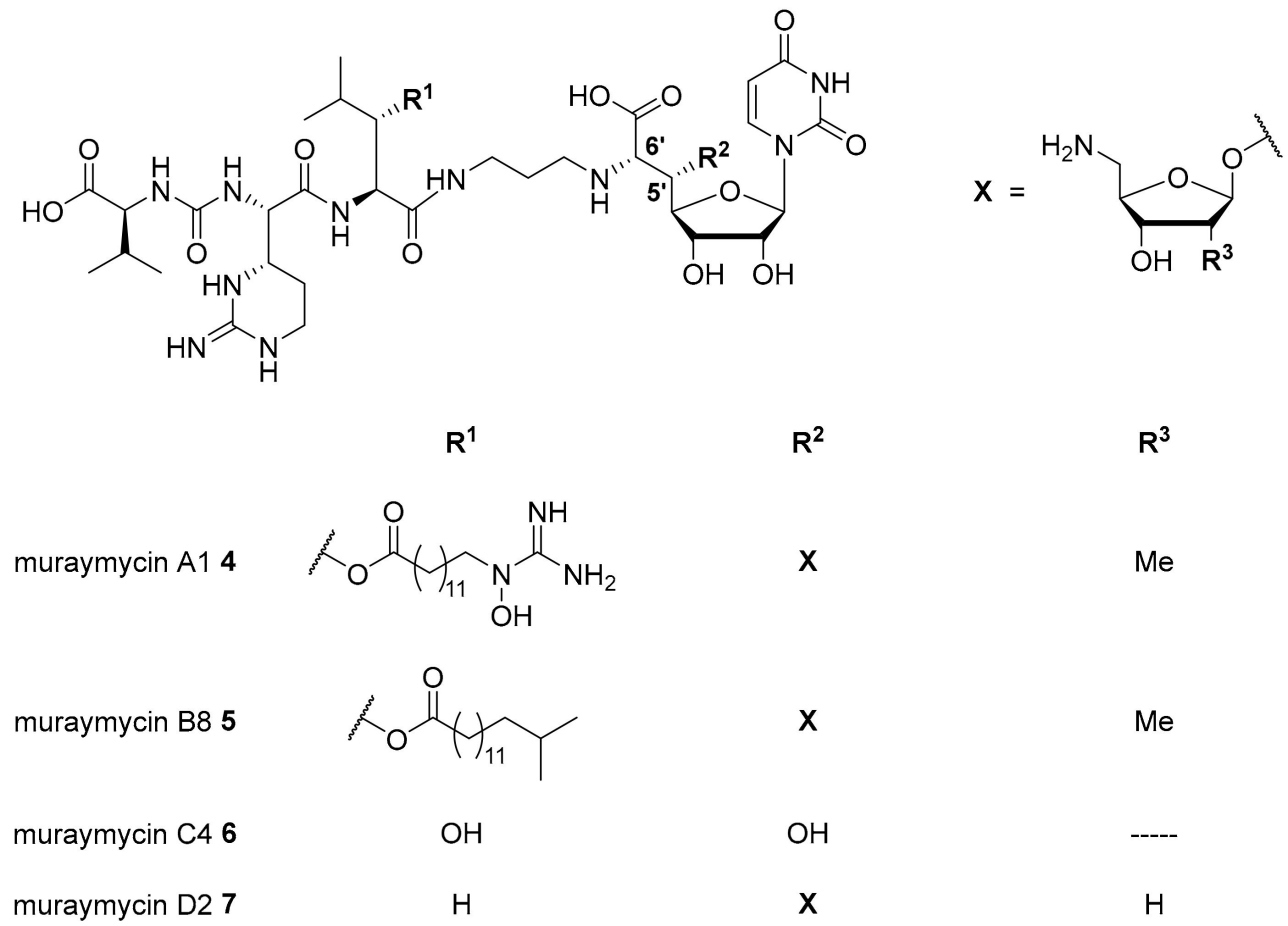
Structures of selected naturally occurring muraymycins **4**–**7** [18,19,20].

Nucleoside antibiotics are naturally produced secondary metabolites acting as MraY inhibitors. Several subclasses such as muraymycins, mureidomycins, tunicamycins, liposidomycins, and capuramycins have been reported [13,14,15,16]. Our research on nucleoside antibiotics mainly focusses on muraymycins which were isolated from *Streptomyces* sp. as a set of 19 structurally varying natural products [13,17]. Recently, several new members of this subclass have been reported [18]. Muraymycins can be divided into four groups (A–D, Figure 1). They share a joint backbone structure, i.e., a uridine-derived nucleosyl amino acid (’glycyluridine’, GlyU) motif and a urea-containing peptide moiety, but vary in the substitution pattern at the central L-leucine unit and in the presence of aminoribosyl substituents in the 5′-position.

In the A- and B-series (e.g., muraymycins A1 **4** and B8 **5**, Figure 1), lipophilic side chains are present which are connected to the backbone as fatty acid esters of β-hydroxylated L-leucine. Such motifs are not found in the C- and D-series congeners (e.g., muraymycins C4 **6** and D2 **7**, Figure 1). Different aminoribosyl substituents (**X** in Figure 1) at the GlyU core structure are present in most naturally occurring muraymycins. Structures containing hydroxy groups in the 5′-position (e.g., **6**) are discussed to be hydrolysis products formed in the isolation and purification process of the natural products [19,20,21].

**Figure 2:**
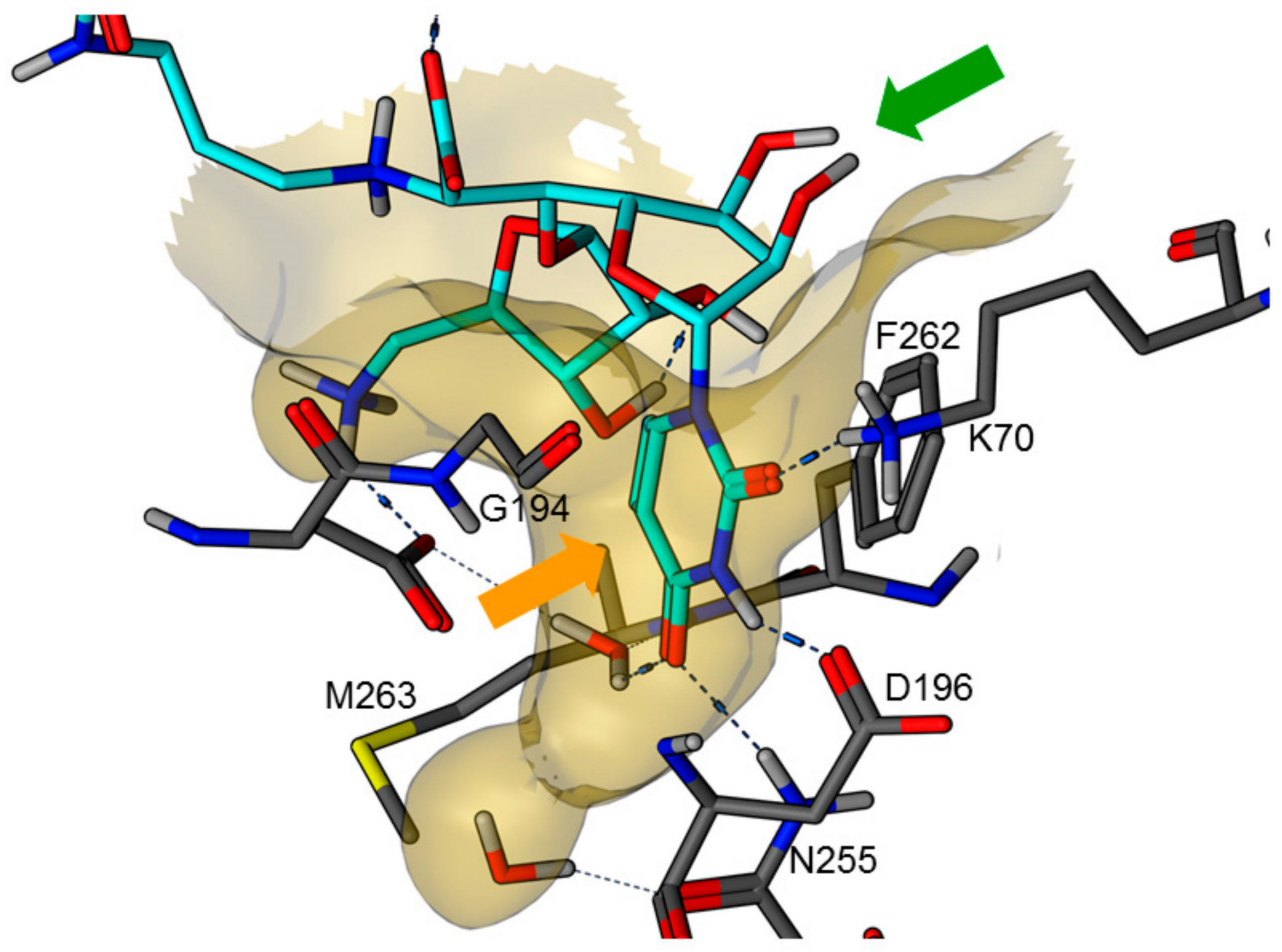
X-ray co-crystal structure of MraY from *Aquifex aeolicus* in complex with muraymycin D2 **7** (PDB 5CKR) [22,23]: protein-inhibitor interactions at the GlyU binding site (hydrocarbon scaffold of inhibitor **7** in light blue; orange arrow: well-defined uracil binding pocket; green arrow: ribose moiety of the uridine-derived GlyU unit showing no significant interactions of 2′-OH and 3′-OH).

In 2016, an X-ray co-crystal structure of the natural product muraymycin D2 **7** in complex with MraY from *Aquifex aeolicus* was reported by Chung et al. [22,23]. These structural insights into the target interaction of muraymycins allowed an identification of possible key interactions which were used to rationalize the design of synthetic muraymycin analogues for SAR studies [24]. A comparison of the aforementioned co-crystal structure with the previously reported crystal structure of the ligand-free *apo* enzyme [25] revealed a large conformational change due to inhibitor binding, hinting to a pronounced conformational plasticity of MraY. In this context, the formation of a well-defined binding pocket for the uridine-derived GlyU moiety was observed (Figure 2). This was regarded as one of the main reasons for the high binding affinity of naturally occurring nucleoside antibiotics despite their structural deviation from the natural substrate, i.e., UDP-Mur*N*Ac pentapeptide **1** [22,23,25]. Very recently, a more comprehensive model for MraY binding of diverse classes of nucleoside antibiotics, again based on X-ray crystallographic studies, has been reported [26].

**Figure 3:**
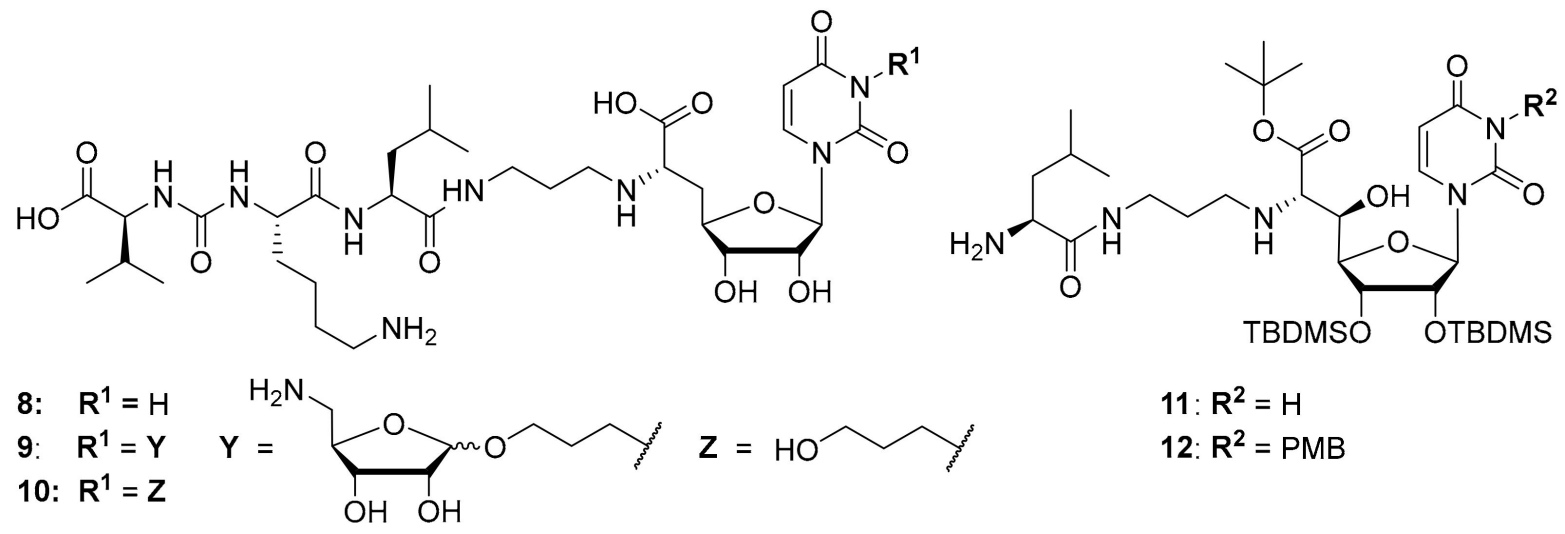
Structures of previously reported 5′-deoxy muraymycin analogues **8**–**10** [24] and the previously reported protecting group-containing analogues **11** and **12** (PMB = *p*-methoxybenzyl, TBDMS = *tert*-butyldimethylsilyl) [34].

Synthetic access to the naturally occurring muraymycins is challenging due to structural features such as the non-proteinogenic amino acid L-epicapreomycidine, the aminoribosylated GlyU moiety and the (3*S*)-3-hydroxy-L-leucine unit. Total syntheses of some naturally occurring muraymycins have been reported though [13,27,28,29], and synthetic routes towards crucial structural motifs have been developed [30,31,32,33]. Both for potential applications and for more straightforward SAR studies, several simplified muraymycin analogues have been designed [34,35,36,37,38]. By comparison of different natural products and synthetic analogues with long alkyl side chains, it was shown that increased lipophilicity improves antibacterial activities (MIC values), but has only a minor effect on MraY inhibition [17,20,39,40]. This is probably owed to an improved cellular uptake of lipophilically decorated muraymycins. Through design of simplified analogues, the terminal urea dipeptide motif [40] as well as the 5′-*O*-aminoribosyl substituent [21,39] were identified to mediate key interactions in MraY inhibition. However, synthetically obtained 5′-defunctionalized (′5′-deoxy’) muraymycin C4 that was reported by our group [38] was found to still inhibit MraY in the nM range, even though most naturally occurring muramycins are MraY inhibitors with pM activities [39]. The replacement of (3*S*)-3-hydroxy-L-leucine with L-leucine resulted in a ~25-fold loss of activity [41], but that implied that such simplified analogues may still be employed in SAR studies. The exact structure of the amino acid side chains in the urea dipeptide moiety seems to only have limited impact on MraY inhibition [21]. These findings led to the design of simplified analogues containing L-lysine instead of L-epicapreomycidine (e.g., analogue **8**, Figure 3, MraY inhibition: IC_50_ = 2.5 μM), thus further reducing synthetic effort [24]. Regarding the stereochemistry of the nucleoside moiety, the naturally occurring (6′*S*)-configuration was found to be preferred. If a 5′-hydroxy group was present, at least either C-5′ or C-6′ needed to have their naturally occurring configuration in order to retain good inhibitory activity towards MraY in vitro [41].

It was also attempted to synthesize and study analogues bearing the aminoribosyl substituent (**X** in Figure 1) at different positions of the muraymycin scaffold. This led to compounds **9** and **10** (with **10** serving as a reference, Figure 3) which were modified at the N-3 position of the uracil base. However, these analogues showed no inhibitory activity against MraY [24]. Remarkably, previously reported simplified analogues **11** and **12** (which still contained synthetic TBDMS and, in case of **12**, PMB protecting groups, Figure 3) had been described to be antibacterially active MraY inhibitors, in spite of the modification of the uracil base in compound **12** [34].

**Scheme 2:**
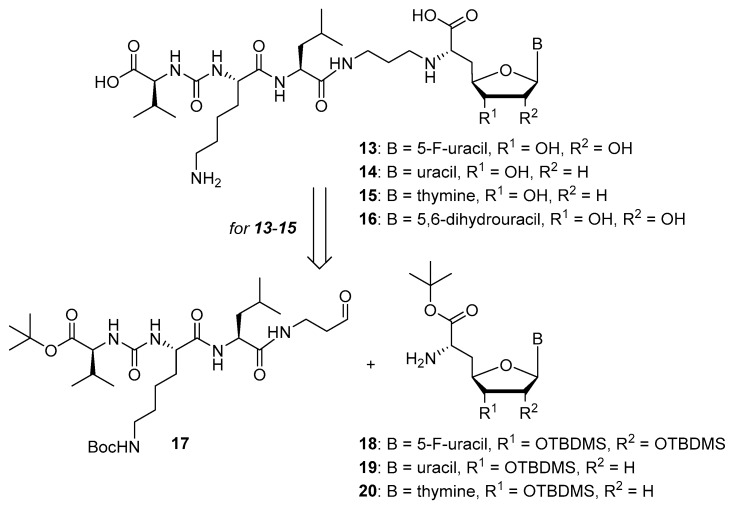
Target structures **13–16** of this study and retrosynthetic strategy for **13–15**.

Overall, the X-ray co-crystal structure (Figure 2) and the results with analogues **9** and **10** (Figure 3) suggest that some structural variations in the muraymycin nucleoside moiety might significantly hamper MraY inhibition. This is particularly the case for modifications of the uracil base with respect to the well-defined uracil binding pocket of MraY (Figure 2, orange arrow). In contrast, binding of the GlyU ribose moiety appears to be far less specific, with the X-ray co-crystal structure showing no significant interactions of the 2′-OH and 3′-OH groups (Figure 2, green arrow). However, the pronounced conformational plasticity of MraY (vide supra) might generally enable its adaptation to structural variations of inhibitors, which would be very hard to predict from the (inherently static) X-ray co-crystal structure. In this work, we have therefore decided to experimentally probe the SAR of the muraymycin nucleoside moiety by introducing structural changes to it which were supposed to be more subtle than the ones in analogues **9**–**12** (cf. Figure 3). These considerations have led to the design of the following target compounds (Scheme 2), which were based on the previously described 5′-defunctionalized muraymycin analogue **8** (vide supra): (i) a derivative **13** containing 5-fluorouracil as a nucleobase; (ii) 2′-deoxy analogue **14**; (iii) thymidine-derived analogue **15**. This would enable us to systematically study both the influence of different C-5 substituents of the pyrimidine base (including the potential electronic effect of fluorine) and the significance of the 2′-functionalization of the ribose moiety. Furthermore, we have also envisioned to investigate a fourth target structure to evaluate the relevance of nucleobase aromaticity, i.e., derivative **16** having non-aromatic 5,6-dihydrouracil as a nucleobase (Scheme 2). Overall, target structures **13**–**16** were chosen based on the rationale that multiple SAR insights (regarding C-5-substitution, ribose modification, and nucleobase aromaticity) should be obtained with reasonable synthetic effort.

## 2. Results

### 2.1. Synthesis of Muraymycin Analogues

We had previously described different solution-phase syntheses of muraymycin analogues, either based on a tripartite [38] or a bipartite [41] strategy. However, we have recently reported a novel synthetic approach towards muraymycin analogues, based on solid-phase peptide synthesis (SPPS) to construct a building block for the complete peptide unit [42]. We have decided to apply this new strategy for the synthesis of target structures **13**–**15** (Scheme 2). Thus, these muraymycin analogues should be accessible by reductive amination of the peptide-derived aldehyde **17** with different nucleosyl amino acids **18**–**20**, to be followed by global acidic deprotection. The protected peptide building block **17** was readily accessible using our novel SPPS method as described [42], leaving the main synthetic challenge to be the stereoselective preparation of nucleosyl amino acids **18**–**20**. For the synthesis of 5,6-dihydrouracil derivative **16**, we have used a solution-phase route (vide infra) as this compound had already been prepared prior to the recent development of the SPPS-based method.

**Scheme 3:**
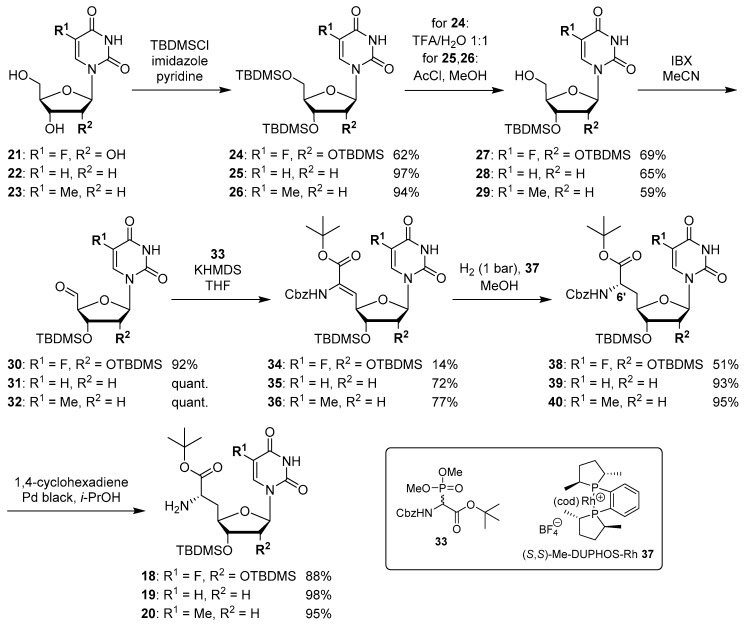
Stereocontrolled synthesis of (6′*S*)-configured nucleosyl amino acids **18**–**20**.

The synthesis of nucleosyl amino acids **18**–**20** was performed in analogy to the previously reported stereocontrolled synthesis of other uridine-derived 5’-deoxy nucleosyl amino acids (Scheme 3) [31]. Commercially available nucleosides 5-fluorouridine **21**, 2’-deoxyuridine **22** and thymidine **23** were fully TBDMS-protected to give persilylated nucleosides **24**–**26** (yields 62%–97%). The protocols for subsequent selective 5’-*O*-desilylation had to be modified with respect to the different nucleoside structures. Thus, for 5-fluorouridine derivative **24**, deprotection towards product **27** with aqueous TFA [43] proceeded with good yield (69%) and regioselectivity. For **25** and **26**, however, reduced steric differentiation of the 3’- and 5’-positions (due to the absence of a 2′-*O*-TBDMS moiety adjacent to the 3′-position) led to poor selectivity under similar conditions. Therefore, an alternative method using milder acidic conditions [44] was applied: Hydrochloric acid was generated in situ by reaction of acetyl chloride with methanol at lower temperatures. This protocol furnished 5’-*O*-desilylated products **28** and **29** in yields of 65% and 59%, respectively. Nucleosides **27**–**29** were then oxidized to the corresponding 5′-aldehydes **30**–**32** in 92% to quantitative yields. Subsequent Wittig–Horner reaction of these aldehydes with phosphonate **33** [31] gave *Z*-configured didehydro nucleosyl amino acids **34**–**36**. For **35** and **36**, this transformation proceeded with good yields of 72% and 77%, respectively. In the case of the 5-fluorouridine derivative **34,** a low yield of 14% was obtained (Scheme 3). This was attributed to the increased acidity of the 5-fluorouracil imide 3-NH, caused by the electron-withdrawing fluorine substituent. Hence, the imide is supposed to (at least partially) protonate the phosphonate anion, thus resulting in incomplete conversion and potentially also partial decomposition of unconverted aldehyde **30**. Using an excess of KHMDS to avoid this side reaction resulted in a loss of stereoselectivity, probably due to base-mediated isomerization of the C-5′-C6′ double bond (reactions not shown). We had studied before if 3-*N*-protection is a viable option for this synthetic route, but results were disappointing mainly due to difficult deprotection reactions [31]. Hence, no further optimization of the synthesis of **34** was attempted and it was decided instead to proceed with the obtained material. For all didehydro nucleosyl amino acids **34**–**36**, the assignment of the *Z*-stereochemistry of the C-5′-C6′ double bond was based on precedent [31] and ^1^H NMR spectroscopic data (i.e., characteristic chemical shifts of the 5′-H [31]).

The next step was the asymmetric hydrogenation of **34**–**36** using the chiral (*S*,*S*)-Me-DUPHOS-Rh(I) catalyst **37** [31,45] (Scheme 3). It had been proven before that this catalyst selectively furnishes L-amino acids from *Z*-didehydro amino acid precursors [31,46,47], thus giving rise to the formation of *N*-Cbz-protected (6′*S*)-configured nucleosyl amino acids **38**–**40** in yields of 51%–95%. The formation of the unwanted (6′*R*)-epimer was not observed in any of these reactions. Finally, Cbz deprotection under transfer hydrogenation conditions (to avoid reduction of the pyrimidine C-5-C-6 double bond [31]) afforded (6′*S*)-nucleosyl amino acids **18**–**20** in yields of 88%–98% (Scheme 3).

**Scheme 4:**
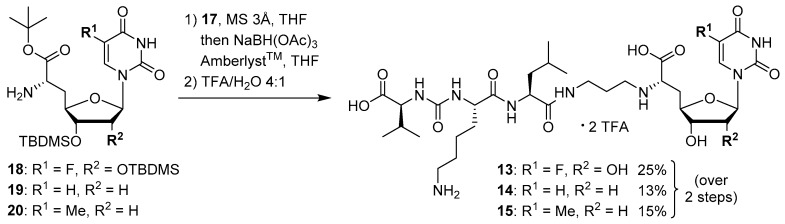
Synthesis of target structures **13–15**.

The synthesis of the peptide-aldehyde unit **17**, based on a solid phase-supported approach, has been performed as recently reported [42]. Thus, the stage was set for the preparation of target structures **13**–**15** (Scheme 4). Nucleosyl amino acids **18**–**20** were connected with peptide-aldehyde **17** by reductive amination. This transformation had been described before as a key step in the synthesis of muraymycin analogues [24,31,38]. Subsequent global acidic deprotection using 80% aqueous TFA and HPLC purification afforded target compounds **13**–**15** as their bis-TFA salts in 13%–25% yields over two steps from **18**–**20** (Scheme 4).

**Scheme 5:**
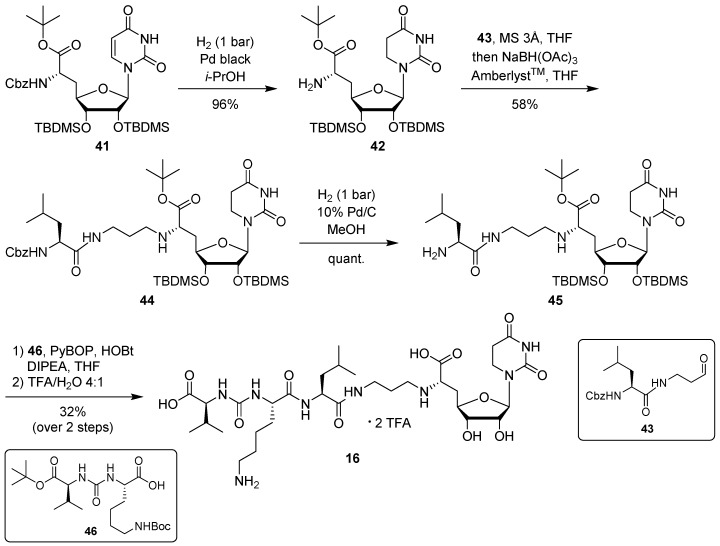
Synthesis of target structure **16**.

For the synthesis of target compound **16** containing 5,6-dihydrouracil as a nucleobase, our previously developed solution-phase tripartite approach was applied [13,38]. The nucleosyl amino acid building block was prepared by hydrogenation of **41** [31] to give concomitant Cbz cleavage and reduction of the uracil-C5-C6 double bond, affording **42** in 96% yield (Scheme 5). This reduction of the uracil nucleobase can otherwise be observed as a side reaction during the hydrogenolytic deprotection of nucleosyl amino acids [31].

Introduction of the propyl linker and the leucine moiety was achieved by reductive amination of **42** with aldehyde **43** [24,34], furnishing **44** in 58% yield (Scheme 5). After hydrogenolytic Cbz cleavage (product **45**, quantitative yield), urea dipeptide building block **46** [24,41] was employed in a peptide coupling step to construct the full-length muraymycin scaffold. This was followed by global acidic deprotection and HPLC purification to give target compound **16** (as its bis-TFA salt) in 32% yield over two steps from **45** (Scheme 5).

### 2.2. Biological Evaluation

Target structures **13**–**16** were tested for their inhibitory potential towards the bacterial target enzyme MraY in vitro. For these studies, we used an established fluorescence-based assay for MraY activity, which employs a dansylated version of Park’s nucleotide **1** and MraY from *S. aureus* (heterologously overexpressed in *E. coli*) [18,39,48,49,50,51,52]. The obtained inhibitory activities (IC_50_ values, also see Supplementary Materials Figures S1–S4, for measured data and inhibition curves) are listed in Table 1, including the potency of previously reported reference compound **8** [24,41]. As target structures **13** and **15** turned out to be nearly inactive as MraY inhibitors in the relevant concentration range (≤100 μM), we also tested them at a significantly higher concentration (1 mM, Table 1). Muraymycin analogues **13**–**16** were also investigated for their antibacterial activities in cellulo against *E. coli*, but were inactive (MIC > 50 μg/mL).

## 3. Discussion

The synthesis of the target structures **13**–**16** was successfully accomplished. For **13**–**15**, we have applied a recently reported novel approach which is based on the solid phase-supported synthesis of the peptide–aldehyde unit **17**, which was then connected with a nucleosyl amino acid building block **18**–**20** (Scheme 2) [42]. Thus, we mainly had to focus on the preparation of **18**–**20**, which was performed using an established route with Wittig–Horner reaction and subsequent asymmetric hydrogenation as key steps for the stereoselective construction of the amino acid motif [31]. It was thus demonstrated that this protocol is applicable for the introduction of different nucleoside core structures. In contrast to this bipartite strategy, target compound **16** was prepared using our previously employed solution-phase tripartite approach [13,38]. This enabled a direct comparison of both routes. With respect to the late-stage introduction of the nucleosyl amino acid moiety, the solid phase-supported bipartite synthesis (Scheme 3 and Scheme 4) appears to be superior and more efficient for studies on analogues with varying nucleoside structures.

The in vitro assay for inhibitory activities of **13**–**16** towards the bacterial target protein MraY provided highly interesting, unprecedented SAR insights. Compound **13** differs from reference compound **8** only in the formal substitution of the uracil-5-H with a fluorine atom. Nevertheless, this minor structural alteration leads to a nearly complete loss of inhibitory activity (Table 1). In contrast, the comparison of 2′-deoxyuridine-derived analogue **14** with **8** reveals a full retention of inhibitory activity (within the error margin of the assay). This suggests that structural variations in the ribose moiety appear to be much better tolerated than nucleobase modifications. Accordingly, when an additional 5-methyl substituent was formally introduced into the structure of **14** (i.e., thymidine analogue **15**), the inhibitory activity was nearly completely lost again. The only partially tolerated modification of the pyrimidine base studied in this work was the reduction of the C5-C6 double bond, as **16** was only ca. tenfold less active than **8**.

These results are in good agreement with the insights obtained from the X-ray crystal structure of MraY from *Aquifex aeolicus* in complex with muraymycin D2 **7** [22,23] (vide supra, Figure 2). The well-defined binding pocket for the uracil base indeed seems to preclude the introduction of substituents into this moiety. In the case of 5-fluoro substitution, it is not fully clear though if the loss of inhibitory activity is a result of steric hindrance or electronic effects (with respect to the strong electron-withdrawing nature of fluorine). Key interactions in the uracil binding pocket include hydrogen bonding with Lys70, Asp196, and Asn255 (Figure 2). The formation and/or strength of these hydrogen bonds might be hampered by the electron deficiency of the 5-fluorouracil moiety. The non-aromatic nucleobase in analogue **16** is probably not sterically hindered, but cannot undergo the same π–π interaction with Phe262 as uracil, thus resulting in a moderate loss of activity. However, the retention of inhibitory activity for 2′-deoxy congener **14** could also be predicted from the X-ray crystal structure as it had indicated no obvious interaction of the 2′-hydroxy group (vide supra, Figure 2). Overall, our findings demonstrate that X-ray crystal structures of MraY-inhibitor complexes appear to be excellent starting points for the structure-based design of new inhibitors, at the very least with respect to variations in the uridine-derived core unit.

The lack of antibacterial activities of target structures **13**–**16** in cellulo was expected. With respect to their potential to inhibit MraY, analogues **14** and **16** were the only candidates for showing antibacterial activity anyway. As part of our previous work on muraymycins, we had shown that there might be a ’threshold’ of inhibitory activity towards MraY for observing antibacterial potency in cellulo, concluding that a low-nM (or stronger) inhibitory potency against MraY might be a prerequisite for sufficient antibacterial activity [24,41]. As **14** and **16** were not within this activity range as MraY inhibitors, their non-activity in cellulo was in good agreement with this hypothesis. It should be pointed out that the main goal of this work was to obtain new SAR insights on MraY inhibition and not to optimize muraymycin analogues for improved antibacterial potencies. The latter can probably be achieved at a later stage by making such muraymycin analogues more lipophilic, as their polar scaffold surely impairs cellular uptake.

## 4. Materials and Methods

### 4.1. Synthesis of Muraymycin Analogues

General methods: All chemicals were purchased from standard suppliers. Reactions involving oxygen and/or moisture sensitive reagents were carried out under an atmosphere of nitrogen or argon using anhydrous solvents. Anhydrous solvents were obtained in the following manner: THF and MeCN were dried with a solvent purification system (MBRAUN MB SPS 800). Pyridine and *i*-PrOH were dried over CaH_2_ and distilled. MeOH was dried over activated molecular sieves (3 Å) and degassed. All of the thus obtained solvents were stored over molecular sieves (4 Å; in case of MeOH 3 Å). All other solvents were of technical quality and distilled prior to use, and deionized water was used throughout. Column chromatography was carried out on silica gel 60 (0.040–0.063 mm, 230–400 mesh ASTM, VWR) under flash conditions except where indicated. TLC was performed on aluminum plates precoated with silica gel 60 F_254_ (VWR). Visualization of the spots was carried out using UV light (254 nm) and/or staining under heating (H_2_SO_4_ staining solution: 4 g vanillin, 25 mL conc. H_2_SO_4_, 80 mL AcOH and 680 mL MeOH; KMnO_4_ staining solution: 1 g KMnO_4_, 6 g K_2_CO_3_ and 1.5 mL 1.25 M NaOH solution, all dissolved in 100 mL H_2_O; ninhydrin staining solution: 0.3 g ninhydrin, 3 mL AcOH and 100 mL 1-butanol; bromocresol green staining solution: 0.1 g bromocresol green, 500 mL EtOH, 5 mL 0.1 M aqueous NaOH solution). Semipreparative HPLC was performed on a VWR-Hitachi system equipped with an L-2300 pump, an L-2200 autosampler, an L-2300 column oven (24 °C), an L-2455 Diode Array Detector (DAD) and a LiChroCart^TM^ column (10 × 250 mm) containing reversed phase silica gel Nucleodur™ 100-5 C18ec (10 µm) purchased from Macherey-Nagel. Method 1: eluent A water (+0.1% TFA), eluent B MeCN (+0.1% TFA); 0–15 min gradient of B (10%–20%), 15–25 min gradient of B (20%–40%), 25–32 min gradient of B (40%–100%), 32–40 min 100% B, 40–41 min gradient of B (100%–30%), 41–45 min 30% B; flow 2 mL/min. Method 2: eluent A water (+0.1% TFA), eluent B MeCN (+0.1% TFA); 0–15 min gradient of B (20%–30%), 15–25 min gradient of B (30%–40%), 25-32 min gradient of B (40%–80%), 32–40 min 80% B, 40–41 min gradient of B (80%–30%), 41–45 min 30% B; flow 2 mL/min. Method 3: eluent A water, eluent B MeCN, 0–25 min gradient of B (5%–50%), 25–40 min gradient of B (50%–100%), 40–45 min 100% B, 45–50 min gradient of B (100%–5%), flow 5 mL/min. 500 MHz-^1^H, 126 MHz-^13^C, as well as 202 MHz-^31^P NMR spectra were recorded on a Bruker UltraShield^TM^-500 spectrometer. 376 MHz-^19^F NMR spectra were recorded on a Bruker UltraShield^TM^-400 spectrometer. 300 MHz-^1^H and 76 MHz-^13^C NMR spectra were recorded on a Bruker Fourier 300 spectrometer. All ^13^C and ^19^F NMR spectra are ^1^H-decoupled. All spectra were recorded at room temperature except where indicated and were referenced internally to solvent reference frequencies wherever possible. Chemical shifts (δ) are quoted in ppm and coupling constants (*J*) are reported in Hz. Assignment of signals was carried out using H,H-COSY, HSQC, and HMBC spectra obtained on the spectrometers mentioned above. The numbering of atoms of muraymycin target structures is depicted in the Supplementary Materials Figure S5. Mass spectra were measured on a Finnigan Surveyor MSQ Plus mass spectrometer. For ESI measurements in the positive mode, solutions in MeOH were used. High resolution mass spectra were measured on a Thermo Scientific Q Exactive Orbitrap mass spectrometer with ESI ionization mode and on a Finnigan sectorfield mass spectrometer type MAT 95S with chemical ionization (CI).

5-Fluorouridine-derived muraymycin analogue (**13**): Nucleosyl amino acid ester **18** (35 mg, 58 μmol) was dissolved in THF (3 mL), and a solution of aldehyde **17** [42] (46 mg, 75 μmol) in THF (3 mL) was added. The reaction mixture was stirred over molecular sieve (3 Å) at rt for 24 h. NaBH(OAc)_3_ (33 mg, 0.16 mmol) and Amberlyst 15^TM^ (3.4 mg, 11 μmol) were added and stirring was continued at rt for 24 h. The reaction mixture was filtered and the residue was washed with EtOAc (150 mL). The combined filtrates were washed with sat. NaHCO_3_ solution (100 mL) and brine (50 mL). The aqueous layer was extracted with EtOAc (3 × 100 mL). The combined organics were dried over Na_2_SO_4_ and the solvent was evaporated under reduced pressure. The resultant crude product was purified by column chromatography (98:2→95:5, CH_2_Cl_2_-MeOH) to give the fully protected muraymycin analogue as a colorless solid. This was dissolved in aq. TFA (80%, 4.6 mL) and stirred at rt for 24 h. The reaction mixture was then diluted with water (12 mL) and the solvent was evaporated under reduced pressure. The resultant crude product was purified by semipreparative HPLC (method 1) to give **13** (bis-TFA salt) as a colorless foam (11 mg, 25% over 2 steps from **18**). ^1^H NMR (500 MHz, D_2_O): δ [ppm] = 0.83 (d, *J* = 6.0 Hz, 3H, Leu-5-H), 0.88 (d, *J* = 5.7 Hz, 3H, Leu-5-H), 0.89 (d, *J* = 6.9 Hz, 3H, Val-4-H), 0.93 (d, *J* = 6.9 Hz, 3H, Val-4-H), 1.33–1.48 (m, 2H, Lys-4-H), 1.50–1.70 (m, 6H, Lys-3-H_a_, Lys-5-H, Leu-3-H, Leu-4-H), 1.73–1.87 (m, 1H, Lys-3-H_b_), 1.85–1.92 (m, 2H, 2″-H), 2.10–2.16 (m, 1H, Val-3-H), 2.23–2.33 (m, 1H, 5′-H_a_), 2.39–2.44 (m, 1H, 5′-H_b_), 2.96 (t, *J* = 7.6 Hz, 2H, Lys-6-H), 3.06 (t, *J* = 7.6 Hz, 2H, 1″-H), 3.20–3.29 (m, 2H, 3″-H_a_, 3″-H_b_), 3.85–3.90 (m, 1H, 6′-H), 4.03 (dd, *J* = 12.3, 6.3 Hz, 1H, 3′-H), 4.05 (d, *J* = 5.4 Hz, 1H, Val-2-H), 4.09 (dd, *J* = 8.5, 5.4 Hz, 1H, Lys-2-H), 4.14 (ddd, *J* = 9.8, 6.3, 2.8 Hz, 1H, 4′-H), 4.23 (dd, *J* = 10.1, 5.0 Hz, 1H, Leu-2-H), 4.35 (dd, *J* = 5.7, 3.8 Hz, 1H, 2′-H), 5.73 (d, *J* = 3.8 Hz, 1H, 1′-H), 7.81 (d, *J* = 6.3 Hz, 1H, 6-H). ^13^C NMR (126 MHz, D_2_O): δ [ppm] = 17.02 (Leu-C-5), 18.55 (Leu-C-5), 20.70 (Val-C-4), 22.08 (Lys-C-4), 22.15 (Val-C-4), 24.44 (Lys-C-5), 25.70 (C-2″), 26.31 (Leu-C-4), 30.02 (Val-C-3), 30.83 (Lys-C-3), 32.91 (C-5′), 36.01 (C-3′), 39.28 (Lys-C-6), 39.55 (Leu-C-3), 44.36 (C-1″), 52.65 (Leu-C-2), 54.11 (Lys-C-2), 58.95 (Val-C-2), 59.70 (C-6′), 72.83 (C-2′), 72.89 (C-3′), 79.96 (C-4′), 91.38 (C-1′), 116.45 (d, *J*_CF_ = 291.4 Hz, TFA-CF_3_), 126.45 (d, *J*_CF_ = 34.8 Hz, C-6), 140.76 (d, *J*_CF_ = 233.7 Hz, C-5), 150.02 (C-2), 159.36 (urea-C=O), 159.55 (C-4), 163.07 (d, *J*_CF_ = 35.8 Hz, TFA-COO), 172.02 (C-7′), 174.98 (Leu-C-1), 175.48 (Lys-C-1), 176.80 (Val-C-1). ^19^F NMR (376 MHz, D_2_O): δ [ppm] = −166.24 (1F, 5-F), −75.59 (6F, TFA-CF_3_). MS (ESI^+^): *m/z* = 761.5 [M + H]^+^. HRMS (ESI^+^): calcd.: 761.3840 [M + H]^+^, 783.3659 [M + Na]^+^, found: 761.3843, 783.3646. HPLC (semipreparative): t_R_ = 24.2 min (method 1).

2’-Deoxyuridine-derived muraymycin analogue (**14**): Nucleosyl amino acid ester **19** (12.6 mg, 27.6 μmol) was dissolved in THF (1.7 mL), and a solution of aldehyde **17** [42] (17 mg, 28 μmol) in THF (1.7 mL) was added. The reaction mixture was stirred over molecular sieve (3 Å) at rt for 24 h. NaBH(OAc)_3_ (12.3 mg, 58.0 μmol) and Amberlyst 15^TM^ (1.9 mg, 5.5 μmol) were added and stirring was continued at rt for 24 h. The reaction mixture was filtered and the residue was washed with EtOAc (150 mL). The combined filtrates were washed with sat. NaHCO_3_ solution (100 mL) and brine (50 mL). The aqueous layer was extracted with EtOAc (3 × 100 mL). The combined organics were dried over Na_2_SO_4_ and the solvent was evaporated under reduced pressure. The resultant crude product was purified by column chromatography (98:2→96:4→95:5, CH_2_Cl_2_-MeOH) to give the fully protected muraymycin analogue as a colorless solid. This was dissolved in aq. TFA (80%, 1.3 mL) and stirred at rt for 24 h. The reaction mixture was then diluted with water (12 mL) and the solvent was evaporated under reduced pressure. The resultant crude product was purified by semipreparative HPLC (method 1) to give **14** (bis-TFA salt) as a colorless foam (3.5 mg, 13% over 2 steps from **19**). ^1^H NMR (500 MHz, D_2_O): δ [ppm] = 0.84 (d, *J* = 6.0 Hz, 3H, Leu-5-H), 0.89 (d, *J* = 6.0 Hz, 3H, Leu-5-H), 0.90 (d, *J* = 6.6 Hz, 3H, Val-4-H), 0.93 (d, *J* = 6.9 Hz, 3H, Val-4-H), 1.35–1.46 (m, 2H, Lys-4-H), 1.50–1.69 (m, 6H, Lys-3-H_a_, Lys-5-H, Leu-3-H, Leu-4-H), 1.72–1.80 (m, 1H, Lys-3-H_b_), 1.85–1.91 (m, 2H, 2″-H), 2.10–2.17 (m, 1H, Val-3-H), 2.20 (ddd, *J* = 15.1, 10.4, 6.0 Hz, 1H, 5′-H_a_), 2.33 (ddd, *J* = 14.5, 7.3, 5.7 Hz, 1H, 2′-H_a_), 2.38 (ddd, *J* = 14.8, 6.9, 2.8 Hz 1H, 5′-H_b_), 2.45–2.51 (m, 1H, 2′-H_b_), 2.96 (t, *J* = 7.6 Hz, 2H, Lys-6-H), 3.04 (t, *J* = 7.6 Hz, 2H, 1″-H), 3.19–3.32 (m, 2H, 3″-H_a_, 3″-H_b_), 3.88 (t, *J* = 6.3 Hz, 1H, 6′-H), 4.03 (ddd, *J* = 10.4, 5.0, 2.8 Hz, 1H, 4′-H), 4.05 (d, *J* = 5.4 Hz, 1H, Val-2-H), 4.09 (dd, *J* = 8.8, 5.7 Hz, 1H, Lys-2-H), 4.23 (dd, *J* = 9.8, 4.7 Hz, 1H, Leu-2-H), 4.28–4.32 (m, 1H, 3′-H), 5.85 (d, *J* = 8.2 Hz, 1H, 5-H), 6.16 (dd, *J* = 6.6, 6.6 Hz, 1H, 1′-H), 7.66 (d, *J* = 8.2 Hz, 1H, 6-H). ^13^C NMR (126 MHz, D_2_O): δ [ppm] = 17.03 (Val-C-4), 18.56 (Val-C-4), 20.71 (Leu-C-4), 22.10 (Leu-C-4), 22.16 (Lys-C-4), 24.46 (Lys-C-5), 25.69 (C-2″), 26.32 (Leu-C-4), 30.02 (Val-C-3), 30.84 (Lys-C-3), 32.95 (C-5′), 35.99 (C-3″), 37.47 (C-2′), 39.29 (Lys-C-6), 39.56 (Leu-C-6), 44.25 (C-1″), 52.65 (Leu-C-2), 54.13 (Lys-C-2), 58.95 (Val-C-2), 59.60 (C-6′), 73.53 (C-3′), 82.56 (C-4′), 86.33 (C-1′), 102.30 (C-5), 142.87 (C-6), 151.51 (C-2), 159.56 (urea-C=O), 166.27 (C-4), 171.90 (C-7′), 174.97 (Leu-C-1), 175.48 (Lys-C-1), 176.79 (Val-C-1). ^19^F NMR (376 MHz, D_2_O): δ [ppm] = −75.59 (TFA-CF_3_). HRMS (ESI^+^): calcd.: 727.3985 [M + H]^+^, found: 727.3966. HPLC (semipreparative): t_R_ = 25.9 min (method 1).

Thymidine-derived muraymycin analogue (**15**): Nucleosyl amino acid ester **20** (25 mg, 53 μmol) was dissolved in THF (3 mL), and a solution of aldehyde **17** [42] (42 mg, 68 μmol) in THF (3 mL) was added. The reaction mixture was stirred over molecular sieve (3 Å) at rt for 24 h. NaBH(OAc)_3_ (23 mg, 0.11 mmol) and Amberlyst 15^TM^ (3.5 mg, 11 μmol) were added and stirring was continued at rt for 24 h. The reaction mixture was filtered and the residue was washed with EtOAc (150 mL). The combined filtrates were washed with sat. NaHCO_3_ solution (100 mL) and brine (50 mL). The aqueous layer was extracted with EtOAc (3 × 100 mL). The combined organics were dried over Na_2_SO_4_ and the solvent was evaporated under reduced pressure. The resultant crude product was purified by column chromatography (98:2→95:5, CH_2_Cl_2_-MeOH) to give the fully protected muraymycin analogue as a colorless solid. This was dissolved in aq. TFA (80%, 6.5 mL) and stirred at rt for 24 h. The reaction mixture was then diluted with water (12 mL) and the solvent was evaporated under reduced pressure. The resultant crude product was purified by semipreparative HPLC (method 2) to give **15** (bis-TFA salt) as a colorless foam (9.0 mg, 15% over 2 steps from **20**). ^1^H NMR (500 MHz, D_2_O): δ [ppm] = 0.83 (d, *J* = 6.0 Hz, 3H, Leu-5-H), 0.88 (d, *J* = 5.7 Hz, 3H, Leu-5-H), 0.89 (d, *J* = 6.6 Hz, 3H, Val-4-H), 0.93 (d, *J* = 6.9 Hz, 3H, Val-4-H), 1.31–1.45 (m, 2H, Lys-4-H), 1.50–1.68 (m, 6H, Lys-3-H_a_, Lys-5-H, Leu-3-H, Leu-4-H), 1.73–1.76 (m, 1H, Lys-3-H_b_), 1.85–1.90 (m, 2H, 2″-H), 1.86 (s, 3H, 7-H), 2.10–2.16 (m, 1H, Val-3-H), 2.20–2.25 (m, 1H, 5′-H_a_), 2.30 (ddd, *J* = 14.2, 6.9, 5.0 Hz, 1H, 2′-H_a_), 2.39–2.44 (m, 1H, 5′-H_b_), 2.46–2.51 (m, 1H, 2′-H_b_), 2.96 (t, *J* = 7.6 Hz, 2H, Lys-6-H), 3.06 (t, *J* = 7.3 Hz, 2H, 1″-H), 3.20–3.29 (m, 2H, 3″-H), 3.94–3.97 (m, 1H, 6′-H), 4.02 (ddd, *J* = 10.4, 4.7, 2.8 Hz, 1H, 4′-H), 4.05 (d, *J* = 5.4 Hz, 1H, Val-2-H), 4.05–4.10 (dd, *J* = 8.5, 5.4 Hz, 1H, Lys-2-H), 4.21 (dd, *J* = 9.8, 4.7 Hz, 1H, Leu-2-H), 4.30–4.33 (m, 1H, 3′-H), 6.16 (dd, *J* = 6.6, 6.6 Hz, 1H, 1′-H), 7.44 (s, 1H, 6-H). ^13^C NMR (126 MHz, D_2_O): δ [ppm] = 11.57 (C-7), 17.02 (Val-C-4), 18.54 (Val-C-4), 20.70 (Leu-C-4), 22.09 (Leu-C-4), 22.16 (Lys-C-4), 24.45 (Lys-C-5), 25.67 (C-2″), 26.32 (Leu-C-4), 30.00 (Val-C-3), 30.82 (Lys-C-3), 32.73 (C-5′), 35.96 (C-3″), 37.24 (C-2′), 39.28 (Lys-C-6), 39.55 (Leu-C-6), 44.19 (C-1″), 52.62 (Leu-C-2), 54.15 (Lys-C-2), 58.91 (Val-C-2), 59.16 (C-6′), 73.16 (C-3′), 82.30 (C-4′), 85.96 (C-1′), 111.52 (C-5), 116.41 (d, *J*_CF_ = 291.4 Hz, TFA-CF_3_), 138.34 (C-6), 151.65 (C-2), 159.56 (urea-C=O), 163.05 (d, *J*_CF_ = 35.7 Hz, TFA-COO), 166.51 (C-4), 171.57 (C-7′), 174.95 (Leu-C-1), 175.46 (Lys-C-1), 176.73 (Val-C-1). ^19^F NMR (376 MHz, D_2_O): δ [ppm] = −75.59 (TFA-CF_3_). MS (ESI^+^): *m/z* = 741.5 [M + H]^+^, 763.5 [M + Na]^+^. HRMS (ESI^+^): calcd.: 741.4141 [M + H]^+^, found: 741.4122. HPLC (semipreparative): t_R_ = 13.9 min (method 2).

5,6-Dihydrouridine-derived muraymycin analogue (**16**): Protected urea dipeptide **46** [24,41] (15.5 mg, 34.8 μmol) was dissolved in THF (5 mL), and 1-hydroxybenzotriazole (HOBt, 4.9 mg, 36 μmol), benzotriazole-1-yl-oxytripyrrolidinophosphonium hexafluorophosphate (PyBOB, 18.7 mg, 35.9 μmol) and DIPEA (12.2 μL, 71.8 μmol) were added. The reaction mixture was stirred at rt for 30 min. After cooling to 0 °C, a solution of 5,6-dihydrouridine derivative **45** (25.2 mg, 33.2 μmol) in THF (7 mL) was added. The reaction mixture was stirred at 0 °C for 30 min and at rt for 3 h. The solvent was evaporated under reduced pressure, and the resultant crude product was purified by column chromatography (98:2→95:5, CH_2_Cl_2_-MeOH) to give the fully protected muraymycin analogue. This was dissolved in aq. TFA (80%, 5 mL) and stirred at rt for 24 h. The reaction mixture was then diluted with water (15 mL) and the solvent was evaporated under reduced pressure. The resultant crude product was purified by semipreparative HPLC (method 3) to give **16** (bis-TFA salt) as a colorless solid (7.9 mg, 32% over 2 steps from **45**). ^1^H NMR (500 MHz, D_2_O, 15 °C): δ [ppm] = 0.79 (d, *J* = 5.0 Hz, 3H, Leu-5-H), 0.82–0.87 (m, 6H, Leu-5-H, Val-4-H), 0.89 (d, *J* = 6.4 Hz, 3H, Val-4-H), 1.31–1.41 (m, 2H, Lys-4-H), 1.44–1.56 (m, 3H, Leu-3-H, Leu-4-H), 1.56–1.66 (m, 2H, Lys-5-H), 1.67–1.76 (m, 2H, Lys-3-H), 1.78–1.89 (m, 2H, 2″-H), 2.04–2.16 (m, 2H, Val-3-H, 5′-H_a_), 2.29–2.37 (m, 1H, 5′-H_b_), 2.68 (t, *J* = 6.5 Hz, 2H, 5-H), 2.89–2.94 (m, 2H, Lys-6-H), 2.98–3.04 (m, 2H, 1″-H), 3.16–3.25 (m, 2H, 3″-H), 3.45 (t, *J* = 6.5 Hz, 2H, 6-H), 3.84–3.88 (m, 1H, 6′-H), 3.89–3.95 (m, 1H, 3′-H), 3.96–4.09 (m, 3H, 4′-H, Lys-2-H, Val-2-H), 4.12–4.22 (m, 1H, Leu-2-H), 4.25 (dd, *J* = 5.3, 4.9 Hz, 1H, 2′-H), 5.67 (d, *J* = 4.9 Hz, 1H, 1′-H). ^13^C NMR (126 MHz, D_2_O): δ [ppm] = 17.04 (Val-C-4), 18.56 (Val-C-4), 20.72 (Leu-C-5), 22.10 (Lys-C-4), 22.16 (Leu-C-5), 24.46 (Leu-C-4), 25.69 (C-2″), 26.32 (Lys-C-5), 30.01 (Val-C-3), 30.21 (C-5), 30.83 (Lys-C-3), 33.04 (C-5′), 36.00 (C-3″), 37.56 (C-6), 39.30 (Lys-C-6), 39.60 (Leu-C-3), 44.31 (C-1″), 52.65 (Leu-C-2), 54.11 (Lys-C-2), 58.95 (Val-C-2), 59.42 (C-6′), 70.50 (C-2′), 73.30 (C-3′), 79.07 (C-4′), 89.51 (C-1′), 116.47 (q, *J*_CF_ = 291.4 Hz, TFA-CF_3_), 154.45 (C-2), 159.58 (urea-C=O), 163.10 (q, *J*_CF_ = 35.6 Hz, TFA-COO), 171.47 (C-7′), 173.75 (C-4), 174.99 (Lys-C-1), 175.46 (Leu-C-1), 176.78 (Val-C-1). ^19^F NMR (376 MHz, D_2_O): δ [ppm] = −76.04 (TFA-CF_3_). HRMS (ESI^+^): calcd.: 745.4090 [M + H]^+^, found: 745.4074. HPLC (semipreparative): t_R_ = 5.9 min (method 3).

(6′*S*)-5-Fluorouridinyl amino acid *tert*-butyl ester (**18**): Cbz-protected nucleosyl amino acid ester **38** (25 mg, 34 μmol) was dissolved in *i*-PrOH (2 mL). 1,4-Cyclohexadiene (31 μL, 0.34 mmol) and Pd black (~4 mg, 38 μmol) were added and the reaction mixture was stirred at rt for 3 h. After filtration through a syringe filter and washing the filter with EtOAc (3 × 5 mL), the solvent of the combined filtrates was evaporated under reduced pressure to give **18** as a colorless foam (18 mg, 88%). ^1^H NMR (500 MHz, CDCl_3_): δ [ppm] = 0.08 (s, 6H, Si(CH_3_)_2_C(CH_3_)_3_), 0.12 (s, 6H, Si(CH_3_)_2_C(CH_3_)_3_), 0.90 (s, 9H, Si(CH_3_)_2_C(CH_3_)_3_), 0.91 (s, 9H, Si(CH_3_)_2_C(CH_3_)_3_), 1.47 (s, 9H, COOC(CH_3_)_3_), 1.86 (ddd, *J* = 17.0, 10.4, 6.3 Hz, 1H, 5′-H_a_), 2.18 (ddd, *J* = 14.2, 6.3, 2.8 Hz, 1H, 5′-H_b_), 3.63 (dd, *J* = 6.3, 6.3 Hz, 1H, 6′-H), 3.67 (dd, *J* = 6.6, 4.1 Hz, 1H, 3′-H), 4.16 (dd, *J* = 4.1, 2.8 Hz, 1H, 2′-H), 4.21 (ddd, *J* = 10.7, 6.6, 2.9 Hz, 1H, 4′-H), 5.63 (d, *J* = 1.9 Hz, 1H, 1′-H), 7.58 (d, *J*_HF_ = 6.0 Hz, 1H, 6-H). ^13^C NMR (126 MHz, CDCl_3_): δ [ppm] = −4.84 (Si(CH_3_)_2_C(CH_3_)_3_), −4.31 (Si(CH_3_)_2_C(CH_3_)_3_), 17.91 (Si(CH_3_)_2_C(CH_3_)_3_), 18.05 (Si(CH_3_)_2_C(CH_3_)_3_), 25.76 (Si(CH_3_)_2_C(CH_3_)_3_), 25.84 (Si(CH_3_)_2_C(CH_3_)_3_), 27.98 (COOC(CH_3_)_3_), 37.80 (C-5′), 53.18 (C-6′), 75.01 (C-2′), 75.19 (C-3′), 81.34 (C-4′), 81.74 (COOC(CH_3_)_3_), 91.56 (C-1′), 124.26 (d, *J*_CF_ = 34.8 Hz, C-6), 140.4 (d, *J*_CF_ = 237.4 Hz, C-5), 148.55 (C-2), 156.82 (d, *J*_CF_ = 26.6 Hz, C-4). ^19^F NMR (376 MHz, CDCl_3_): −164.54. MS (ESI^+^): *m/z* = 604.4 [M + H]^+^, 626.4 [M + Na]^+^. HRMS (ESI^+^): calcd.: 604.3244 [M + H]^+^, 626.3064 [M + Na]^+^, found: 604.3232, 626.3051. TLC: *R*_f_ = 0.31 (95:5, CH_2_Cl_2_-MeOH).

(6′*S*)-2′-Deoxyuridinyl amino acid *tert*-butyl ester (**19**): Cbz-protected nucleosyl amino acid ester **39** (50 mg, 85 μmol) was dissolved in *i*-PrOH (5 mL). 1,4-Cyclohexadiene (80 μL, 0.85 mmol) and Pd black (~4 mg, 38 μmol) were added and the reaction mixture was stirred at rt for 1.5 h. After filtration through a syringe filter and washing the filter with EtOAc (3 × 10 mL), the solvent of the combined filtrates was evaporated under reduced pressure to give **19** as a colorless foam (38 mg, 98%). ^1^H NMR (500 MHz, CDCl_3_): δ [ppm] = 0.08 (s, 6H, Si(CH_3_)_2_C(CH_3_)_3_), 0.89 (s, 9H, Si(CH_3_)_2_C(CH_3_)_3_), 1.47 (s, 9H, COOC(CH_3_)_3_), 1.84 (ddd, *J* = 14.2, 9.5, 6.9 Hz, 1H, 5′-H_a_), 2.11–2.17 (m, 2H, 2′-H_a_, 5′-H_b_), 2.30 (ddd, *J* = 13.6, 6.6, 5.4 Hz, 1H, 2′-H_b_), 3.57 (dd, *J* = 6.3, 6.3 Hz, 1H, 6′-H), 3.95 (ddd, *J* = 9.1, 5.0, 3.5 Hz, 1H, 4′-H), 4.11 (dd, *J* = 12.0, 5.0 Hz, 1H, 3′-H), 5.76 (d, *J* = 7.9 Hz, 1H, 5-H), 6.16 (dd, *J* = 6.0, 6.0 Hz, 1H, 1′-H), 7.44 (d, *J* = 8.2 Hz, 1H, 6-H). ^13^C NMR (126 MHz, CDCl_3_): δ [ppm] = −4.71 (Si(CH_3_)_2_C(CH_3_)_3_), 17.90 (Si(CH_3_)_2_C(CH_3_)_3_), 25.66 (Si(CH_3_)_2_C(CH_3_)_3_), 28.00 (COOC(CH_3_)_3_), 37.85 (C-5′), 40.58 (C-2′), 53.09 (C-6′), 74.89 (C-3′), 81.65 (COOC(CH_3_)_3_), 84.28 (C-4′), 85.25 (C-1′), 102.55 (C-5), 139.88 (C-6), 149.90 (C-2), 162.86 (C-4), 173.81 (C-7′). MS (ESI^+^): *m/z* = 456.1 [M + H]^+^, 478.2 [M + Na]^+^. HRMS (ESI^+^): calcd.: 456.2524 [M + H]^+^, found: 456.2498.

(6′*S*)-Thymidinyl amino acid *tert*-butyl ester (**20**): Cbz-protected nucleosyl amino acid ester **40** (50 mg, 83 μmol) was dissolved in *i*-PrOH (5 mL). 1,4-Cyclohexadiene (77 μL, 0.82 mmol) and Pd black (~4 mg, 38 μmol) were added and the reaction mixture was stirred at rt for 2.5 h. After filtration through a syringe filter and washing the filter with EtOAc (3 × 10 mL), the solvent of the combined filtrates was evaporated under reduced pressure to give **20** as a colorless foam (37 mg, 95%). ^1^H NMR (500 MHz, CDCl_3_): δ [ppm] = 0.09 (s, 6H, Si(CH_3_)_2_C(CH_3_)_3_), 0.90 (s, 9H, Si(CH_3_)_2_C(CH_3_)_3_), 1.47 (s, 9H, COOC(CH_3_)_3_), 1.95 (d, *J* = 1.1 Hz, 3H, 7-H), 1.86 (ddd, *J* = 16.1, 9.3, 6.9 Hz, 1H, 5′-H_a_), 2.13–2.18 (m, 2H, 2′-H_a_, 5′-H_b_), 2.26 (ddd, *J* = 13.6, 6.7, 5.0 Hz, 1H, 2′-H_b_), 3.58 (dd, *J* = 6.4, 6.4 Hz, 1H, 6′-H), 3.93 (ddd, *J* = 9.2, 4.9, 4.0 Hz, 1H, 4′-H), 4.13 (dd, *J* = 12.1, 5.0 Hz, 1H, 3′-H), 6.17 (dd, *J* = 6.5, 6.5 Hz, 1H, 1′-H), 7.20 (d, *J* = 1.1 Hz, 1H, 6-H). ^13^C NMR (126 MHz, CDCl_3_): δ [ppm] = −4.71 (Si(CH_3_)_2_C(CH_3_)_3_), 12.58 (C-7), 17.90 (Si(CH_3_)_2_C(CH_3_)_3_), 25.67 (Si(CH_3_)_2_C(CH_3_)_3_), 27.98 (COOC(CH_3_)_3_), 37.86 (C-5′), 40.33 (C-2′), 53.10 (C-6′), 75.00 (C-3′), 81.57 (COOC(CH_3_)_3_), 84.04 (C-4′), 84.87 (C-1′), 111.07 (C-5), 135.63 (C-6), 149.97 (C-2), 163.43 (C-4), 173.88 (C-7′). MS (ESI^+^): *m/z* = 470.1 [M + H]^+^, 492.3 [M + Na]^+^. HRMS (ESI^+^): calcd.: 470.2681 [M + H]^+^, found: 470.2658.

2′,3′,5′-Tris-*O*-TBDMS-5-fluorouridine (**24**): 5-Fluorouridine **21** (2.00 g, 7.62 mmol) was coevaporated with dry pyridine (2 × 14 mL). Pyridine (26 mL), imidazole (1.56 g, 22.8 mmol) and TBDMSCl (5.74 g, 38.1 mmol) were added and the reaction mixture was stirred at rt for 4 d. Water (90 mL) was then added at 0 °C. The solvent was evaporated under reduced pressure, the resultant colorless residue was dissolved in EtOAc (100 mL) and washed with sat. NaHCO_3_ solution (3 × 100 mL) and brine (2 × 20 mL). The organic layer was dried over Na_2_SO_4_ and the solvent was evaporated under reduced pressure. The resultant crude product was purified by column chromatography (7:3, petroleum ether-EtOAc) to give **24** as a colorless foam (2.87 g, 62%). ^1^H NMR (500 MHz, CDCl_3_): δ [ppm] = 0.07 (s, 6H, Si(CH_3_)_2_C(CH_3_)_3_), 0.09 (s, 6H, Si(CH_3_)_2_C(CH_3_)_3_), 0.16 (s, 6H, Si(CH_3_)_2_C(CH_3_)_3_), 0.89 (s, 9H, Si(CH_3_)_2_C(CH_3_)_3_), 0.92 (s, 9H, Si(CH_3_)_2_C(CH_3_)_3_), 0.97 (s, 9H, Si(CH_3_)_2_C(CH_3_)_3_), 3.77 (dd, *J* = 11.7, 1.0 Hz, 1H, 5′-H_a_), 4.01 (dd, *J* = 11.7, 1.2 Hz, 1H, 5′-H_b_), 4.06–4.10 (m, 3H, 2′-H, 3′-H, 4′-H), 5.89 (dd, *J* = 4.1, 1.2 Hz, 1H, 1′-H), 8.18 (d, *J*_HF_ = 6.3 Hz, 1H, 6-H), 8.73 (bs, 1H, 3-NH). ^13^C NMR (126 MHz, CDCl_3_): δ [ppm] = −5.54 (Si(CH_3_)_2_C(CH_3_)_3_), −4.81 (Si(CH_3_)_2_C(CH_3_)_3_), −3.94 (Si(CH_3_)_2_C(CH_3_)_3_), 17.92 (Si(CH_3_)_2_C(CH_3_)_3_), 18.05 (Si(CH_3_)_2_C(CH_3_)_3_), 18.61 (Si(CH_3_)_2_C(CH_3_)_3_), 25.70 (Si(CH_3_)_2_C(CH_3_)_3_), 25.79 (Si(CH_3_)_2_C(CH_3_)_3_), 26.06 (Si(CH_3_)_2_C(CH_3_)_3_), 62.15 (C-5′), 71.16 (C-3′), 76.12 (C-4′), 85.11 (C-2′), 88.78 (C-1′), 124.41 (d, *J*_CF_ = 33.9 Hz, C-6), 140.37 (d, *J*_CF_ = 236.4 Hz, C-5), 148.65 (C-2), 156.67 (d, *J*_CF_ = 26.6 Hz, C-4). ^19^F NMR (376 MHz, CDCl_3_): δ [ppm] = −164.60. MS (ESI^−^): *m/z* = 605.3 [M + H]^+^, 627.3 [M + Na]^+^. HRMS (ESI)^+^: calcd.: 605.3268 [M + H]^+^, found: 605.3261. TLC: *R*_f_ = 0.85 (1:1, petroleum ether-EtOAc).

3′,5′-Bis-*O*-TBDMS-2′-deoxyuridine (**25**): 2′-Deoxyuridine **22** (5.09 g, 22.3 mmol) was coevaporated with dry pyridine (2 × 40 mL). Pyridine (75 mL), imidazole (4.58 g, 66.9 mmol) and TBDMSCl (10.1 g, 66.9 mmol) were added and the reaction mixture was stirred at rt for 31 h. Water (50 mL) was then added at 0 °C. The solvent was evaporated under reduced pressure, the resultant colorless residue was dissolved in EtOAc (250 mL) and washed with sat. NaHCO_3_ solution (3 × 125 mL) and brine (50 mL). The organic layer was dried over Na_2_SO_4_ and the solvent was evaporated under reduced pressure. The resultant crude product was purified by column chromatography (3:2, petroleum ether-EtOAc) to give **25** as a colorless solid (9.90 g, 97%). ^1^H NMR (300 MHz, CDCl_3_): δ [ppm] = 0.08 (s, 3H, Si(CH_3_)_2_C(CH_3_)_3_), 0.09 (s, 3H, Si(CH_3_)_2_C(CH_3_)_3_), 0.11 (s, 3H, Si(CH_3_)_2_C(CH_3_)_3_), 0.11 (s, 3H, Si(CH_3_)_2_C(CH_3_)_3_), 0.90 (s, 9H, Si(CH_3_)_2_C(CH_3_)_3_), 0.98 (s, 9H, Si(CH_3_)_2_C(CH_3_)_3_), 2.07 (ddd, *J* = 13.2, 6.2, 6.2 Hz, 1H, 2′-H_a_), 2.33 (ddd, *J* = 13.2, 6.2, 4.2 Hz, 1H, 2′-H_b_), 3.77 (dd, *J* = 11.3, 2.5 Hz, 1H, 5′-H_a_), 3.90 (dd, *J* = 11.3, 2.5 Hz, 1H, 5′-H_b_), 3.92–3.94 (m, 1H, 4′-H), 4.42 (ddd, *J* = 6.0, 6.0, 3.8 Hz, 1H, 3′-H), 5.68 (d, *J* = 8.0 Hz, 1H, 5-H), 6.29 (dd, *J* = 6.2, 6.2 Hz, 1H, 1′-H), 7.47 (d, *J* = 8.0 Hz, 1H, 6-H), 8.16 (s, 1H, 3-NH). ^13^C NMR (126 MHz, CDCl_3_): δ [ppm] = −5.43 (Si(CH_3_)_2_C(CH_3_)_3_), −5.36 (Si(CH_3_)_2_C(CH_3_)_3_), −4.72 (Si(CH_3_)_2_C(CH_3_)_3_), −4.47 (Si(CH_3_)_2_C(CH_3_)_3_), 18.12 (Si(CH_3_)_2_C(CH_3_)_3_), 18.50 (Si(CH_3_)_2_C(CH_3_)_3_), 25.86 (Si(CH_3_)_2_C(CH_3_)_3_), 26.02 (Si(CH_3_)_2_C(CH_3_)_3_), 42.00 (C-2′), 62.60 (C-5′), 71.38 (C-3′), 85.35 (C-1′), 87.94 (C-4′), 102.25 (C-5), 140.34 (C-6), 150.10 (C-2), 162.95 (C-4). HRMS (CI): calcd.: 457.2549 [M + H]^+^, found: 457.2538. TLC: *R*_f_ = 0.40 (1:1, CH_2_Cl_2_-EtOAc).

3′,5′-Bis-*O*-TBDMS-thymidine (**26**): Thymidine **23** (5.40 g, 22.3 mmol) was coevaporated with dry pyridine (2 × 22 mL). Pyridine (50 mL), imidazole (3.88 g, 57.0 mmol) and TBDMSCl (8.43 g, 56.0 mmol) were added and the reaction mixture was stirred at rt for 48 h. Water (50 mL) was then added at 0 °C. The solvent was evaporated under reduced pressure, the resultant colorless residue was dissolved in EtOAc (250 mL) and washed with sat. NaHCO_3_ solution (3 × 125 mL) and brine (50 mL). The organic layer was dried over Na_2_SO_4_ and the solvent was evaporated under reduced pressure. The resultant crude product was purified by column chromatography (3:1, petroleum ether-EtOAc) to give **26** as a colorless solid (9.81 g, 94%). ^1^H NMR (300 MHz, CDCl_3_): δ [ppm] = 0.08 (s, 3H, Si(CH_3_)_2_C(CH_3_)_3_), 0.08 (s, 3H, Si(CH_3_)_2_C(CH_3_)_3_), 0.11 (s, 3H, Si(CH_3_)_2_C(CH_3_)_3_), 0.11 (s, 3H, Si(CH_3_)_2_C(CH_3_)_3_), 0.90 (s, 9H, Si(CH_3_)_2_C(CH_3_)_3_), 0.93 (s, 9H, Si(CH_3_)_2_C(CH_3_)_3_), 1.92 (d, *J* = 0.8 Hz, 3H, 7-H), 1.95–2.05 (m, 1H, 2′-H_a_), 2.25 (ddd, *J* = 13.1, 5.8, 2.5 Hz, 1H, 2′-H_b_) 3.76 (dd, *J* = 11.4, 2.5 Hz, 1H, 5′-H_a_), 3.87 (dd, *J* = 11.4, 2.5 Hz, 1H, 5′-H_b_), 3.94 (dd, *J* = 2.5, 2.5 Hz, 1H, 4′-H), 4.41 (ddd, *J* = 5.8, 2.5, 2.5 Hz, 1H, 3′-H), 6.33 (dd, *J* = 8.0, 5.8 Hz, 1H, 1′-H), 7.47 (d, *J* = 0.8 Hz, 1H, 6-H), 8.03 (s, 1H, 3-NH). ^13^C NMR (126 MHz, CDCl_3_): δ [ppm] = −5.29 (Si(CH_3_)_2_C(CH_3_)_3_), −5.20 (Si(CH_3_)_2_C(CH_3_)_3_), −4.67 (Si(CH_3_)_2_C(CH_3_)_3_), −4.48 (Si(CH_3_)_2_C(CH_3_)_3_), 12.68 (C-7), 18.17 (Si(CH_3_)_2_C(CH_3_)_3_), 18.57 (Si(CH_3_)_2_C(CH_3_)_3_), 25.90 (Si(CH_3_)_2_C(CH_3_)_3_), 26.09 (Si(CH_3_)_2_C(CH_3_)_3_), 41.53 (C-2′), 63.16 (C-5′), 72.45 (C-3′), 85.00 (C-1′), 88.01 (C-4′), 110.93 (C-5), 135.64 (C-6), 150.17 (C-2), 163.52 (C-4). HRMS (CI): calcd.: 471.2705 [M + H]^+^, found: 471.2694. TLC: *R*_f_ = 0.29 (1:1, CH_2_Cl_2_-EtOAc).

2′,3′-Bis-*O*-TBDMS-5-fluorouridine (**27**): 2′,3′,5′-Tris-*O*-TBDMS-5-fluorouridine **24** (1.09 g, 1.80 mmol) was dissolved in THF (22 mL) and aq. TFA (50%, 5.4 mL) was added slowly at 0 °C over 40 min. The reaction mixture was stirred at 0 °C for 5.5 h. After conversion was sufficient according to TLC, sat. NaHCO_3_ solution (70 mL) was added until pH 8 was reached. The aqueous layer was extracted with EtOAc (3 × 70 mL) and the combined organics were dried over Na_2_SO_4_. The solvent was evaporated under reduced pressure, and the resultant crude product was purified by column chromatography (7:3, petroleum ether-EtOAc) to give **27** as a colorless foam (610 mg, 69%). ^1^H NMR (500 MHz, CDCl_3_): δ [ppm] = 0.09 (s, 6H, Si(CH_3_)_2_C(CH_3_)_3_), 0.10 (s, 6H, Si(CH_3_)_2_C(CH_3_)_3_), 0.90 (s, 9H, Si(CH_3_)_2_C(CH_3_)_3_), 0.92 (s, 9H, Si(CH_3_)_2_C(CH_3_)_3_), 2.45 (bs, 1H, 5′-OH), 3.80 (ddd, *J* = 11.9, 5.0, 1.9 Hz, 1H, 5′-H_a_), 4.04 (ddd, *J* = 11.6, 2.2 Hz, 1H, 5′-H_b_), 4.11 (d, *J* = 5.0 Hz, 1H, 4′-H), 4.16 (dd, *J* = 4.7, 4.4 Hz, 1H, 3′-H), 4.34 (dd, *J* = 4.7, 4.4 Hz, 1H, 2′-H), 5.60 (d, *J* = 6.0 Hz, 1H, 1′-H), 8.12 (d, *J*_HF_ = 6.6 Hz, 1H, 6-H), 9.00 (bs, 1H, 3-NH). ^13^C NMR (126 MHz, CDCl_3_): δ [ppm] = −4.84 (Si(CH_3_)_2_C(CH_3_)_3_), −4.55 (Si(CH_3_)_2_C(CH_3_)_3_), 17.95 (Si(CH_3_)_2_C(CH_3_)_3_), 18.05 (Si(CH_3_)_2_C(CH_3_)_3_), 25.75 (Si(CH_3_)_2_C(CH_3_)_3_), 25.80 (Si(CH_3_)_2_C(CH_3_)_3_), 60.89 (C-5′), 70.76 (C-4′), 74.87 (C-2′), 84.88 (C-3′), 91.89 (C-1′), 126.12 (d, *J*_CF_ = 34.8 Hz, C-6), 139.29 (d, *J*_CF_ = 236.4 Hz, C-5), 148.76 (C-2), 156.78 (d, *J*_CF_ = 26.6 Hz, C-4). ^19^F NMR (376 MHz, CDCl_3_): δ [ppm] = −165.47. MS (ESI^+^): *m/z* = 513.2 [M + Na]^+^. HRMS (ESI^+^): calcd.: 491.2403 [M + H]^+^, 513.2223 [M + Na]^+^, found: 491.2392, 513.2210. TLC: *R*_f_ = 0.19 (7:3, petroleum ether-EtOAc).

3′-*O*-TBDMS-2′-deoxyuridine (**28**): 3′,5′-Bis-*O*-TBDMS-2′-deoxyuridine **25** (7.09 g, 15.5 mmol) was dissolved in MeOH (200 mL) and cooled to −10 °C. Acetyl chloride (273 μL, 3.84 mmol) was added slowly at this temperature. The mixture was warmed to 0 °C and stirred at this temperature for 2.5 h. After conversion was sufficient according to TLC, sat. NaHCO_3_ solution (150 mL) was added and the mixture was stirred for 15 min. Then EtOAc (300 mL), water (100 mL) and brine (200 mL) were added. The organic layer was washed with sat. NaHCO_3_ solution (100 mL), brine (2 x 100 mL) and water (100 mL) and dried over Na_2_SO_4_. The solvent was evaporated under reduced pressure, and the resultant crude product was purified by column chromatography (3:2, petroleum ether-EtOAc→7:3, petroleum ether-EtOAc→EtOAc) to give **28** as a colorless solid (3.47 g, 65%). ^1^H NMR (500 MHz, CDCl_3_): δ [ppm] = 0.08 (s, 6H, Si(CH_3_)_2_C(CH_3_)_3_), 0.89 (s, 9H, Si(CH_3_)_2_C(CH_3_)_3_), 2.26–2.30 (m, 2H, 2′-H), 2.51 (dd, *J* = 4.6, 4.6 Hz, 1H, 5′-OH), 3.76 (ddd, *J* = 11.6, 5.8, 3.2 Hz, 1H, 5′-H_a_), 3.89–3.91 (m, 1H, 5′-H_b_), 3.92–3.94 (m, 1H, 4′-H), 4.48 (ddd, *J* = 5.9, 4.2, 4.2 Hz, 1H, 3′-H), 5.73 (d, *J* = 8.1 Hz, 1H, 5-H), 6.18 (dd, *J* = 6.6, 6.6 Hz, 1H, 1′-H), 7.68 (d, *J* = 8.1 Hz, 1H, 6-H), 9.33 (s, 1H, 3-NH). ^13^C NMR (126 MHz, CDCl_3_): δ [ppm] = −4.90 (Si(CH_3_)_2_C(CH_3_)_3_), −4.74 (Si(CH_3_)_2_C(CH_3_)_3_), 17.92 (Si(CH_3_)_2_C(CH_3_)_3_), 25.67 (Si(CH_3_)_2_C(CH_3_)_3_), 40.87 (C-2′), 61.73 (C-5′), 71.38 (C-3′), 86.60 (C-1′), 87.61 (C-4′), 102.40 (C-5), 141.15 (C-6), 150.30 (C-2), 163.56 (C-4). HRMS (CI): calcd.: 343.1684 [M + H]^+^, found: 343.1687. TLC: *R*_f_ = 0.44 (EtOAc).

3′-*O*-TBDMS-thymidine (**29**): 3′,5′-Bis-*O*-TBDMS-thymidine **26** (7.02 g, 14.9 mmol) was dissolved in MeOH (340 mL) and cooled to −10 °C. Acetyl chloride (265 μL, 3.73 mmol) was added slowly at this temperature. The mixture was warmed to 0 °C and stirred at this temperature for 3.5 h. After conversion was sufficient according to TLC, sat. NaHCO_3_ solution (150 mL) was added and the mixture was stirred for 15 min. Then EtOAc (300 mL), water (100 mL) and brine (400 mL) were added. The organic layer was washed with sat. NaHCO_3_ solution (2 × 100 mL), water (100 mL) and brine (100 mL) and dried over Na_2_SO_4_. The solvent was evaporated under reduced pressure, and the resultant crude product was purified by column chromatography (1:1, petroleum ether-EtOAc) to give **29** as a colorless solid (3.21 g, 59%). ^1^H NMR (500 MHz, CDCl_3_): δ [ppm] = 0.09 (s, 6H, Si(CH_3_)_2_C(CH_3_)_3_), 0.90 (s, 9H, Si(CH_3_)_2_C(CH_3_)_3_), 1.92 (d, *J* = 1.1 Hz, 3H, 7-H), 2.22 (ddd, *J* = 13.3, 6.4, 3.8 Hz, 1H, 2′-H_a_), 2.33–2.42 (m, 1H, 2′-H_b_), 2.60 (brs, 1H, 5′-OH), 3.76 (ddd, *J* = 12.4, 6.5, 3.5 Hz, 1H, 5′-H_a_), 3.90–3.95 (m, 2H, 4′-H, 5′-H_b_), 4.48–4.52 (m, 1H, 3′-H), 6.14 (dd, *J* = 6.8, 6.8 Hz, 1H, 1′-H), 7.36 (d, *J* = 1.1 Hz, 1H, 6-H), 8.63 (s, 1H, 3-NH). ^13^C NMR (126 MHz, CDCl_3_): δ [ppm] = −4.87 (Si(CH_3_)_2_C(CH_3_)_3_), −4.71 (Si(CH_3_)_2_C(CH_3_)_3_), 12.52 (C-7), 17.94 (Si(CH_3_)_2_C(CH_3_)_3_), 25.70 (Si(CH_3_)_2_C(CH_3_)_3_), 40.41 (C-2′), 62.05 (C-5′), 71.60 (C-3′), 87.09 (C-1′), 87.56 (C-4′), 111.00 (C-5), 137.05 (C-6), 150.24 (C-2), 163.59 (C-4). HRMS (CI): calcd.: 357.1840 [M + H]^+^, found: 357.1848. TLC: *R*_f_ = 0.35 (1:1, petroleum ether-EtOAc).

2′,3′-Bis-*O*-TBDMS-5-fluorouridine-5′-aldehyde (**30**): 2′,3′-Bis-*O*-TBDMS-5-fluorouridine **27** (992 mg, 2.02 mmol) was dissolved in MeCN (21 mL) and IBX (1.19 g, 4.26 mmol) was added. The reaction mixture was stirred under reflux for 1.5 h. After cooling to 0 °C, stirring was continued for 30 min. The reaction mixture was then filtered and the solid residue was washed with EtOAc (6 × 40 mL). The solvent of the combined filtrates was evaporated under reduced pressure to give **30** as a colorless foam (910 mg, 92%). This material was used in the following step without further purification and characterization with respect to its limited stability. ^1^H NMR (500 MHz, CDCl_3_): δ [ppm] = 0.04 (s, 6H, Si(CH_3_)_2_C(CH_3_)_3_), 0.14 (s, 6H, Si(CH_3_)_2_C(CH_3_)_3_), 0.89 (s, 9H, Si(CH_3_)_2_C(CH_3_)_3_), 0.94 (s, 9H, Si(CH_3_)_2_C(CH_3_)_3_), 4.18 (dd, *J* = 5.4, 4.1 Hz, 1H, 4′-H), 4.21 (dd, *J* = 4.1, 4.1 Hz, 1H, 3′-H), 4.64 (d, *J* = 3.5 Hz, 1H, 2′-H), 5.82 (dd, *J* = 5.4, 1.0 Hz, 1H, 1′-H), 8.02 (d, *J*_HF_ = 6.0 Hz, 1H, 6-H), 8.53 (brs, 1H, 3-NH), 9.83 (s, 1H, 5’-H).

3′-*O*-TBDMS-2′-deoxyuridine-5′-aldehyde (**31**): 3′-*O*-TBDMS-2′-deoxyuridine **28** (1.25 g, 3.66 mmol) was dissolved in MeCN (35 mL) and IBX (2.56 g, 9.15 mmol) was added. The reaction mixture was stirred under reflux for 2 h. After cooling to 0 °C, stirring was continued for 30 min. The reaction mixture was then filtered and the solid residue was washed with EtOAc (3 × 50 mL). The solvent of the combined filtrates was evaporated under reduced pressure to give **31** as a colorless foam (1.25 g, quant.). This material was used in the following step without further purification and characterization with respect to its limited stability. ^1^H NMR (500 MHz, CDCl_3_): δ [ppm] = 0.13 (s, 3H, Si(CH_3_)_2_C(CH_3_)_3_), 0.14 (s, 3H, Si(CH_3_)_2_C(CH_3_)_3_), 0.91 (s, 9H, Si(CH_3_)_2_C(CH_3_)_3_), 1.97–2.03 (m, 1H, 2′-H_a_), 2.37 (ddd, *J* = 13.6, 5.8, 2.2 Hz, 1H, 2′-H_b_), 4.52 (d, *J* = 2.0 Hz, 1H, 4′-H), 4.64–4.68 (m, 1H, 3′-H), 5.81 (d, *J* = 8.2 Hz, 1H, 5-H), 6.31 (dd, *J* = 8.1, 5.8 Hz, 1H, 1′-H), 7.85 (d, *J* = 8.2 Hz, 1H, 6-H), 9.26 (brs, 1H, 3-NH), 9.75 (s, 1H, 5′-H).

3′-*O*-TBDMS-thymidine-5′-aldehyde (**32**): 3′-*O*-TBDMS-thymidine **29** (1.32 g, 3.70 mmol) was dissolved in MeCN (35 mL) and IBX (2.59 g, 9.25 mmol) was added. The reaction mixture was stirred under reflux for 1 h. After cooling to 0 °C, stirring was continued for 30 min. The reaction mixture was then filtered and the solid residue was washed with EtOAc (3 × 40 mL). The solvent of the combined filtrates was evaporated under reduced pressure to give **32** as a colorless foam (1.31 g, quant.). This material was used in the following step without further purification and characterization with respect to its limited stability. ^1^H NMR (500 MHz, CDCl_3_): δ [ppm] = 0.15 (s, 3H, Si(CH_3_)_2_C(CH_3_)_3_), 0.16 (s, 3H, Si(CH_3_)_2_C(CH_3_)_3_), 0.94 (s, 9H, Si(CH_3_)_2_C(CH_3_)_3_), 1.98 (d, *J* = 1.1 Hz, 3H, 7-H), 2.03–2.12 (m, 1H, 2′-H_a_), 2.34 (ddd, *J* = 13.4, 5.9, 1.9 Hz, 1H, 2′-H_b_), 4.50 (d, *J* = 2.1 Hz, 1H, 4′-H), 4.69 (ddd, *J* = 5.2, 2.1, 1.9 Hz, 1H, 3′-H), 6.33 (dd, *J* = 8.1, 5.9 Hz, 1H, 1′-H), 7.59 (d, *J* = 1.1 Hz, 1H, 6-H), 8.76 (brs, 1H, 3-NH), 9.78 (s, 1H, 5′-H).

*Z*-6′-*N*-Cbz-5′,6′-didehydro-5-fluorouridinyl amino acid *tert*-butyl ester (**34**): THF (8 mL) was added to a solution of KHMDS in toluene (0.5 M, 2.84 mL, 1.42 mmol). After cooling to –86 °C, a solution of phosphonate **33** [31] (480 mg, 1.42 mmol) in THF (10 mL) was added slowly at this temperature. The mixture was stirred at –86 °C for 30 min. Subsequently, a solution of aldehyde **30** (900 mg, 1.85 mmol) in THF (12 mL) was added dropwise. Stirring was continued for 16 h while the solution was allowed to slowly warm to rt. After cooling to 10 °C, MeOH (3.2 mL) was added. The mixture was diluted with EtOAc (70 mL) and washed with half-sat. NaCl solution (2 × 65 mL). The organic layer was dried over Na_2_SO_4_ and the solvent was evaporated under reduced pressure. The resultant crude product was purified by threefold column chromatography (7:3, petroleum ether-EtOAc and 3:1, petroleum ether-EtOAc, respectively) to give **34** (9:1 mixture of *Z* and *E* isomers as determined by ^1^H NMR spectroscopy) as a colorless foam (184 mg, 14%). *Z*-**34**: ^1^H NMR (500 MHz, CDCl_3_): δ [ppm] = 0.07 (s, 6H, Si(CH_3_)_2_C(CH_3_)_3_), 0.12 (s, 6H, Si(CH_3_)_2_C(CH_3_)_3_), 0.90 (s, 9H, Si(CH_3_)_2_C(CH_3_)_3_), 0.91 (s, 9H, Si(CH_3_)_2_C(CH_3_)_3_), 1.49 (s, 9H, COOC(CH_3_)_3_), 3.90 (dd, *J* = 3.8, 3.8 Hz, 1H, 3′-H), 4.28 (dd, *J* = 3.5, 3.5 Hz, 1H, 2′-H), 4.92 (dd, *J* = 7.0, 7.0 Hz, 1H, 4′-H), 5.13–5.18 (m, 2H, 1″-H), 5.60 (d, *J* = 2.9 Hz, 1H, 1′-H), 6.24 (d, *J* = 7.8 Hz, 1H, 5′-H), 6.80 (brs, 1H, 6′-NH), 7.34–7.36 (m, 5H, aryl-H), 7.39 (d, *J*_HF_ = 6.0 Hz, 1H, 6-H), 8.48 (d, *J*_HF_ = 4.4 Hz, 1H, 3-NH). ^13^C NMR (126 MHz, CDCl_3_): δ [ppm] = −4.81 (Si(CH_3_)_2_C(CH_3_)_3_), −4.47 (Si(CH_3_)_2_C(CH_3_)_3_), 18.02 (Si(CH_3_)_2_C(CH_3_)_3_), 18.10 (Si(CH_3_)_2_C(CH_3_)_3_), 25.75 (Si(CH_3_)_2_C(CH_3_)_3_), 25.82 (Si(CH_3_)_2_C(CH_3_)_3_), 27.84 (COOC(CH_3_)_3_), 67.60 (C-1″), 74.90 (C-2′), 76.03 (C-3′), 79.44 (C-4′), 82.90 (COOC(CH_3_)_3_), 92.21 (C-1′), 124.28 (C-5′), 124.55 (d, *J*_CF_ = 34.8 Hz, C-6), 128.20 (C-3″/C-4″), 128.52 (C-3″/C-4″), 131.28 (C-2″), 135.71 (C-5″), 140.26 (d, *J*_CF_ = 238.3 Hz, C-5), 148.09 (C-2), 156.19 (d, *J*_CF_ = 27.5 Hz, C-4). ^19^F NMR (376 MHz, CDCl_3_): δ [ppm] = −164.45. MS (ESI^+^): *m/z* = 736.4 [M + H]^+^, 758.5 [M + Na]^+^. HRMS (ESI^+^): calcd.: 736.3455 [M + H]^+^, 758.3275 [M + Na]^+^, found: 736.3433, 758.3256. TLC: *R*_f_ = 0.60 (1:1, petroleum ether-EtOAc). *E*-**29**: ^1^H NMR (500 MHz, CDCl_3_): δ [ppm] = 0.07 (s, 6H, Si(CH_3_)_2_C(CH_3_)_3_), 0.12 (s, 6H, Si(CH_3_)_2_C(CH_3_)_3_), 0.90 (s, 9H, Si(CH_3_)_2_C(CH_3_)_3_), 0.91 (s, 9H, Si(CH_3_)_2_C(CH_3_)_3_), 1.49 (s, 9H, COOC(CH_3_)_3_), 3.84 (dd, *J* = 5.7, 4.1 Hz, 1H, 3′-H), 4.23 (dd, *J* = 3.8, 3.5 Hz, 1H, 2′-H), 5.16 (d, *J* = 1.3 Hz, 2H, 1″-H), 5.46 (dd, *J* = 9.8, 6.0 Hz, 1H, 4′-H), 5.68 (d, *J* = 4.1 Hz, 1H, 1′-H), 6.86 (d, *J* = 9.5 Hz, 1H, 5′-H), 7.26–7.38 (m, 5H, aryl-H), 7.55 (d, *J*_HF_ = 6.3 Hz, 1H, 6-H). ^19^F NMR (376 MHz, CDCl_3_): δ [ppm] = −164.09. MS (ESI^+^): *m/z* = 736.4 [M + H]^+^, 758.5 [M + Na]^+^. TLC: *R*_f_ = 0.66 (1:1, petroleum ether-EtOAc).

*Z*-6′-*N*-Cbz-5′,6′-didehydro-2′-deoxyuridinyl amino acid *tert*-butyl ester (**35**): KO*t*-Bu (460 mg, 4.06 mmol) was dissolved in THF (15 mL). After cooling to −87 °C, a solution of phosphonate **33** [31] (1.50 g, 4.02 mmol) in THF (30 mL) was added slowly at this temperature. The mixture was stirred at −86 °C for 30 min. Subsequently, a solution of aldehyde **31** (1.25 g, 3.66 mmol) in THF (12 mL) was added dropwise. Stirring was continued for 18.5 h while the solution was allowed to slowly warm to rt. After cooling to 0 °C, MeOH (5 mL) was added and the mixture was stirred for further 40 min. It was then diluted with EtOAc (150 mL) and washed with half-sat. NaCl solution (3 × 100 mL). The organic layer was dried over Na_2_SO_4_ and the solvent was evaporated under reduced pressure. The resultant crude product was purified by column chromatography (1:1, petroleum ether-EtOAc) to give **35** as a colorless foam (1.54 g, 72%). ^1^H NMR (500 MHz, CDCl_3_): δ [ppm] = 0.08 (s, 3H, Si(CH_3_)_2_C(CH_3_)_3_), 0.09 (s, 3H, Si(CH_3_)_2_C(CH_3_)_3_), 0.88 (s, 9H, Si(CH_3_)_2_C(CH_3_)_3_), 1.47 (s, 9H, COOC(CH_3_)_3_), 2.16 (ddd, *J* = 13.6, 6.2, 6.2 Hz, 1H, 2′-H_a_), 2.41 (ddd, *J* = 13.6, 6.2, 4.7 Hz, 1H, 2′-H_b_), 4.33 (ddd, *J* = 6.2, 4.7, 4.6 Hz, 1H, 3′-H), 4.71 (dd, *J* = 7.8, 4.6 Hz, 1H, 4′-H), 5.14 (d, *J* = 4.0 Hz, 2H, 1″-H), 5.73 (dd, *J* = 8.1, 2.2 Hz, 1H, 5-H), 6.13 (d, *J* = 7.8 Hz, 1H, 5′-H), 6.14 (dd, *J* = 6.2, 6.2 Hz, 1H, 1′-H), 6.78 (s, 1H, 6′-NH), 7.29-7.40 (m, 6H, aryl-H, 6-H), 8.75 (brs, 1H, 3-NH). ^13^C NMR (126 MHz, CDCl_3_): δ [ppm] = −4.76 (Si(CH_3_)_2_C(CH_3_)_3_), −4.64 (Si(CH_3_)_2_C(CH_3_)_3_), 18.06 (Si(CH_3_)_2_C(CH_3_)_3_), 25.81 (Si(CH_3_)_2_C(CH_3_)_3_), 28.03 (COOC(CH_3_)_3_), 40.88 (C-2′), 67.75 (C-1″), 75.97 (C-3′), 82.85 (C-4′), 82.92 (COOC(CH_3_)_3_), 86.69 (C-1′), 102.60 (C-5), 124.91 (C-5′), 128.40 (C-3″,C-7″), 128.51 (C-5″), 128.68 (C-4″, C-6″), 130.36 (C-6′), 135.85 (C-2″), 139.78 (C-6), 149.98 (C-2), 153.70 (Cbz-C=O), 162.83 (C-4), 171.13 (C-7′). HRMS (CI): calcd.: 588.2736 [M + H]^+^, found: 588.2717. TLC: *R*_f_ = 0.26 (EtOAc).

*Z*-6′-*N*-Cbz-5′,6′-didehydro-thymidinyl amino acid *tert*-butyl ester (**36**): KO*t*-Bu (466 mg, 4.06 mmol) was dissolved in THF (35 mL). After cooling to −80 °C, a solution of phosphonate **33** [31] (1.52 g, 4.06 mmol) in THF (30 mL) was added slowly at this temperature. The mixture was stirred at –86 °C for 30 min. Subsequently, a solution of aldehyde **32** (1.31 g, 3.70 mmol) in THF (12 mL) was added dropwise. Stirring was continued for 21 h while the solution was allowed to slowly warm to rt. After cooling to 0 °C, MeOH (5 mL) was added. The mixture was diluted with EtOAc (150 mL) and washed with half-sat. NaCl solution (3 × 100 mL). The organic layer was dried over Na_2_SO_4_ and the solvent was removed under reduced pressure. The resultant crude product was purified by twofold column chromatography (3:2, petroleum ether-EtOAc) to give **36** (95:5 mixture of *Z* and *E* isomers as determined by ^1^H NMR spectroscopy) as a colorless foam (1.71 g, 77%). ^1^H NMR (300 MHz, CDCl_3_): δ [ppm] = 0.09 (s, 3H, Si(CH_3_)_2_C(CH_3_)_3_), 0.09 (s, 3H, Si(CH_3_)_2_C(CH_3_)_3_), 0.89 (s, 9H, Si(CH_3_)_2_C(CH_3_)_3_), 1.47 (s, 9H, COOC(CH_3_)_3_), 1.92 (s, 3H, 7-H), 2.16 (ddd, *J* = 13.5, 6.2, 6.2 Hz, 1H, 2′-H_a_), 2.36 (ddd, *J* = 13.5, 6.2, 4.7 Hz, 1H, 2′-H_b_), 4.35 (ddd, *J* = 6.2, 4.7, 4.6 Hz, 1H, 3′-H), 4.70 (dd, *J* = 7.8, 4.6 Hz, 1H, 4′-H), 5.14 (d, *J* = 4.0 Hz, 2H, 1″-H), 6.13 (d, *J* = 7.8 Hz, 1 H, 5′-H), 6.14 (dd, *J* = 6.2, 6.2 Hz, 1H, 1′-H), 6.81 (s, 1H, 6′-NH), 7.13 (s, 1H, 6-H), 7.29–7.40 (m, 5H, aryl-H), 8.71 (s, 1H, 3-NH). ^13^C NMR (126 MHz, CDCl_3_): δ [ppm] = −4.76 (Si(CH_3_)_2_C(CH_3_)_3_), −4.64 (Si(CH_3_)_2_C(CH_3_)_3_), 12.78 (C-7), 18.05 (Si(CH_3_)_2_C(CH_3_)_3_), 25.81 (Si(CH_3_)_2_C(CH_3_)_3_), 28.02 (COOC(CH_3_)_3_), 40.57 (C-2′), 67.73 (C-1″), 76.13 (C-3′), 82.85 (COOC(CH_3_)_3_), 83.34 (C-4′), 86.39 (C-1′), 111.20 (C-5), 124.95 (C-5′), 128.38 (C-3″, C-7″), 128.49 (C-5″), 128.68 (C-4″, C-6″), 130.67 (C-6′), 135.61 (C-6), 135.86 (C-2″), 150.12 (C-2), 153.71 (Cbz-C=O), 162.91 (C-4), 163.67 (C-7′). HRMS (CI): calcd.: 602.2892 [M + H]^+^, found: 602.2888. TLC: *R*_f_ = 0.31 (55:45, petroleum ether-EtOAc).

(6′*S*)-6′-*N*-Cbz-5-fluorouridinyl amino acid *tert*-butyl ester (**38**): Under strict exclusion of oxygen, didehydro amino acid **34** (9:1 mixture of *Z* and *E* isomers, 280 mg, 0.381 mmol) was dissolved in MeOH (13 mL). After addition of (*S*,*S*)-Me-DUPHOS-Rh **37** (~8 mg, 12 μmol), the solution was stirred under an H_2_ atmosphere (~1 bar) at rt for 6 d. The reaction was monitored by ^1^H NMR spectroscopy. Additional catalyst **37** (~4 mg, 6 μmol) was added after further 5 d and 2 d, respectively, until the conversion of starting material **34** was complete. The solvent was evaporated under reduced pressure, and the resultant crude product was purified by threefold column chromatography (3:1, petroleum ether-EtOAc and 9:1, petroleum ether-EtOAc and CH_2_Cl_2_→95:5, CH_2_Cl_2_-EtOAc, respectively) to give **38** as a colorless foam (133 mg, 51%). ^1^H NMR (500 MHz, CDCl_3_): δ [ppm] = 0.07 (s, 6H, Si(CH_3_)_2_C(CH_3_)_3_), 0.12 (s, 6H, Si(CH_3_)_2_C(CH_3_)_3_), 0.89 (s, 9H, Si(CH_3_)_2_C(CH_3_)_3_), 0.91 (s, 9H, Si(CH_3_)_2_C(CH_3_)_3_), 1.46 (s, 9H, COOC(CH_3_)_3_), 2.02 (ddd, *J* = 14.2, 8.2, 3.8 Hz, 1H, 5′-H_a_), 2.19 (ddd, *J* = 14.2, 8.2, 2.2 Hz, 1H, 5′-H_b_), 3.63 (dd, *J* = 6.0, 4.1 Hz, 1H, 3′-H), 4.14 (dd, *J* = 4.1, 3.2 Hz, 1H, 2′-H), 4.25 (ddd, *J* = 10.8, 6.3, 1.9 Hz, 1H, 4′-H), 4.45 (ddd, *J* = 7.6, 7.6, 3.8 Hz, 1H, 6′-H), 5.12 (s, 2H, 1″-H), 5.61 (d, *J* = 6.9 Hz, 1H, 6′-NH), 5.62 (d, ^3^*J* = 1.9 Hz, 1H, 1′-H), 7.31–7.38 (m, 5H, aryl-H), 7.84 (d, *J*_HF_ = 6.0 Hz, 1H, 6-H), 8.95 (brs, 1H, 3-NH). ^13^C NMR (126 MHz, CDCl_3_): δ [ppm] = −4.85 (Si(CH_3_)_2_C(CH_3_)_3_), −4.33 (Si(CH_3_)_2_C(CH_3_)_3_), 17.96 (Si(CH_3_)_2_C(CH_3_)_3_), 17.99 (Si(CH_3_)_2_C(CH_3_)_3_), 25.75 (Si(CH_3_)_2_C(CH_3_)_3_), 25.79 (Si(CH_3_)_2_C(CH_3_)_3_), 27.92 (COOC(CH_3_)_3_), 36.35 (C-5′), 51.86 (C-6′), 66.97 (C-1″), 75.15 (C-2′), 75.24 (C-3′), 80.24 (C-4′), 82.98 (COOC(CH_3_)_3_), 91.20 (C-1′), 124.72 (d, *J*_CF_ = 30.0 Hz, C-6), 128.22 (C-3″/C-4″), 128.26 (C-3″/C-4″), 128.51 (C-2″), 139.51 (C-5″), 140.65 (d, *J*_CF_ = 238.3 Hz, C-5), 148.39 (C-2), 155.49 (d, *J*_CF_ = 32.9 Hz, C-4). ^19^F NMR (376 MHz, CDCl_3_): δ [ppm] = −164.51. MS (ESI^+^): *m/z* = 738.5 [M + H]^+^, 760.4 [M + Na]^+^. HRMS (ESI^+^): calcd.: 738.3612 [M + H]^+^, 760.3431 [M + Na]^+^, found: 738.3594, 760.3413. TLC: *R*_f_ = 0.53 (1:1, petroleum ether-EtOAc).

(6′*S*)-6′-*N*-Cbz-2′-deoxyuridinyl amino acid *tert*-butyl ester (**39**): Under strict exclusion of oxygen, didehydro amino acid **35** (600 mg, 1.02 mmol) was dissolved in MeOH (35 mL). After addition of (*S*,*S*)-Me-DUPHOS-Rh **37** (~15 mg, 21 μmol), the solution was stirred under an H_2_ atmosphere (~1 bar) at rt for 4 d. The reaction was monitored by ^1^H NMR spectroscopy. Additional catalyst **37** (~4 mg, 6 μmol) was added and stirring was continued for 2 d until the conversion of starting material **35** was complete. The solvent was evaporated under reduced pressure, and the resultant crude product was purified by column chromatography (3:2, petroleum ether-EtOAc) to give **39** as a colorless foam (562 mg, 93%). ^1^H NMR (500 MHz, CDCl_3_): δ [ppm] = 0.05 (s, 6H, Si(CH_3_)_2_C(CH_3_)_3_), 0.89 (s, 9H, Si(CH_3_)_2_C(CH_3_)_3_), 1.43 (s, 9H, COOC(CH_3_)_3_), 1.93–2.02 (m, 1H, 5′-H_a_), 2.03–2.11 (m, 1H, 2′-H_a_), 2.20 (ddd, *J* = 14.2, 6.7, 2.9 Hz, 1H, 5′-H_b_), 2.23–2.31 (m, 1H, 2′-H_b_), 3.90–3.97 (m, 1H, 4′-H), 4.05 (dd, *J* = 10.8, 4.9 Hz, 1H, 3′-H), 4.41 (dd, *J* = 11.6, 6.7 Hz, 1H, 6′-H), 5.10 (s, 2H, 1″-H), 5.55 (d, *J* = 6.8 Hz, 1H, 6′-NH), 5.77 (d, *J* = 8.0 Hz, 1H, 5-H), 6.14 (dd, *J* = 6.1, 6.1 Hz, 1H, 1′-H), 7.27–7.39 (m, 5H, aryl-H), 7.54 (d, *J* = 8.0 Hz, 1H, 6-H), 8.92 (s, 1H, 3-NH). ^13^C NMR (126 MHz, CDCl_3_): δ [ppm] = −4.74 (Si(CH_3_)_2_C(CH_3_)_3_), −4.48 (Si(CH_3_)_2_C(CH_3_)_3_), 18.03 (Si(CH_3_)_2_C(CH_3_)_3_), 25.79 (Si(CH_3_)_2_C(CH_3_)_3_), 28.06 (COOC(CH_3_)_3_), 36.14 (C-5′), 40.64 (C-2′), 52.23 (C-6′), 67.09 (C-1″), 74.92 (C-3′), 82.92 (COOC(CH_3_)_3_), 83.31 (C-4′), 85.63 (C-1′), 102.68 (C-5), 128.30, 128.37, 128.67 (C-3″, C-4″, C-5″, C-6″, C-7″), 136.38 (C-2″), 140.35 (C-6), 150.15 (C-2), 155.69 (Cbz-C=O), 163.29 (C-4), 170.61 (C-7′). HRMS (CI): calcd.: 590.2892 [M + H]^+^, found: 590.2885. TLC: *R*_f_ = 0.25 (1:1, petroleum ether-EtOAc).

(6′*S*)-6′-*N*-Cbz-thymidinyl amino acid *tert*-butyl ester (**40**): Under strict exclusion of oxygen, didehydro amino acid **36** (600 mg, 0.997 mmol) was dissolved in MeOH (35 mL). After addition of (*S*,*S*)-Me-DUPHOS-Rh **37** (~20 mg, 28 μmol), the solution was stirred under an H_2_ atmosphere (~1 bar) at rt for 7 d. The reaction was monitored by ^1^H NMR spectroscopy. After complete conversion of starting material **36**, the solvent was evaporated under reduced pressure. The resultant crude product was purified by column chromatography (1:1, petroleum ether-EtOAc) to give **40** as a colorless foam (566 mg, 95%). ^1^H NMR (500 MHz, CDCl_3_): δ [ppm] = 0.06 (s, 6H, Si(CH_3_)_2_C(CH_3_)_3_), 0.88 (s, 9H, Si(CH_3_)_2_C(CH_3_)_3_), 1.43 (s, 9H, COOC(CH_3_)_3_), 1.97 (s, 3H, 7-H), 1.99–2.09 (m, 2H, 2′-H_a_, 5′-H_a_), 2.14–2.20 (m, 1H, 5′-H_b_), 2.22 (ddd, *J* = 13.5, 6.5, 4.3 Hz, 1H, 2′-H_b_), 3.92–3.97 (m, 1H, 4′-H), 4.07 (ddd, *J* = 6.5, 4.3, 4.3 Hz, 1H, 3′-H), 4.42 (dd, *J* = 11.8, 7.1 Hz, 1H, 6′-H), 5.10 (s, 2H, 1″-H), 5.54 (d, *J* = 6.7 Hz, 1H, 6′-NH), 6.18 (dd, *J* = 6.5, 6.5 Hz, 1H, 1′-H), 7.29–7.36 (m, 5H, aryl-H), 7.37 (s, 1H, 6-H), 8.53 (brs, 1H, 3-NH). ^13^C NMR (126 MHz, CDCl_3_): δ [ppm] = −4.71 (Si(CH_3_)_2_C(CH_3_)_3_), −4.47 (Si(CH_3_)_2_C(CH_3_)_3_), 12.46 (C-7), 18.05 (Si(CH_3_)_2_C(CH_3_)_3_), 25.18 (Si(CH_3_)_2_C(CH_3_)_3_), 28.06 (COOC(CH_3_)_3_), 36.26 (C-5′), 40.39 (C-2′), 52.16 (C-6′), 67.06 (C-1″), 75.11 (C-3′), 82.89 (COOC(CH_3_)_3_), 83.26 (C-4′), 85.27 (C-1′), 111.33 (C-5), 128.26, 128.37, 128.68 (C-3″, C-4″, C-5″, C-6″, C-7″), 136.00 (C-6), 136.41 (C-2″), 150.18 (C-2), 155.65 (Cbz-C=O), 163.74 (C-4), 170.65 (C-7′). HRMS (CI): calcd.: 604.3049 [M + H]^+^, found: 604.3029. TLC: *R*_f_ = 0.30 (1:1, petroleum ether-EtOAc).

(6′*S*)-5,6-dihydrouridinyl amino acid *tert*-butyl ester (**42**): (6′*S*)-6′-*N*-Cbz-uridinyl amino acid *tert*-butyl ester **41** [31] (100 mg, 0.139 mmol) was dissolved in *i*-PrOH (20 mL) and stirred over molecular sieve (3 Å) at rt for 15 min. Pd black (130 mg, 1.22 mmol) was added and the reaction mixture was stirred under an H_2_ atmosphere (~1 bar) at rt for 4 h. After filtration through a syringe filter and washing the filter with *i*-PrOH (50 °C, 45 mL), the solvent of the combined filtrates was evaporated under reduced pressure to give **42** as a colorless foam (82 mg, 96%). ^1^H NMR (300 MHz, CDCl_3_): δ [ppm] = 0.06 (s, 3H, Si(CH_3_)_2_C(CH_3_)_3_), 0.07 (s, 3H, Si(CH_3_)_2_C(CH_3_)_3_), 0.08 (s, 3H, Si(CH_3_)_2_C(CH_3_)_3_), 0.09 (s, 3H, Si(CH_3_)_2_C(CH_3_)_3_), 0.90 (s, 9H, Si(CH_3_)_2_C(CH_3_)_3_), 0.91 (s, 9H, Si(CH_3_)_2_C(CH_3_)_3_), 1.46 (s, 9H, COOC(CH_3_)_3_), 1.68 (ddd, *J* = 13.9, 9.4, 7.1 Hz, 1H, 5′-H_a_), 2.12 (ddd, *J* = 13.9, 6.0, 2.6 Hz, 1H, 5′-H_b_), 2.63-–2.69 (m, 2H, 5-H), 3.29–3.40 (m, 1H, 6-H_a_), 3.49 (dd, *J* = 6.3, 6.3 Hz, 1H, 6-H_b_), 3.56 (dd, *J* = 7.1, 7.0 Hz, 1H, 6′-H), 3.74 (dd, *J* = 5.7, 4.6 Hz, 1H, 3′-H), 4.01 (ddd, *J* = 9.4, 5.7, 2.6 Hz, 1H, 4′-H), 4.14 (dd, *J* = 4.6, 4.0 Hz, 1H, 2′-H), 5.59 (d, *J* = 4.0 Hz, 1H, 1′-H), 7.61 (brs, 1H, 3-NH). ^13^C NMR (126 MHz, CDCl_3_): δ [ppm] = −4.49 (Si(CH_3_)_2_C(CH_3_)_3_), −4.40 (Si(CH_3_)_2_C(CH_3_)_3_), −4.38 (Si(CH_3_)_2_C(CH_3_)_3_), −4.00 (Si(CH_3_)_2_C(CH_3_)_3_), 18.15 (Si(CH_3_)_2_C(CH_3_)_3_), 18.24 (Si(CH_3_)_2_C(CH_3_)_3_), 25.93 (Si(CH_3_)_2_C(CH_3_)_3_), 26.02 (Si(CH_3_)_2_C(CH_3_)_3_), 28.16 (COOC(CH_3_)_3_), 31.29 (C-5), 38.47 (C-5′), 38.70 (C-6), 53.62 (C-6′), 73.52 (C-2′), 76.14 (C-3′), 80.81 (C-4′), 81.69 (COOC(CH_3_)_3_), 91.64 (C-1′), 152.17 (C-2), 169.44 (C-4), 173.97 (C-7′). HRMS (CI): calcd.: 588.3495 [M + H]^+^, found: 588.3488.

*N*-Cbz-protected 5,6-dihydrouridine derivative (**44**): Nucleosyl amino acid ester **42** (100 mg, 0.170 mmol) was dissolved in THF (14 mL) and aldehyde **43** [24,34] (70 mg, 0.22 mmol) was added. The reaction mixture was stirred over molecular sieve (3 Å) at rt for 23 h. NaBH(OAc)_3_ (80 mg, 0.39 mmol) and Amberlyst 15^TM^ (14 mg, 44 μmol) were added and stirring was continued at rt for 22 h. The reaction mixture was filtered and the residue was washed with EtOAc (150 mL). The combined filtrates were washed with sat. NaHCO_3_ solution (100 mL) and brine (50 mL). The aqueous layer was extracted with EtOAc (3 × 100 mL). The combined organics were dried over Na_2_SO_4_ and the solvent was evaporated under reduced pressure. The resultant crude product was purified by column chromatography (98:2, CH_2_Cl_2_-MeOH) to give **44** as a colorless solid (93 mg, 58%). ^1^H NMR (500 MHz, CD_3_OD): δ [ppm] = 0.11 (s, 3H, Si(CH_3_)_2_C(CH_3_)_3_), 0.12 (s, 3H, Si(CH_3_)_2_C(CH_3_)_3_), 0.13 (s, 3H, Si(CH_3_)_2_C(CH_3_)_3_), 0.14 (s, 3H, Si(CH_3_)_2_C(CH_3_)_3_), 0.84–0.91 (m, 6H, Leu-5-H), 0.92 (s, 9H, Si(CH_3_)_2_C(CH_3_)_3_), 0.93 (s, 9H, Si(CH_3_)_2_C(CH_3_)_3_), 1.49 (s, 9H, COOC(CH_3_)_3_), 1.53 (dd, *J* = 7.2, 7.2 Hz, 2H, Leu-3-H), 1.63–1.72 (m, 3H, 2″-H, Leu-4-H), 1.78 (ddd, *J* = 13.4, 11.2, 4.9 Hz, 1H, 5′-H_a_), 1.99 (ddd, *J* = 13.4, 9.6, 2.7 Hz, 1H, 5′-H_b_), 2.51–2.58 (m, 1H, 1″-H_a_), 2.60–2.67 (m, 3H, 1″-H_b_, 5-H), 3.18–3.36 (m, 2H, 6′-H, 3″-H_a_), 3.39 (ddd, *J* = 12.9, 6.6, 6.6 Hz, 1H, 6-H_a_), 3.47 (ddd, *J* = 12.9, 6.7, 6.7 Hz, 1H, 6-H_b_), 3.56 (ddd, *J* = 8.1, 4.4, 4.4 Hz, 1H, 3″-H_b_), 3.85 (dd, *J* = 4.8, 4.8 Hz, 1H, 3′-H), 3.88–3.94 (m, 1H, 4′-H), 4.04–4.14 (m, 1H, Leu-2-H), 4.25 (dd, *J* = 4.8, 4.6 Hz, 1H, 2′-H), 5.09 (s, 2H, 1″′-H), 5.77 (d, *J* = 4.6 Hz, 1H, 1′-H), 7.25–7.39 (m, 5H, aryl-H). ^13^C NMR (126 MHz, CD_3_OD): δ [ppm] = −4.36 (Si(CH_3_)_2_C(CH_3_)_3_), −4.34 (Si(CH_3_)_2_C(CH_3_)_3_), −4.13 (Si(CH_3_)_2_C(CH_3_)_3_), −3.93 (Si(CH_3_)_2_C(CH_3_)_3_), 18.90 (Si(CH_3_)_2_C(CH_3_)_3_), 18.94 (Si(CH_3_)_2_C(CH_3_)_3_), 23.37 (Leu-C-5), 23.46 (Leu-C-5), 25.99 (Leu-C-4), 26.41 (Si(CH_3_)_2_C(CH_3_)_3_), 26.48 (Si(CH_3_)_2_C(CH_3_)_3_), 28.44 (COOC(CH_3_)_3_), 29.97 (C-2″), 31.93 (C-5), 37.46 (C-5′), 38.09 (C-6), 39.00 (C-3″), 42.22 (Leu-C-3), 46.02 (C-1″), 55.23 (Leu-C-2), 60.41 (C-6′), 67.71 (C-1″′), 74.14 (C-2′), 77.15 (C-3′), 81.10 (C-4′), 83.24 (COOC(CH_3_)_3_), 91.77 (C-1′), 128.85 (C-3″′, C-7″′), 129.09 (C-5″′), 129.50 (C-4″′, C-6″′), 138.20 (C-2″′), 154.93 (C-2), 158.39 (Cbz-C=O), 172.49 (C-4), 175.67 (C-7′), 181.44 (Leu-C-1). HRMS (ESI^+^): calcd.: 892.5282 [M + H]^+^, found: 892.5261.

5,6-Dihydrouridine derivative (**45**): *N*-Cbz-protected 5,6-dihydrouridine derivative **44** (51 mg, 57 μmol) was dissolved in MeOH (10 mL) and Pd/C (10%, 3.6 mg, 3.4 μmol Pd) was added. The reaction mixture was stirred under an H_2_ atmosphere (~1 bar) at rt for 2 h. After filtration through a syringe filter and washing the filter with EtOAc (50 °C, 20 mL), the solvent of the combined filtrates was evaporated under reduced pressure to give **45** as a colorless solid (42 mg, quant.). ^1^H NMR (500 MHz, CD_3_OD): δ [ppm] = 0.11 (s, 3H, Si(CH_3_)_2_C(CH_3_)_3_), 0.12 (s, 3H, Si(CH_3_)_2_C(CH_3_)_3_), 0.13 (s, 3H, Si(CH_3_)_2_C(CH_3_)_3_), 0.14 (s, 3H, Si(CH_3_)_2_C(CH_3_)_3_), 0.89–0.96 (m, 6H, Leu-5-H), 0.92 (s, 9H, Si(CH_3_)_2_C(CH_3_)_3_), 0.94 (s, 9H, Si(CH_3_)_2_C(CH_3_)_3_), 1.41–1.55 (m, 2H, Leu-3-H), 1.49 (s, 9H, COOC(CH_3_)_3_), 1.64–1.72 (m, 3H, 2″-H, Leu-4-H), 1.76 (ddd, *J* = 13.9, 10.8, 4.9 Hz, 1H, 5′-H_a_), 1.96–2.02 (m, 1H, 5′-H_b_), 2.52 (ddd, *J* = 11.6, 7.0, 7.0 Hz, 1H, 1″-H_a_), 2.59–2.68 (m, 3H, 5-H, 1″-H_b_), 3.19–3.30 (m, 2H, 6′-H, 3″-H_a_), 3.41 (ddd, *J* = 13.6, 6.8, 6.8 Hz, 1H, 6-H_a_), 3.49 (ddd, *J* = 13.6, 6.8, 6.8 Hz, 1H, 6-H_b_), 3.85 (dd, *J* = 4.8, 4.8 Hz, 1H, 3′-H), 3.88–3.93 (m, 1H, 4′-H), 4.04–4.14 (m, 1H, Leu-2-H), 4.24 (dd, *J* = 4.8, 4.5 Hz, 1H, 2′-H), 5.76 (d, *J* = 4.5 Hz, 1H, 1′-H). ^13^C NMR (126 MHz, CD_3_OD): δ [ppm] = −4.36 (Si(CH_3_)_2_C(CH_3_)_3_), −4.35 (Si(CH_3_)_2_C(CH_3_)_3_), −4.13 (Si(CH_3_)_2_C(CH_3_)_3_), −3.92 (Si(CH_3_)_2_C(CH_3_)_3_), 18.90 (Si(CH_3_)_2_C(CH_3_)_3_), 18.93 (Si(CH_3_)_2_C(CH_3_)_3_), 22.58 (Leu-C-5), 23.41 (Leu-C-5), 25.92 (Leu-C-4), 26.41 (Si(CH_3_)_2_C(CH_3_)_3_), 26.48 (Si(CH_3_)_2_C(CH_3_)_3_), 28.46 (COOC(CH_3_)_3_), 30.43 (C-2″), 31.95 (C-5), 38.17 (C-5′), 38.25 (C-6), 38.85 (C-3″), 45.71 (Leu-C-3), 46.16 (C-1″), 54.68 (Leu-C-2), 60.94 (C-6′), 74.24 (C-2′), 77.16 (C-3′), 81.13 (C-4′), 82.77 (COOC(CH_3_)_3_), 91.61 (C-1′), 154.93 (C-2), 172.49 (C-4), 174.97 (C-7′), 178.16 (Leu-C-1). MS (ESI^+^): calcd.: 758.49 [M + H]^+^, found: 758.57.

### 4.2. Overexpression of MraY from S. Aureus

The overexpression of MraY was performed as described before [39,41,49].

### 4.3. Fluorescence Based MraY Assay

The assay was performed as described before [39,41,49]. Measured data and inhibition curves are shown in the Supplementary Materials Figures S1–S4.

## 5. Conclusions

In summary, we report valuable new SAR insights for synthetic analogues of muraymycin antibiotics with structural variations in the nucleoside core unit. For this purpose, we have synthesized four novel muramycin analogues **13**–**16**. The modular preparation of **13**–**15** demonstrated the efficiency of a recently established bipartite approach employing a solid phase-supported protocol for the construction of the peptide moiety [42]. Furthermore, it was shown that our previously reported route towards 5’-defunctionalized nucleosyl amino acids [31] is also applicable for structurally modified nucleosides.

By in vitro evaluation of **13**–**16** as inhibitors of the bacterial target protein MraY, it was found that the introduction of substituents in the 5-position of the pyrimidine nucleobase led to a drastic loss of inhibitory potency towards MraY. In contrast, the removal of nucleobase aromaticity (by reduction of the uracil C5-C6 double bond) only resulted in a ca. tenfold decrease in activity. The formal removal of the 2′-hydroxy group, however, led to a retention of inhibitory activity, thus demonstrating that modifications of the ribose moiety might be well-tolerated. These results were in good agreement with previous X-ray crystallographic studies on the interaction of naturally occurring muraymycin D2 **7** with MraY. This indicates that such X-ray crystal structures of MraY-inhibitor complexes can be highly useful for the structure-based design of new MraY inhibitors. In this work, only four nucleoside-modified muraymycin analogues were studied, thus limiting the possibility to derive robust general instructions for the future design of muraymycin analogues from the reported data. Nevertheless, the obtained SAR insights strongly suggest that nucleobase modifications should be avoided, while the ribose moiety appears to be a good choice for the introduction of novel structural variations. This may even include lipophilic moieties in order to improve the bacterial cellular uptake of otherwise rather polar muraymycin analogues, thus providing congeners with improved activities in cellulo. Such development of synthetic muraymycin analogues towards potential antibacterial drug candidates is currently on the way in our laboratories.

## Figures and Tables

**Table 1:**
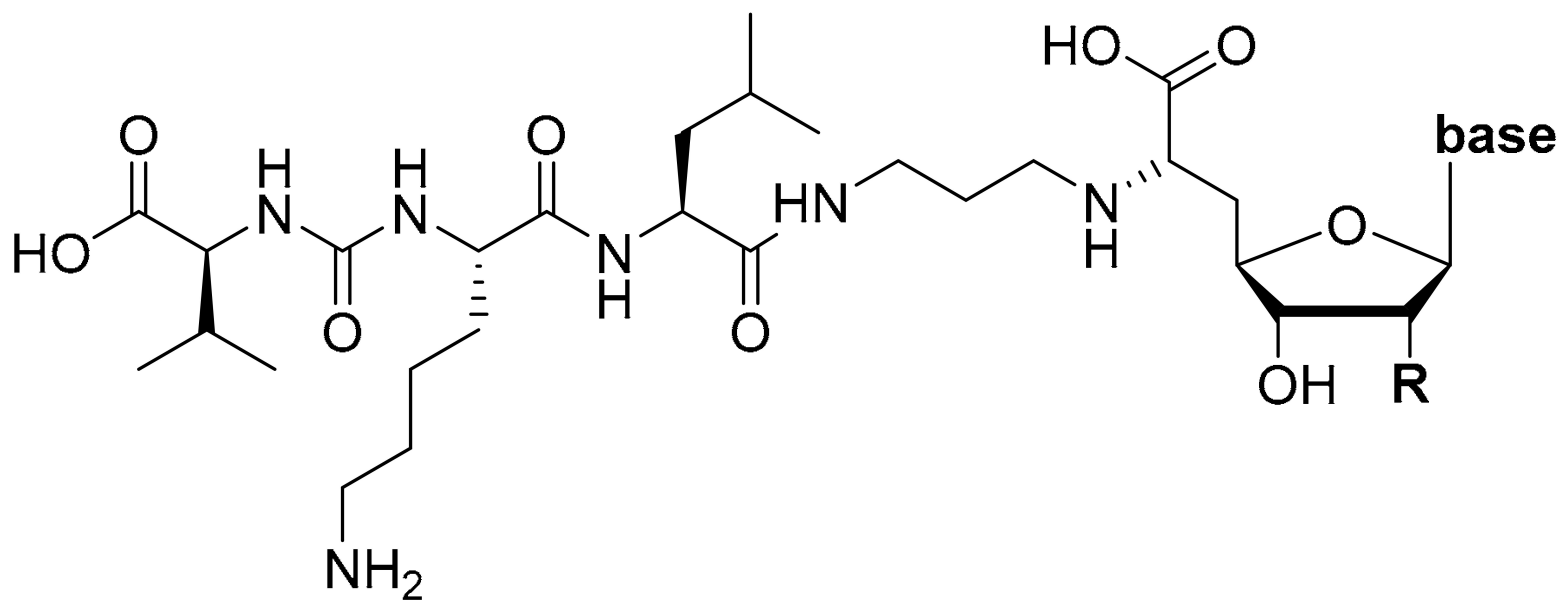
In vitro inhibitory activities of muraymycin analogues **13**–**16** against MraY from *S. aureus*.

Compound	R	Base	IC_50_ [µM]^1^
**13**	OH	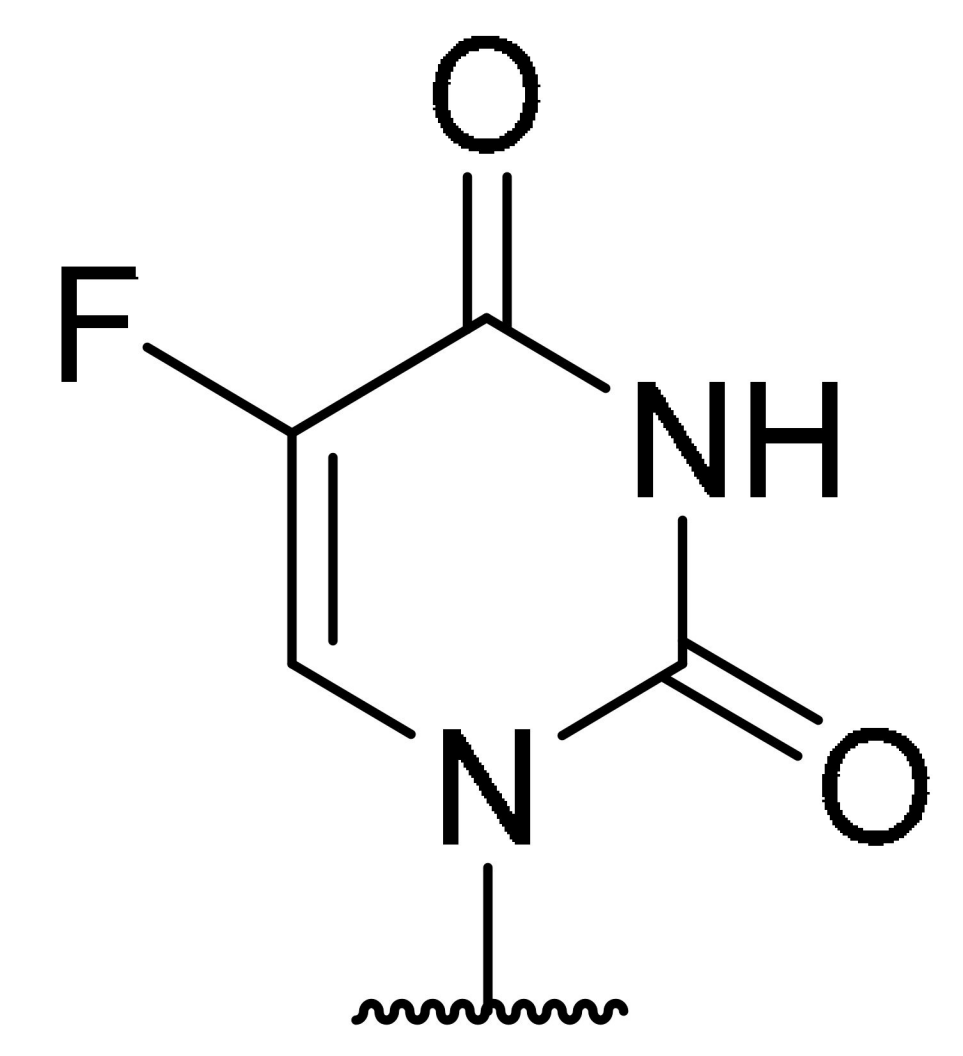	>100 (49% inhibition @1 mM)
**14**	H	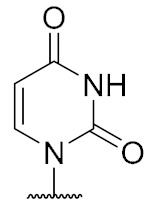	4.6 ± 1.8
**15**	H	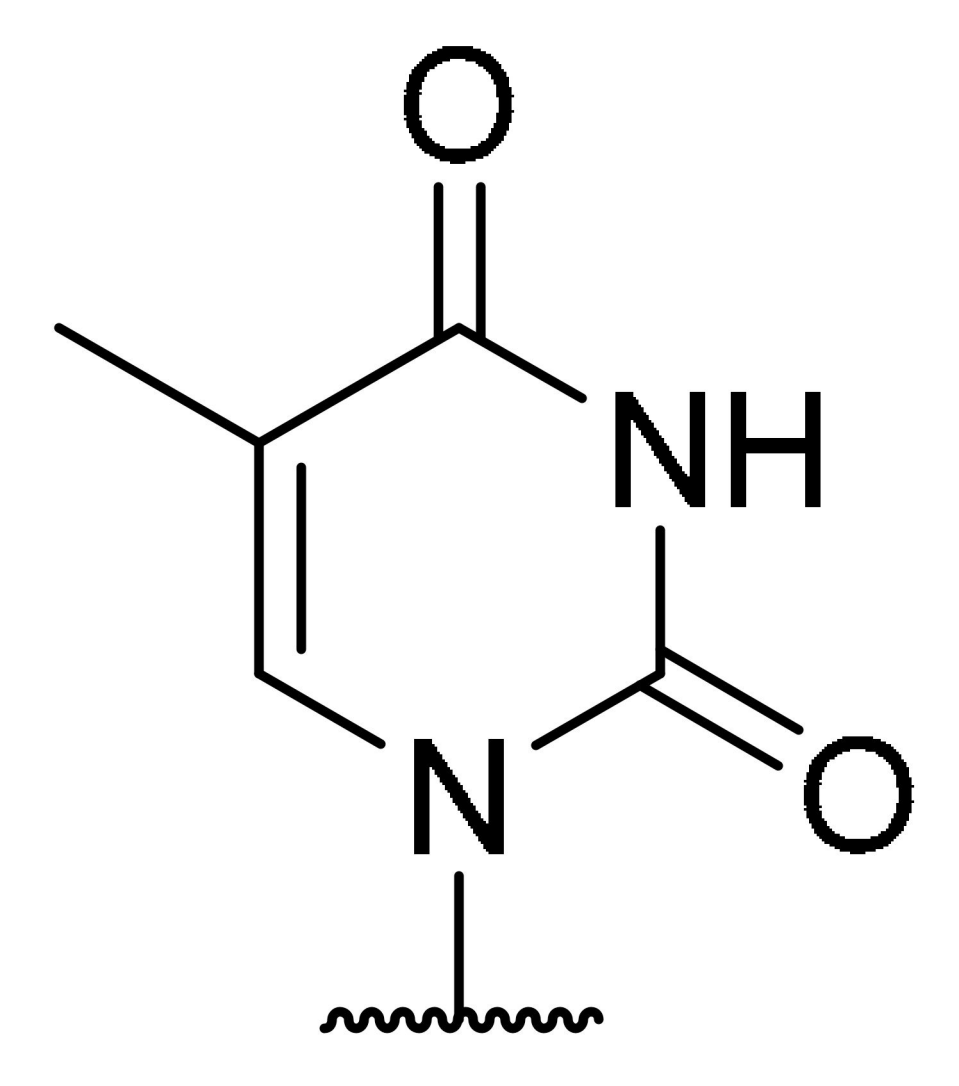	>100 (38% inhibition @1 mM)
**16**	OH	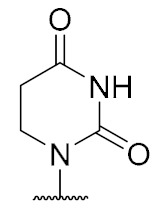	27 ± 8
**8**	OH	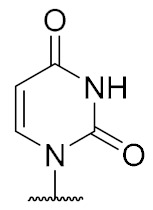	2.5 ± 0.6

^1^ A crude membrane preparation of MraY from *S. aureus*, heterologously overexpressed in *E. coli*, was used. Measurements for significantly active compounds (IC_50_ < 100 μM) were carried out in triplicates, for weakly active compounds (IC_50_ > 100 μM) in duplicates. Data for significantly active compounds are given as mean value ± standard deviation. For weakly active compounds, percentage of inhibition at 1 mM is also listed.

## Supplementary Materials

The following are available online, data from the MraY assays, copies of NMR spectra and Figures S1–S5.

## References

[1] Taubes G. (2008). The bacteria fight back. Science.

[2] Cooper M.A., Shlaes D. (2011). Fix the antibiotics pipeline. Nature.

[3] Walsh C. (2003). Where will new antibiotics come from?. Nat. Rev. Microbiol..

[4] Hamed R.B., Gomez-Castellanos J.R., Henry L., Ducho C., McDonough M.A., Schofield C.J. (2013). The enzymes of β-lactam biosynthesis. Nat. Prod. Rep..

[5] Dini C. (2005). MraY Inhibitors as novel antibacterial agents. Curr. Top. Med. Chem..

[6] Lloyd A.J., Brandish P.E., Gilbey A.M., Bugg T.D.H. (2004). Phospho-N-acetyl-muramyl-pentapeptide translocase from *Escherichia coli*: catalytic role of conserved aspartic acid residues. J. Bacteriol..

[7] Struve W.G., Neuhaus F.C. (1965). Evidence for an initial acceptor of UDP-NAc-muramyl-pentapeptide in the synthesis of bacterial mucopeptide. Biochem. Biophys. Res. Commun..

[8] Anderson J.S., Matsuhashi M., Haskin M.A., Strominger J.L. (1965). Lipid-phosphoacetylmuramyl-pentapeptide and lipid-phosphodisaccharide-pentapeptide: presumed membrane transport intermediates in cell wall synthesis. Proc. Natl. Acad. Sci. USA.

[9] Heydanek M.G., Struve W.G., Neuhaus F.C. (1969). Initial state in peptidoglycan synthesis. III. Kinetics and uncoupling of phospho-N-acetylmuramyl-pentapeptide translocase (uridine 5’-phosphate). Biochemistry.

[10] Ikeda M., Wachi M., Jung H.K., Ishino F., Matsuashi M. (1991). The *Escherichia coli mraY* gene encoding UDP-N-acetylmuramoyl-acetylmuramoyl-pentapeptide transferase. J. Bacteriol..

[11] Boyle D.S., Donachie W.D. (1998). *mraY* is an essential gene for cell growth in *Escherichia coli*. J. Bacteriol..

[12] Vollmer W., Blanot D., De Pedro M.A. (2008). Peptidoglycan structure and architecture. Fems Microbiol. Rev..

[13] Wiegmann D., Koppermann S., Wirth M., Niro G., Leyerer K., Ducho C. (2016). Muraymycin nucleoside-peptide antibiotics: Uridine-derived natural products as lead structures for the development of novel antibacterial agents. Beilstein J. Org. Chem..

[14] Winn M., Goss R.J.M., Kimura K.I., Bugg T.D.H. (2010). Antimicrobial nucleoside antibiotics targeting cell wall assembly: Recent advances in structure-function studies and nucleoside biosynthesis. Nat. Prod. Rep..

[15] Ichikawa S., Yamaguchi M., Matsuda A. (2015). Antibacterial nucleoside natural products inhibiting phospho-MurNAc-pentapeptide translocase; chemistry and structure–activity relationship. Curr. Med. Chem..

[16] Bugg T.D.H., Rodolis M.T., Mihalyi A., Jamshidi S. (2016). Inhibition of phospho-MurNAc-pentapeptide translocase (MraY) by nucleoside natural product antibiotics, bacteriophage ϕX174 lysis protein E, and cationic antibacterial peptides. Bioorg. Med. Chem..

[17] Lin Y., Li Z., Francisco G.D., Mcdonald L.A., Davis R.A., Singh G., Yang Y., Mansour T.S. (2002). Muraymycins, Novel Peptidoglycan Biosynthesis Inhibitors: Semisynthesis and SAR of Their Derivatives. Bioorg. Med. Chem. Lett..

[18] Cui Z., Wang X., Koppermann S., Thorson J.S., Ducho C., Van Lanen S.G. (2018). Antibacterial Muraymycins from Mutant Strains of *Streptomyces* sp. NRRL 30471. J. Nat. Prod..

[19] Carter G.T., Lotvin J.A., McDonald L.A. (2002). (American Cyanamid Company) Antibiotics AA-896.

[20] McDonald L.A., Barbieri L.R., Carter G.T., Lenoy E., Lotvin J., Petersen P.J., Siegel M.M., Singh G., Williamson R.T. (2002). Structures of the muraymycins, novel peptidoglycan biosynthesis inhibitors. J. Am. Chem. Soc..

[21] Tanino T., Al-Dabbagh B., Mengin-Lecreulx D., Bouhss A., Oyama H., Ichikawa S., Matsuda A. (2011). Mechanistic analysis of muraymycin analogues: A guide to the design of MraY inhibitors. J. Med. Chem..

[22] Chung B.C., Mashalidis E.H., Tanino T., Kim M., Matsuda A., Hong J., Ichikawa S., Lee S.Y. (2016). Structural insights into inhibition of lipid I production in bacterial cell wall synthesis. Nature.

[23] Koppermann S., Ducho C. (2016). Natural products at work: structural insights into inhibition of the bacterial membrane protein MraY. Angew. Chem. Int. Ed..

[24] Wiegmann D., Koppermann S., Ducho C. (2018). Aminoribosylated Analogues of Muraymycin Nucleoside Antibiotics. Molecules.

[25] Chung B.C., Zhao J., Gillespie R.A., Kwon D.-Y., Guan Z., Hong J., Zhou P., Lee S.-Y. (2013). Crystal structure of MraY, an essential membrane enzyme for bacterial cell wall synthesis. Science.

[26] Mashalidis E.H., Kaeser B., Terasawa Y., Katsuyama A., Kwon D.-Y., Lee K., Hong J., Ichikawa S., Lee S.-Y. (2019). Chemical logic of MraY inhibition by antibacterial nucleoside natural products. Nat. Commun..

[27] Tanino T., Hirano S., Ichikawa S., Matsuda A. (2008). Synthetic study of muraymycins using Ugi-four component reaction. Nucleic Acids Symp. Ser..

[28] Tanino T., Ichikawa S., Shiro M., Matsuda A. (2010). Total Synthesis of (−)-Muraymycin D2 and Its Epimer. J. Org. Chem..

[29] Mitachi K., Aleiwi B.A., Schneider C.M., Siricilla S., Kurosu M. (2016). Stereocontrolled Total Synthesis of Muraymycin D1 Having a Dual Mode of Action against *Mycobacterium tuberculosis*. J. Am. Chem. Soc..

[30] Ries O., Büschleb M., Granitzka M., Stalke D., Ducho C. (2014). Amino acid motifs in natural products: Synthesis of *O*-acylated derivatives of (2*S*,3*S*)-3-hydroxyleucine. Beilstein J. Org. Chem..

[31] Spork A.P., Wiegmann D., Granitzka M., Stalke D., Ducho C. (2011). Stereoselective synthesis of uridine-derived nucleosyl amino acids. J. Org. Chem..

[32] Büschleb M., Granitzka M., Stalke D., Ducho C. (2012). A biomimetic domino reaction for the concise synthesis of capreomycidine and epicapreomycidine. Amino Acids.

[33] Ries O., Ochmann A., Ducho C. (2011). Synthesis of *N*-alkyl-*N*-hydroxyguanidines: A comparative study using different protecting group strategies. Synthesis.

[34] Yamashita A., Norton E., Petersen P.J., Rasmussen B.A., Singh G., Yang Y., Mansour T.S., Ho D.M. (2003). Muraymycins, novel peptidoglycan biosynthesis inhibitors: Synthesis and SAR of their analogues. Bioorg. Med. Chem. Lett..

[35] Aleiwi B.A., Schneider C.M., Kurosu M. (2012). Synthesis of Ureidomuraymycidine Derivatives for Structure– Activity Relationship Studies of Muraymycins. J. Org. Chem..

[36] Spork A.P., Koppermann S., Dittrich B., Herbst-Irmer R., Ducho C. (2010). Efficient synthesis of the core structure of muraymycin and caprazamycin nucleoside antibiotics based on a stereochemically revised sulfur ylide reaction. Tetrahedron Asymmetry.

[37] Spork A.P., Ducho C. (2013). Stereocontrolled synthesis of 5′- and 6′-epimeric analogues of muraymycin nucleoside antibiotics. Synlett.

[38] Spork A.P., Büschleb M., Ries O., Wiegmann D., Boettcher S., Mihalyi A., Bugg T.D.H., Ducho C. (2014). Lead Structures for New Antibacterials: Stereocontrolled Synthesis of a Bioactive Muraymycin Analogue. Chem. Eur. J..

[39] Koppermann S., Cui Z., Fischer P.D., Wang X., Ludwig J., Thorson J.S., Van Lanen S.G., Ducho C. (2018). Insights into the Target Interaction of Naturally Occurring Muraymycin Nucleoside Antibiotics. ChemMedChem.

[40] Tanino T., Ichikawa S., Al-Dabbagh B., Bouhss A., Oyama H., Matsuda A. (2010). Synthesis and biological evaluation of muraymycin analogues active against anti-drug-resistant bacteria. ACS Med. Chem. Lett..

[41] Spork A.P., Koppermann S., Schier S., Linder R., Ducho C. (2018). Analogues of muraymycin nucleoside antibiotics with epimeric uridine-derived core structures. Molecules.

[42] Leyerer K., Koppermann S., Ducho C. (2019). Solid Phase-Supported Synthesis of Muraymycin Analogues. Eur. J. Org. Chem..

[43] Zhu X.-F., Williams H.J., Scott A.I. (2000). Facile and highly selective 5′-desilylation of multisilylated nucleosides. J. Chem. Soc. Perkin Trans. 1.

[44] Khan A.T., Mondal E. (2003). A highly efficient and useful synthetic protocol for the cleavage of *tert*-Butyldimethylsilyl (TBS) ethers using a catalytic amount of acetyl chloride in dry methanol. Synlett.

[45] Burk M.J. (1991). C2-symmetric bis(phospholanes) and their use in highly enantioselective hydrogenation reactions. J. Am. Chem. Soc..

[46] Masquelin T., Broger E., Müller K., Schmid R., Obrecht D. (1994). Synthesis of enantiomerically pure D- and L-(heteroaryl)alanines by asymmetric hydrogenation of (*Z*)-α-amino-αβ-didehydro esters. Helv. Chim. Acta.

[47] Ducho C., Hamed R.B., Batchelar E.T., Sorensen J.L., Odell B., Schofield C.J. (2009). Synthesis of regio- and stereoselectively deuterium-labelled derivatives of L-glutamate semialdehyde for studies on carbapenem biosynthesis. Org. Biomol. Chem..

[48] Brandish P.E., Burnham M.K., Lonsdale J.T., Southgate R., Inukai M., Bugg T.D.H. (1996). Slow binding inhibition of phospho-N-acetylmuramyl-pentapeptide-translocase (*Escherichia coli*) by mureidomycin A. J. Biol. Chem..

[49] Wohnig S., Spork A.P., Koppermann S., Mieskes G., Gisch N., Jahn R., Ducho C. (2016). Total Synthesis of Dansylated Park’s Nucleotide for High-Throughput MraY Assays. Chem. Eur. J..

[50] Brandish P.E., Kimura K.I., Inukai M., Southgate R., Lonsdale J.T., Bugg T.D.H. (1996). Modes of action of tunicamycin, liposidomycin B, and mureidomycin A: Inhibition of phospho-N-acetylmuramyl-pentapeptide translocase from *Escherichia coli*. Antimicrob. Agents Chemother..

[51] Stachyra T., Dini C., Ferrari P., Bouhss A., van Heijenoort J., Mengin-Lecreulx D., Blanot D., Biton J., Le Beller D. (2004). Fluorescence detection-based functional assay for high-throughput screening for MraY. Antimicrob. Agents Chemother..

[52] Bouhss A., Crouvoisier M., Blanot D., Mengin-Lecreulx D. (2004). Purification and characterization of the bacterial MraY translocase catalyzing the first membrane step of peptidoglycan biosynthesis. J. Biol. Chem..

