# Supplementary material for: Muraymycin Nucleoside Antibiotics: Structure-Activity Relationship for Variations in the Nucleoside Unit
Source: Molecules. 2019 Dec 19;25(1):22. doi: 10.3390/molecules25010022 (PMC6983020; doi:10.3390/molecules25010022)
Supplement: Supplementary file 1 [file molecules-25-00022-s001.pdf]

Supplementary Materials

Muraymycin Nucleoside Antibiotics: Structure-Activity Relationship for Variations in the Nucleoside Unit

Anna Heib[1], Giuliana Niro[1], Stefanie C. Weck[1], Stefan Koppermann[1] and Christian Ducho[1,*]

[1] Saarland University, Department of Pharmacy, Pharmaceutical and Medicinal Chemistry, Campus C2 3, 66 123 Saarbrücken, Germany

*Corresponding author *E-mail: christian.ducho@uni-saarland.de*

Table of contents

Measured data and inhibition curves from MraY assays……………...……………… S2

^{1}H, ^{13}C and ^{19}F NMR spectra of synthesized compounds...........….......................... S6

Measured data and inhibition curves from MraY assays

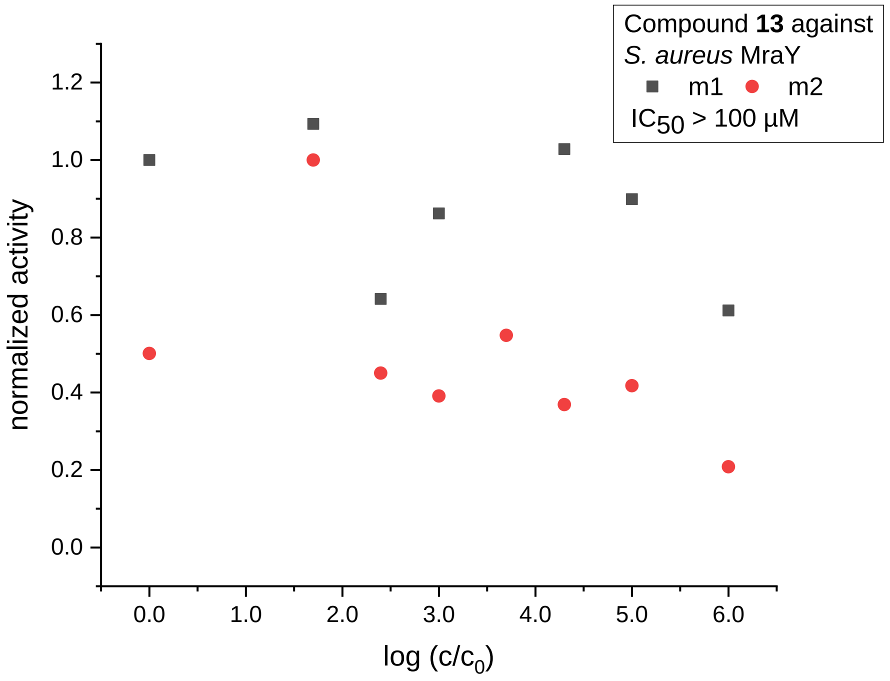

Figure S1. MraY assay with MraY from *S. aureus* (crude membranes) and compound **13** as inhibitor (original data, shown as individual series of measurements; c_0 = 1 nM).

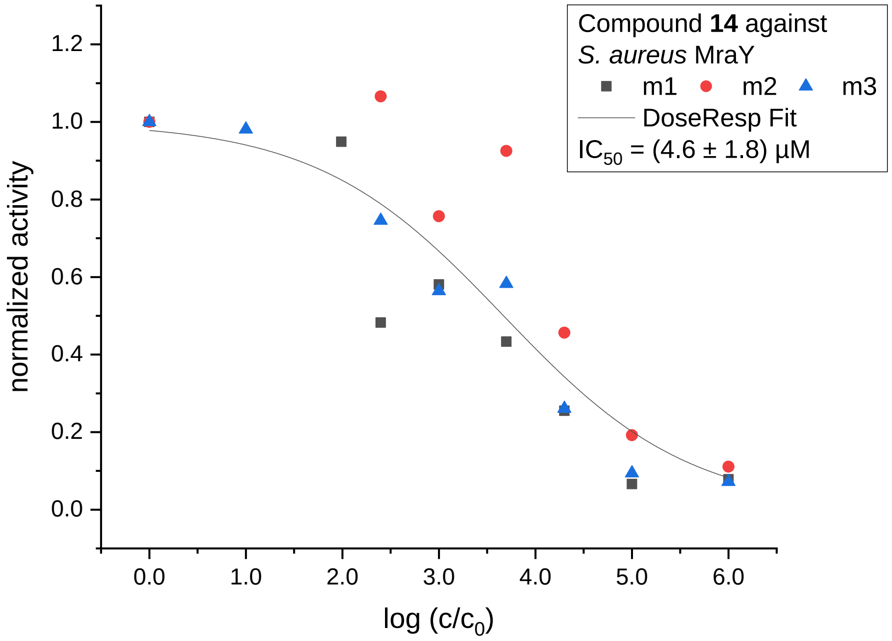

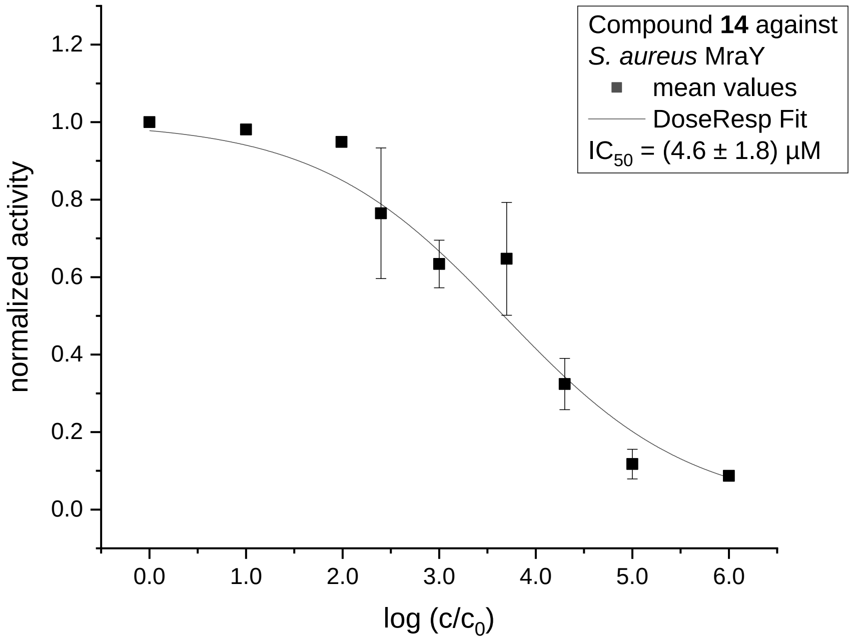

Figure S2. MraY assay with MraY from *S. aureus* (crude membranes) and compound **14** as inhibitor, including the fitted curve for MraY activity (top: original data used for fitting the curve, with data points shown as individual series of measurements; bottom: values displayed as mean values and standard deviations, for illustrative purposes; c_0 = 1 nM).

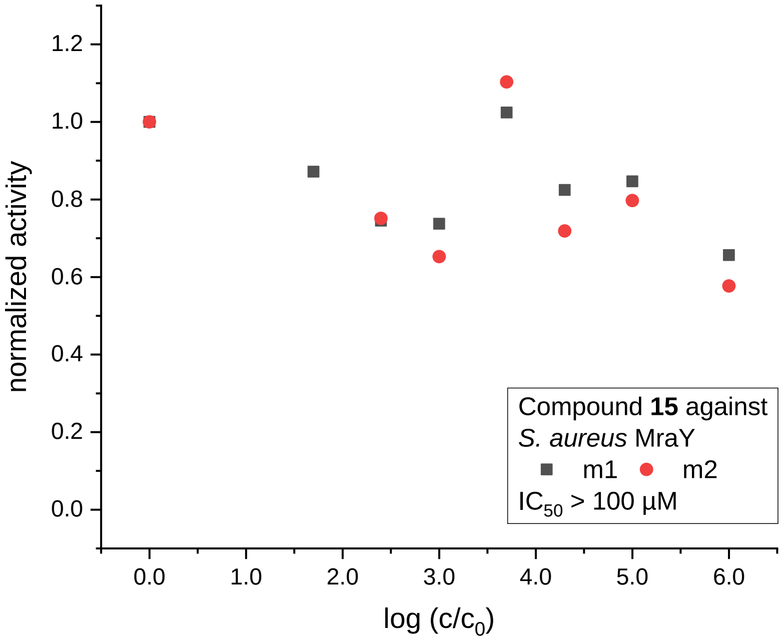

Figure S3. MraY assay with MraY from *S. aureus* (crude membranes) and compound **15** as inhibitor (original data, shown as individual series of measurements; c_0 = 1 nM).

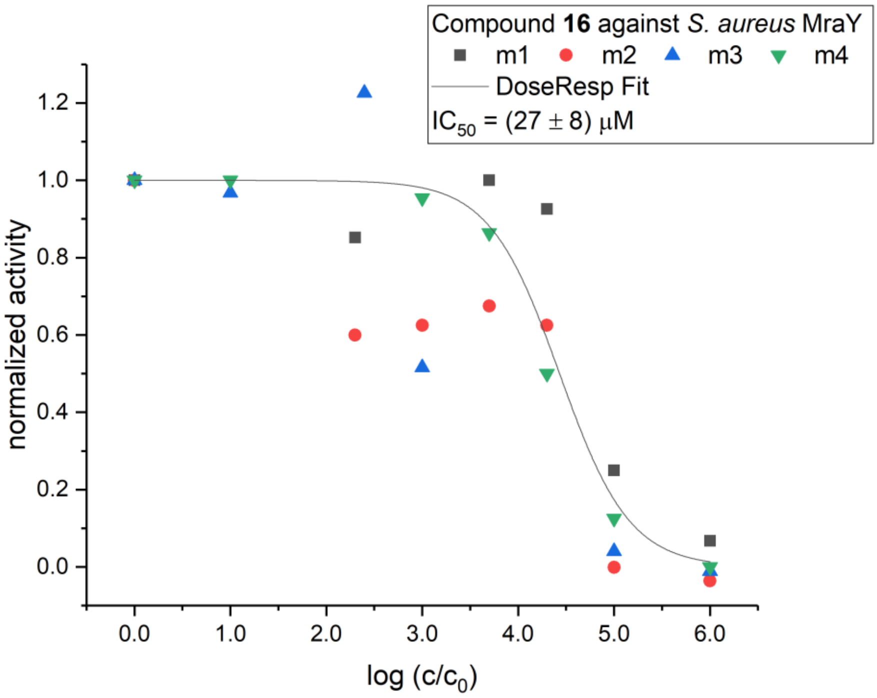

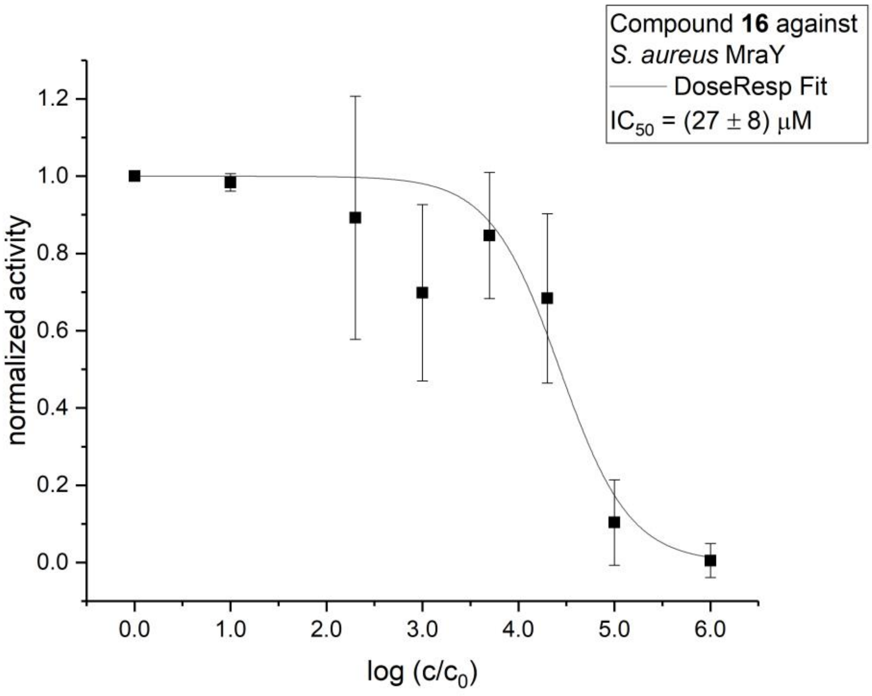

Figure S4. MraY assay with MraY from *S. aureus* (crude membranes) and compound **16** as inhibitor, including the fitted curve for MraY activity (top: original data used for fitting the curve, with data points shown as individual series of measurements; bottom: values displayed as mean values and standard deviations, for illustrative purposes; c_0 = 1 nM).

^{1}H, ^{13}C and ^{19}F NMR spectra of synthesized compounds

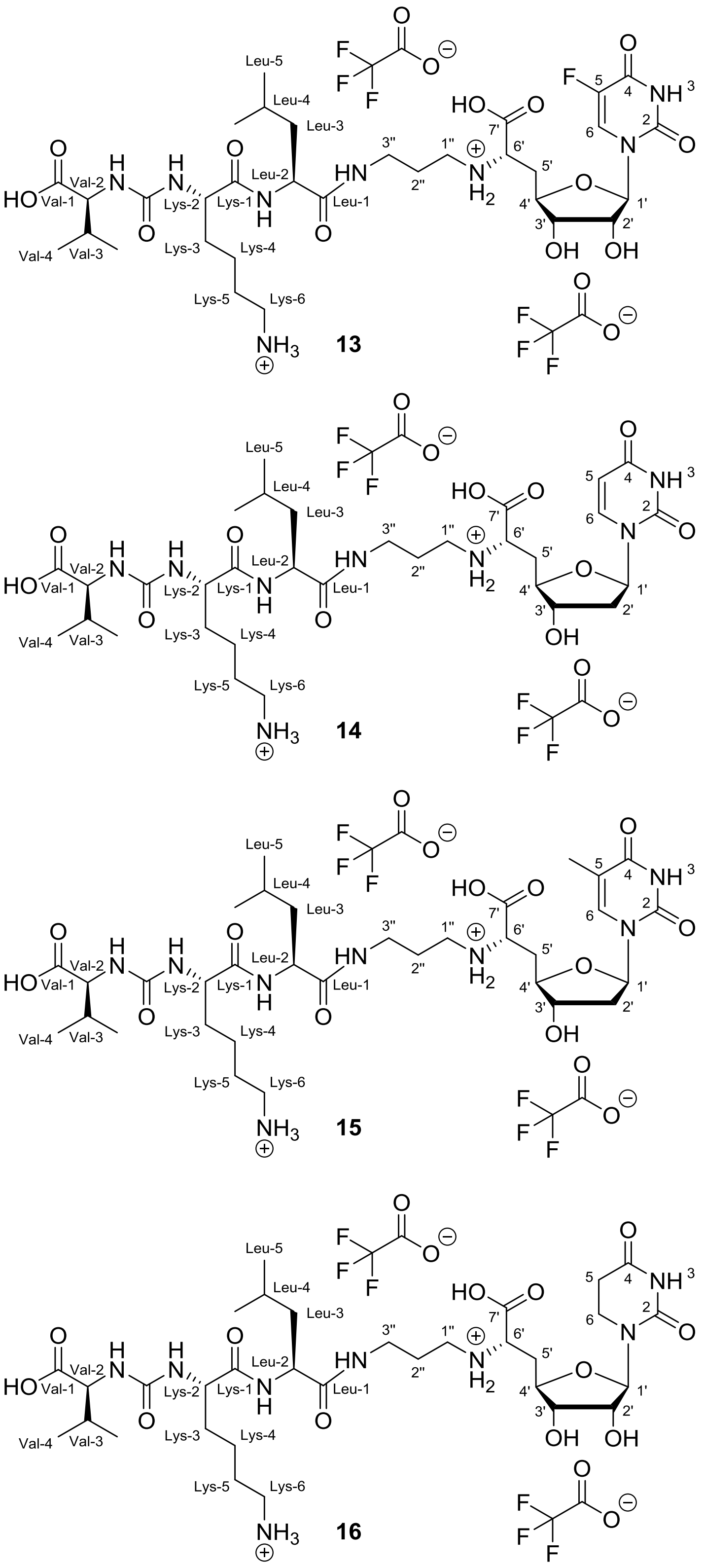

Figure S5. Numbering of atoms of muraymycin target structures **13-16** for the assignment of NMR signals.

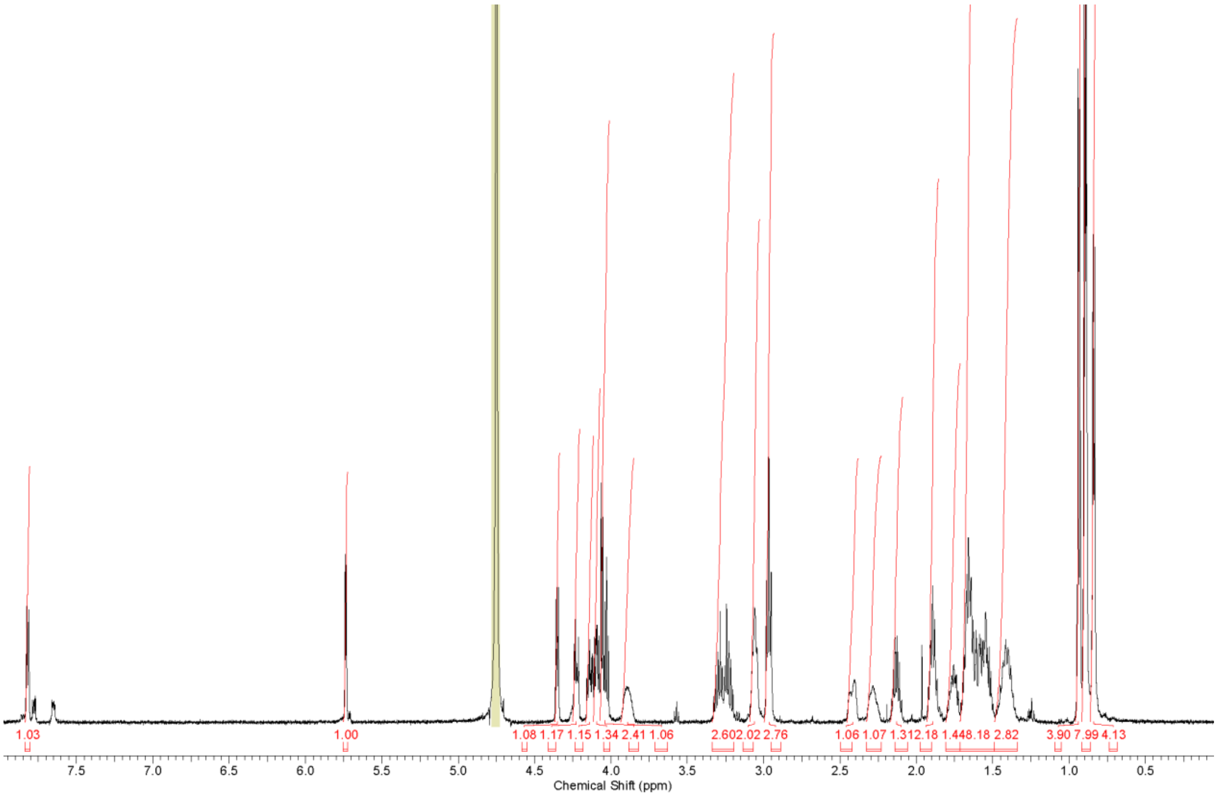

^{1}H NMR spectrum of **13** (500 MHz, D_2O).

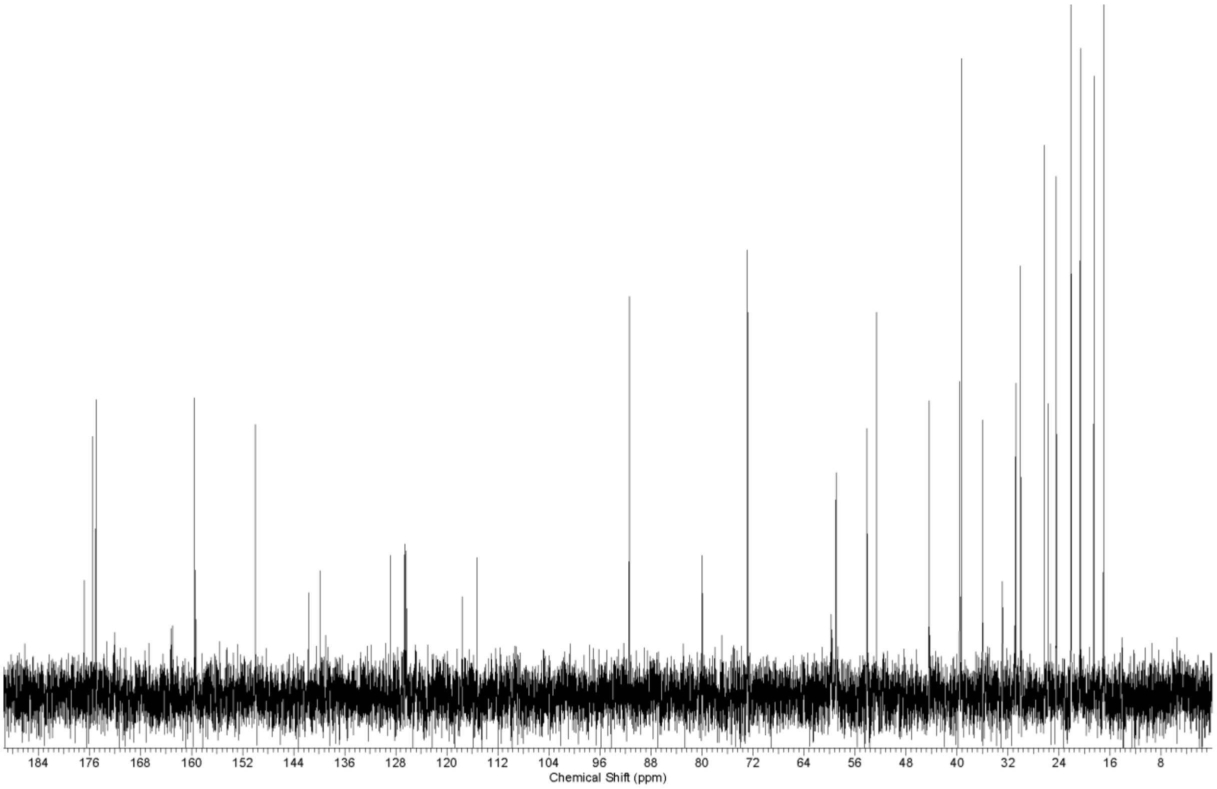

^{13}C NMR spectrum of **13** (126 MHz, D_2O).

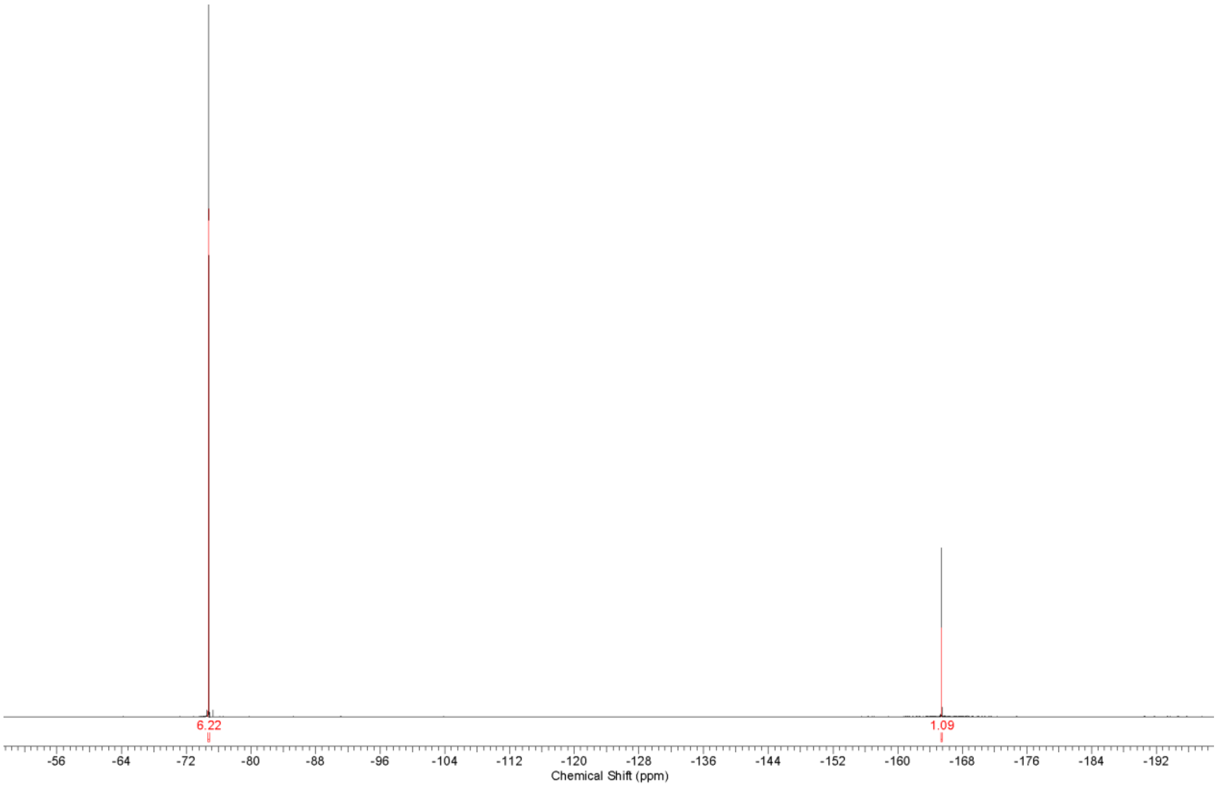

^{19}F NMR spectrum of **13** (376 MHz, D_2O).

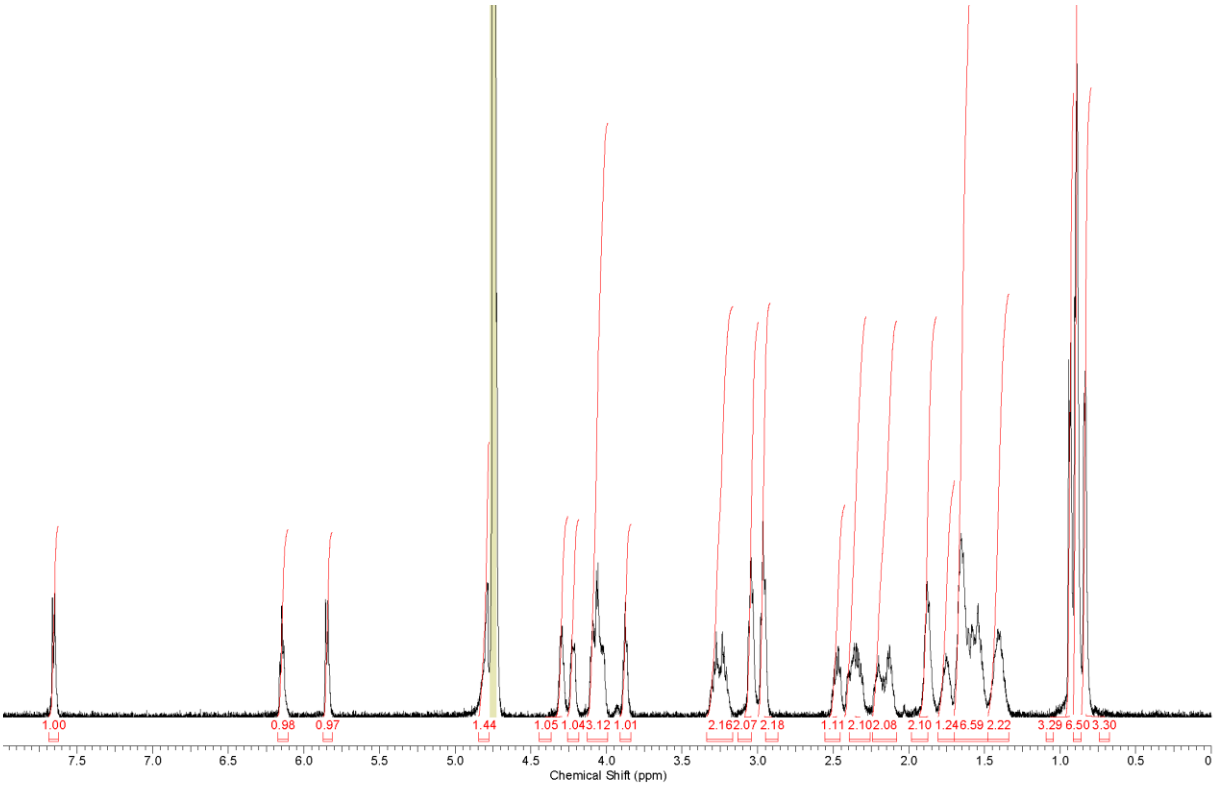

^{1}H NMR spectrum of **14** (500 MHz, D_2O).

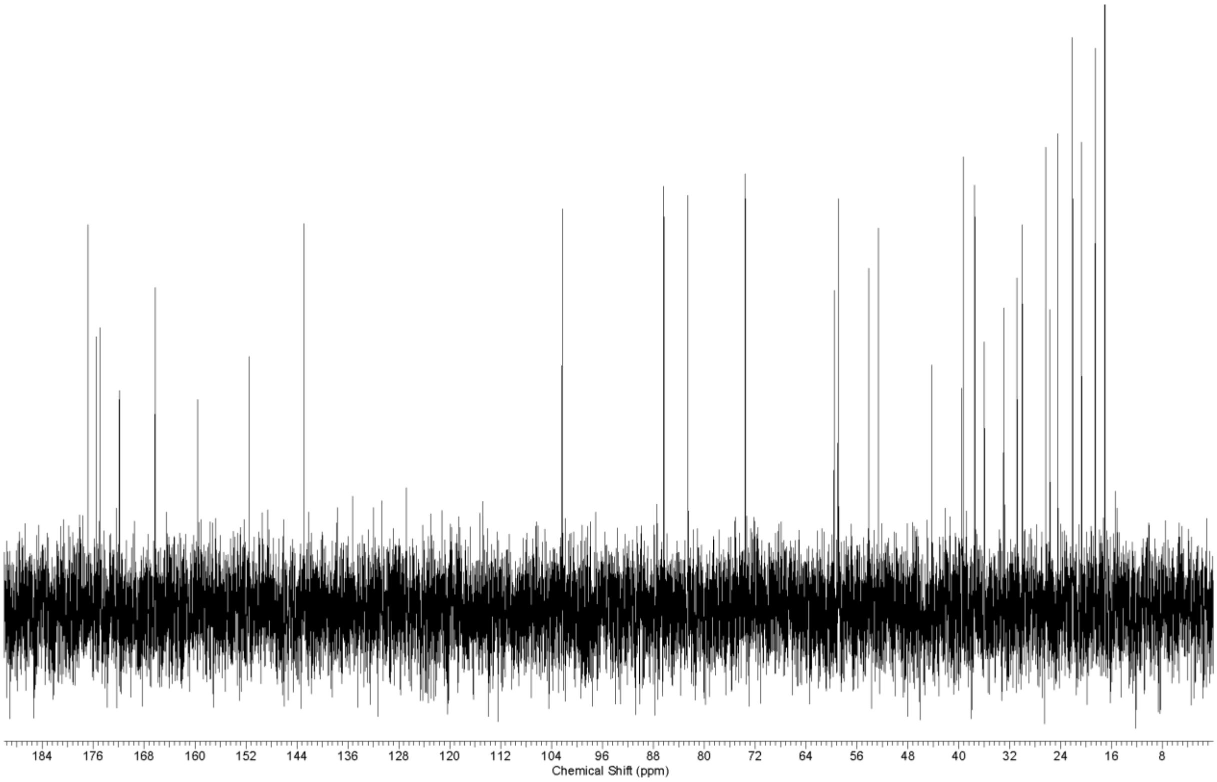

^{13}C NMR spectrum of **14** (126 MHz, D_2O)

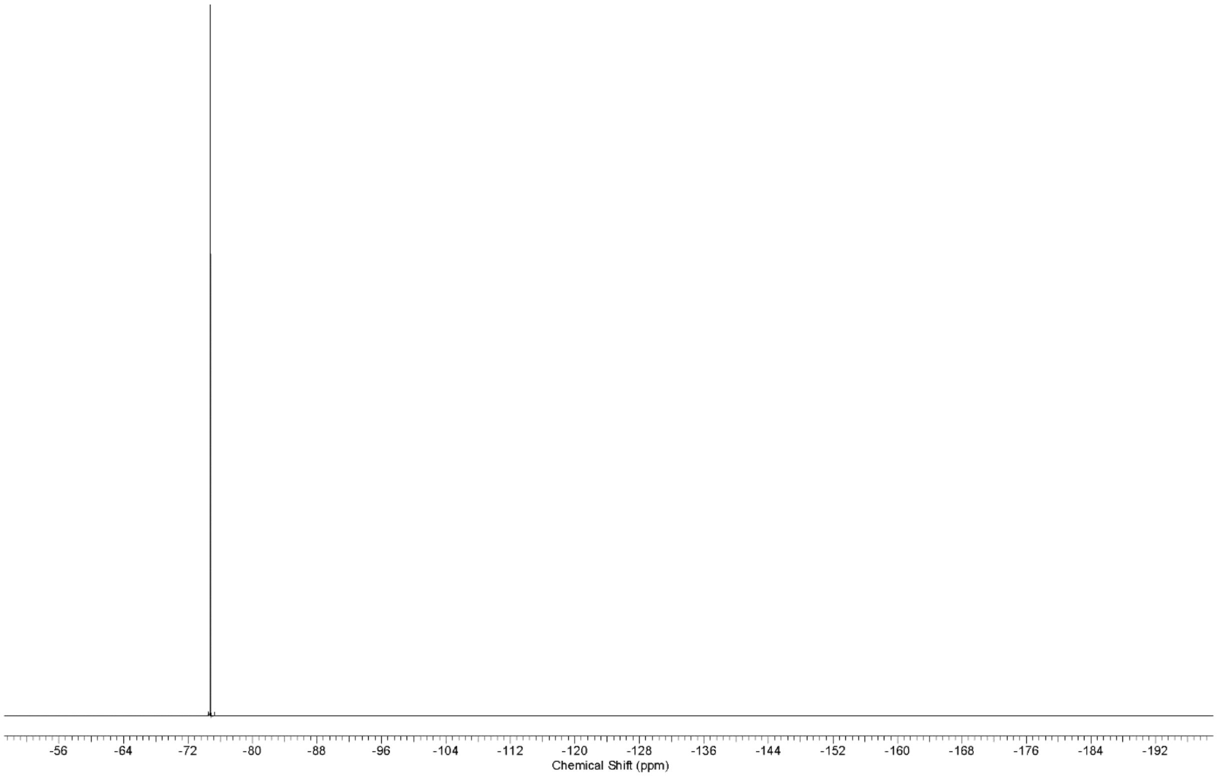

^{19}F NMR spectrum of **14** (376 MHz, D_2O).

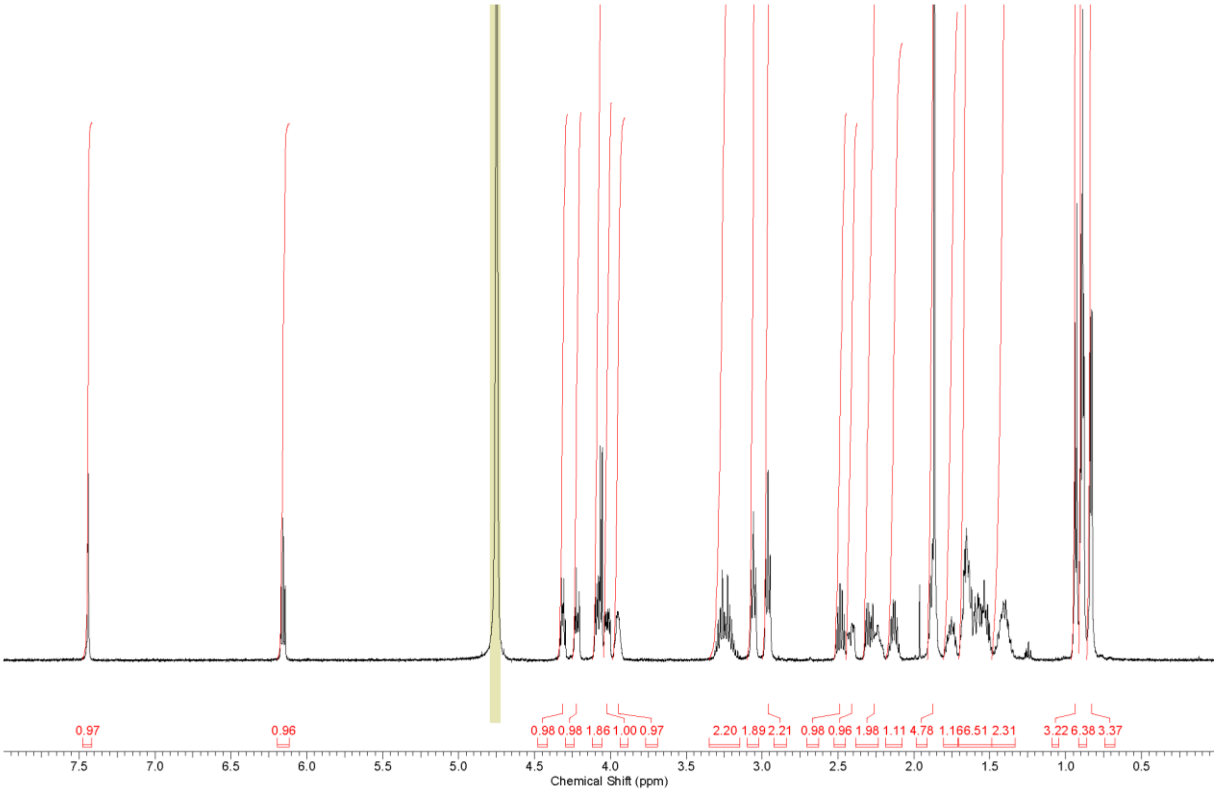

^{1}H NMR spectrum of **15** (500 MHz, D_2O).

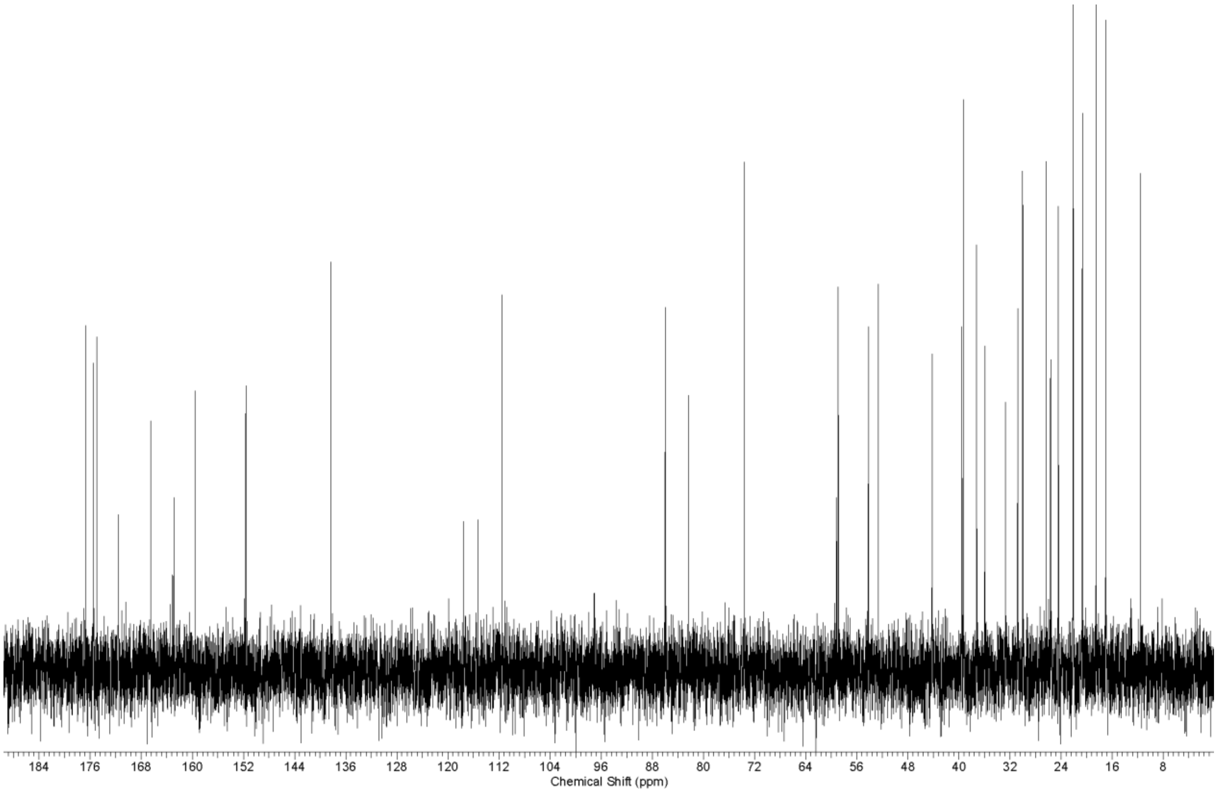

^{13}C NMR spectrum of **15** (126 MHz, D_2O).

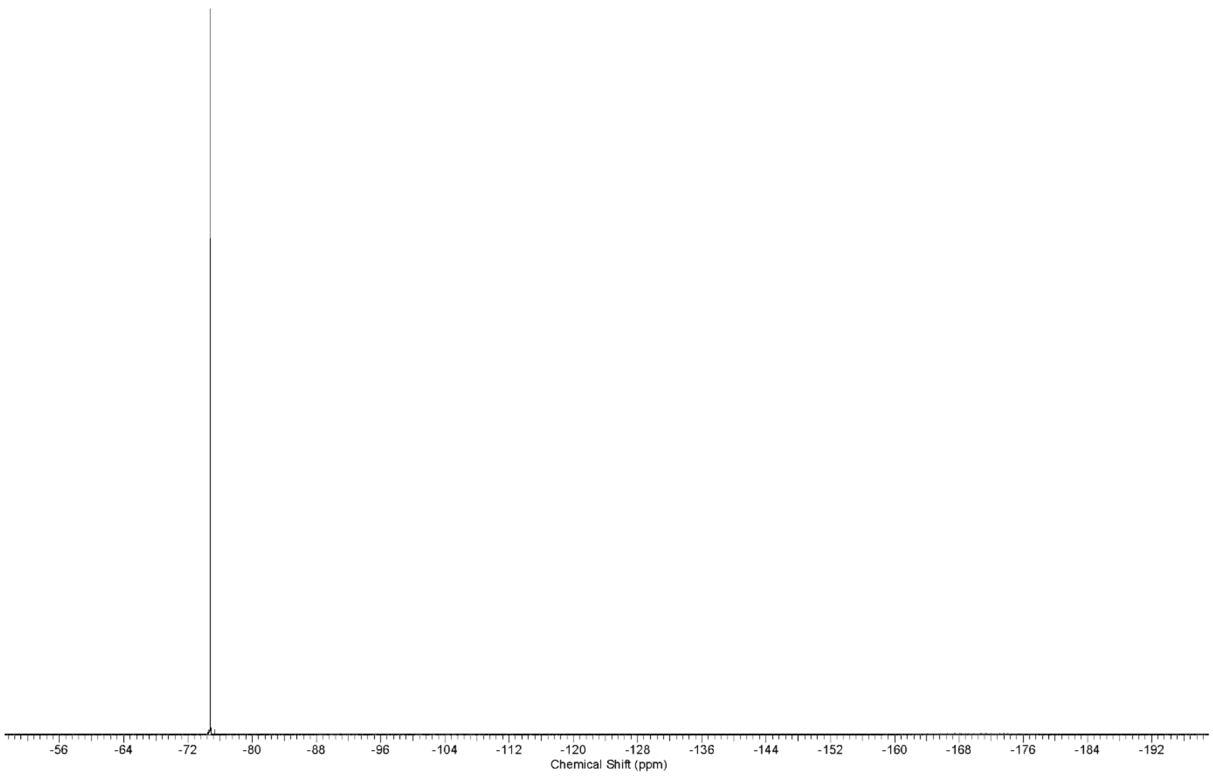

^{19}F NMR spectrum of **15** (376 MHz, D_2O).

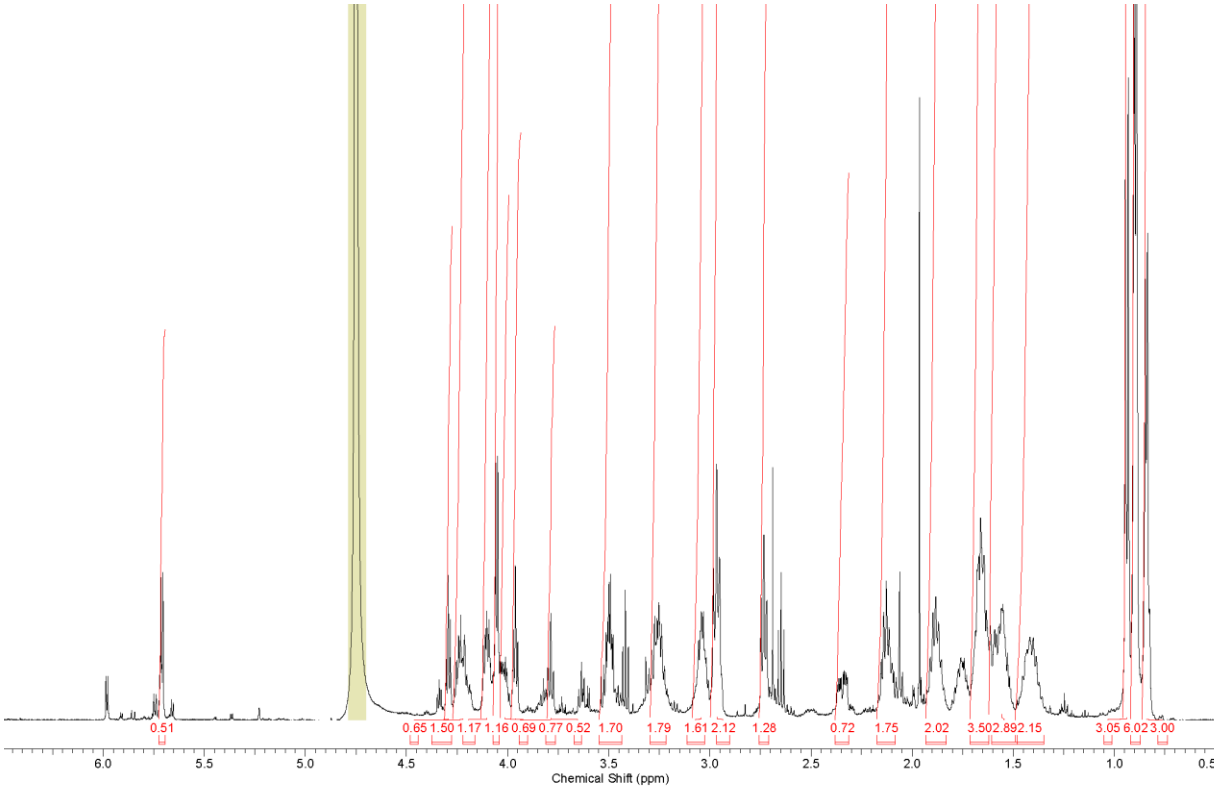

^{1}H NMR spectrum of **16** (500 MHz, D_2O).

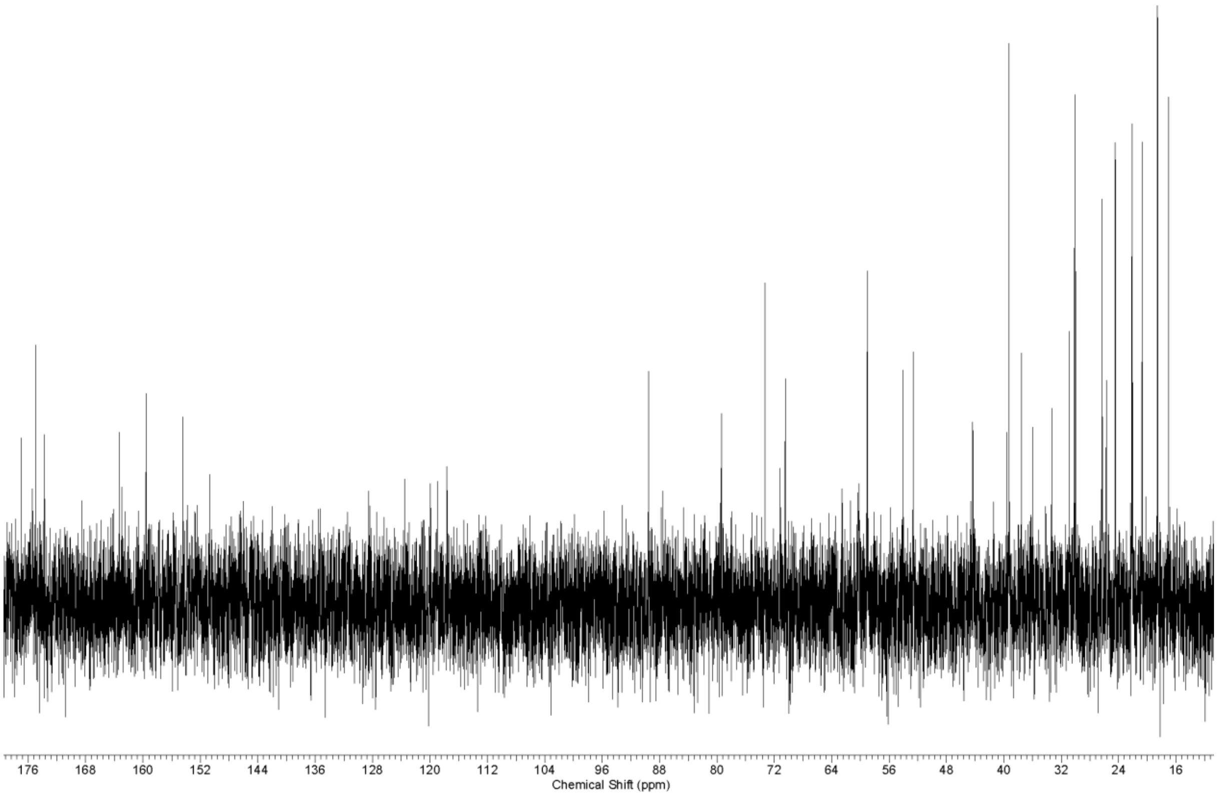

^{13}C NMR spectrum of **16** (126 MHz, D_2O).

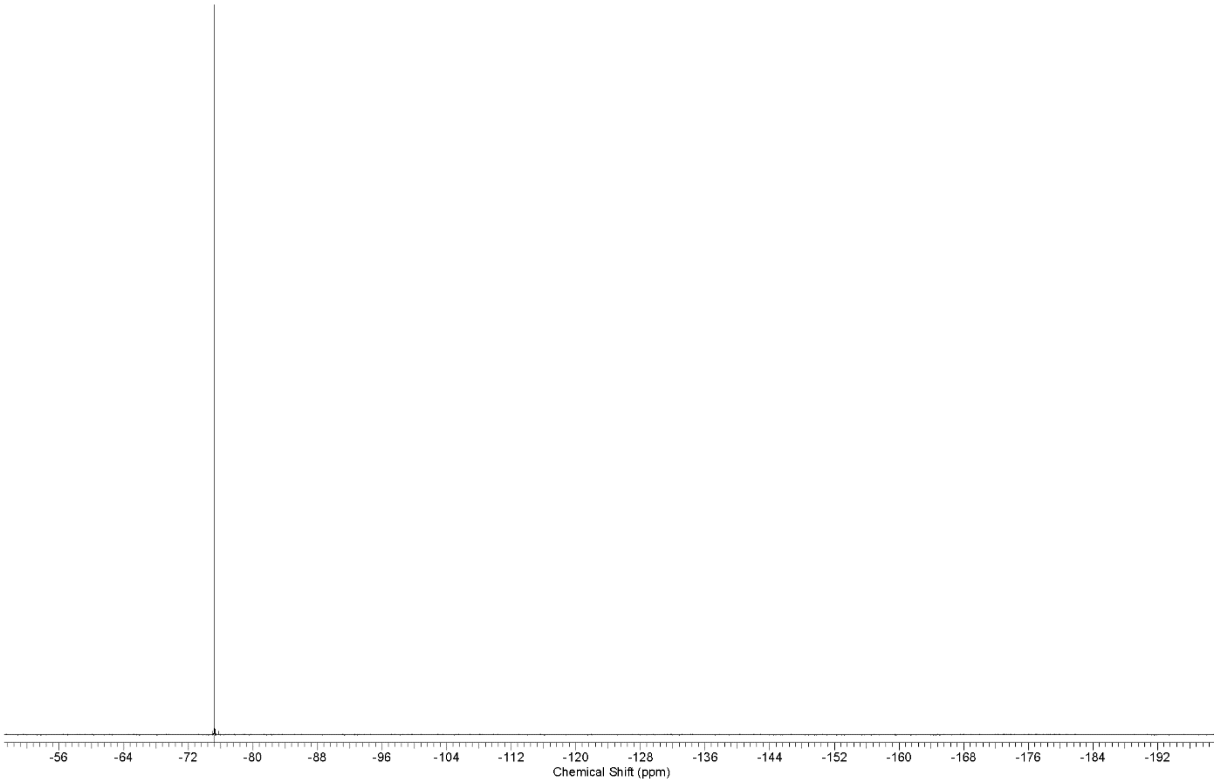

19F NMR spectrum of **16** (376 MHz, D_2O).

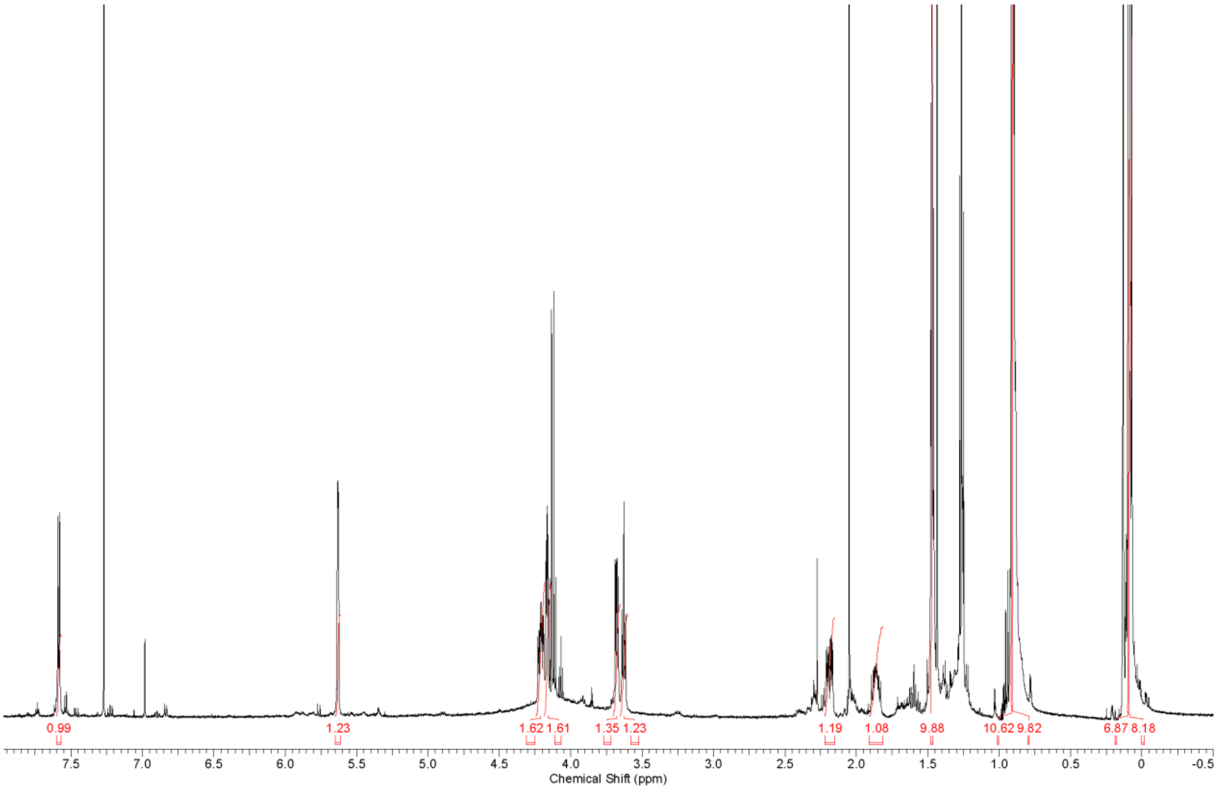

^{1}H NMR spectrum of **18** (500 MHz, $CDCl_3$).

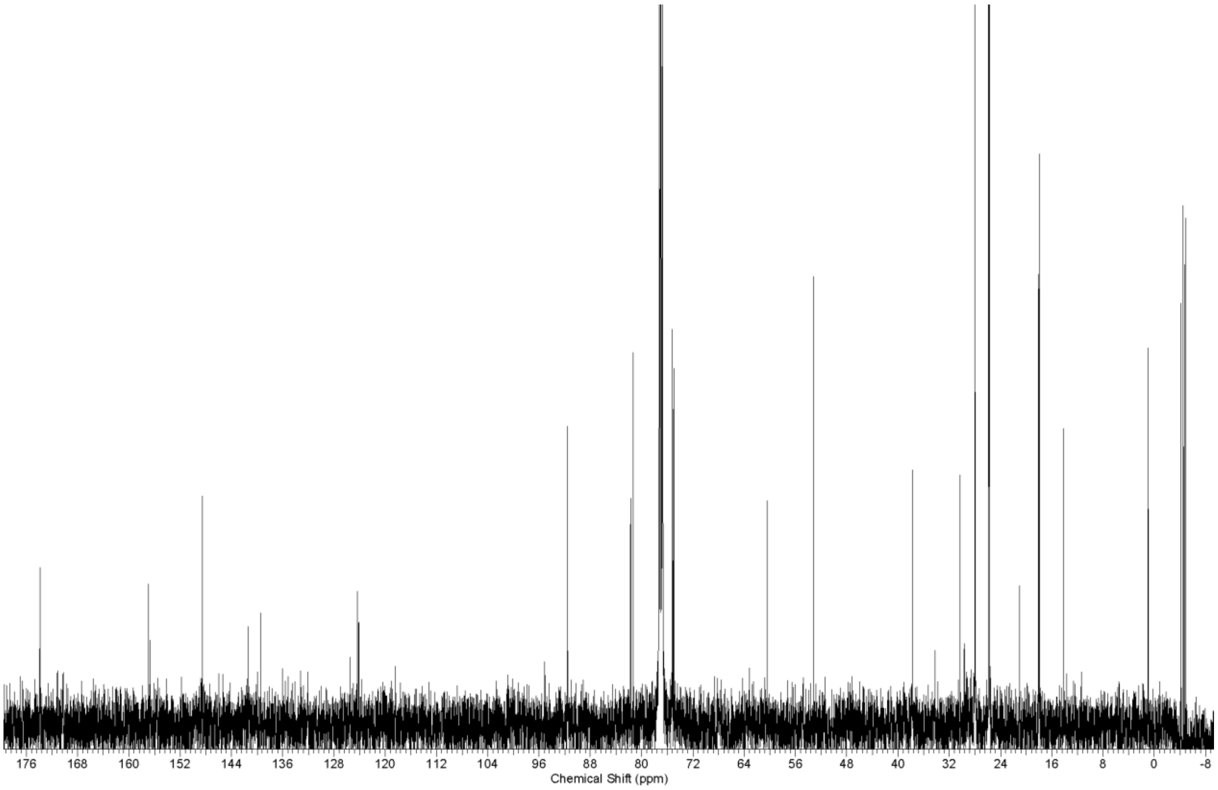

^{13}C NMR spectrum of **18** (126 MHz, $CDCl_3$).

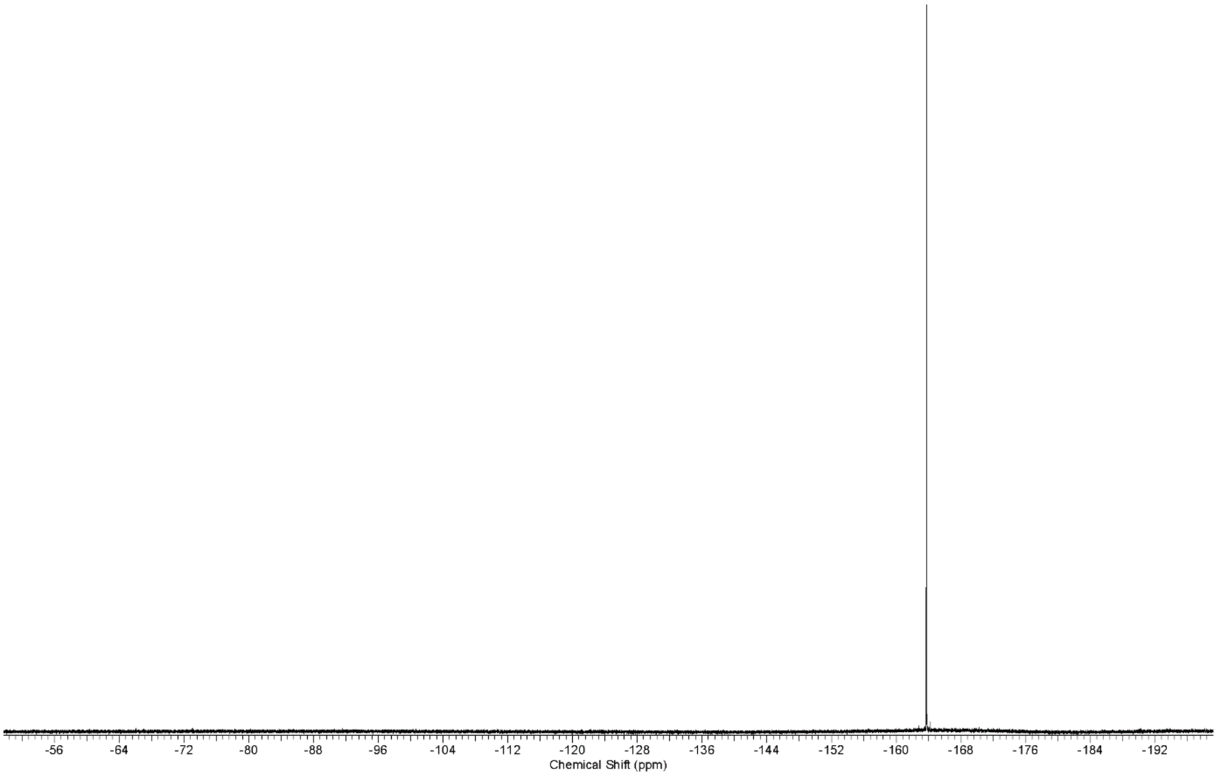

^{19}F NMR spectrum of **18** (376 MHz, $CDCl_3$).

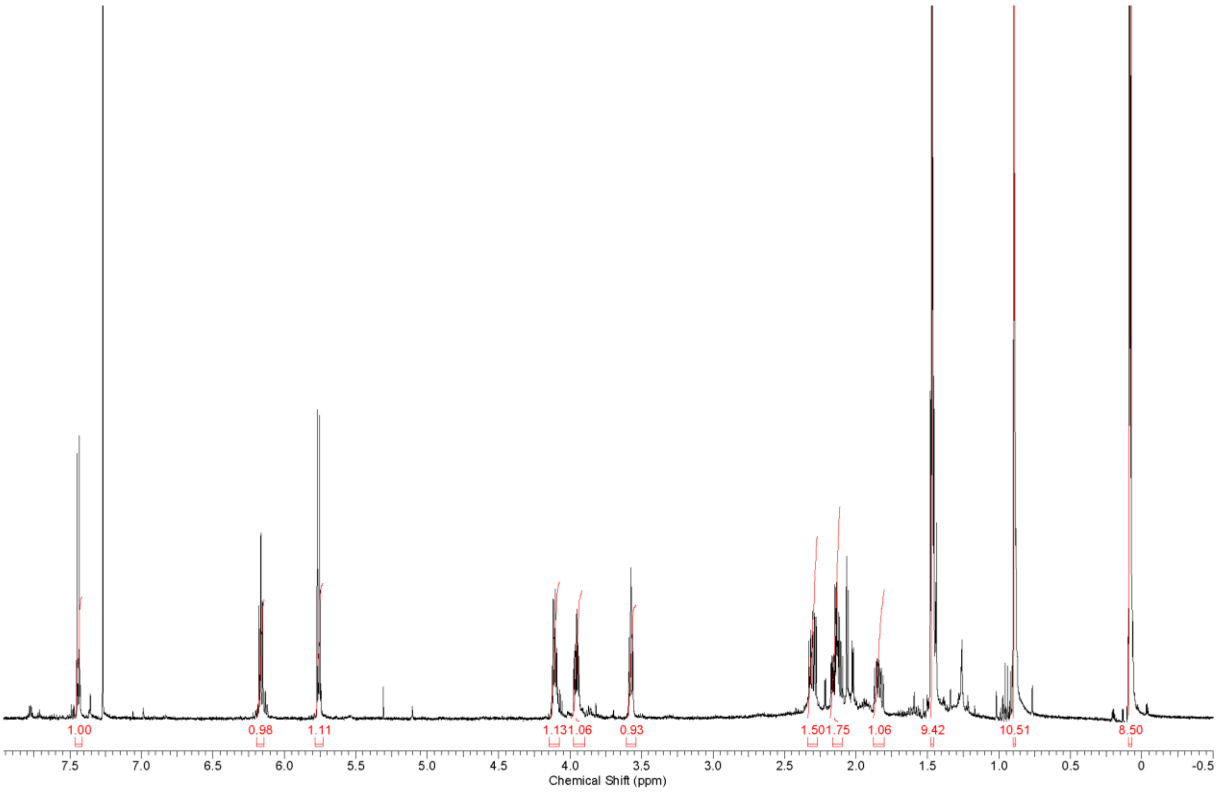

^{1}H NMR spectrum of **19** (500 MHz, $CDCl_3$).

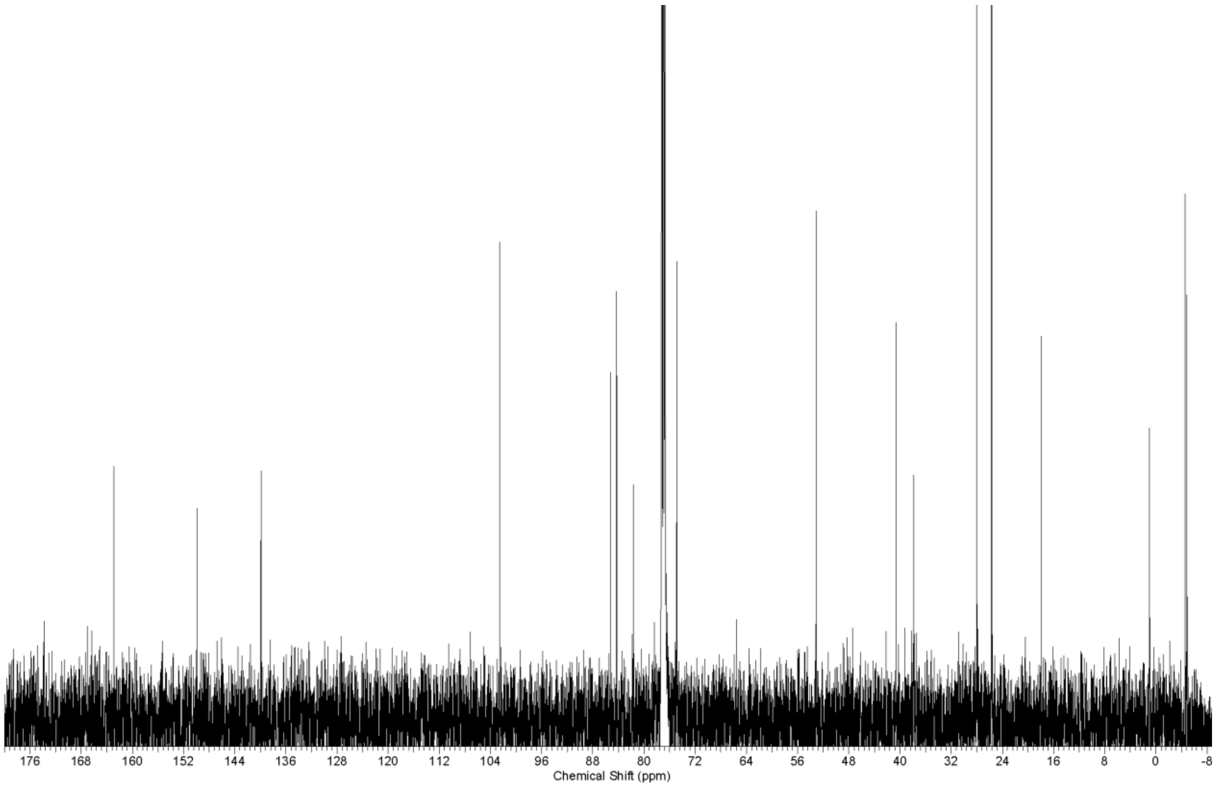

^{13}C NMR spectrum of **19** (126 MHz, $CDCl_3$).

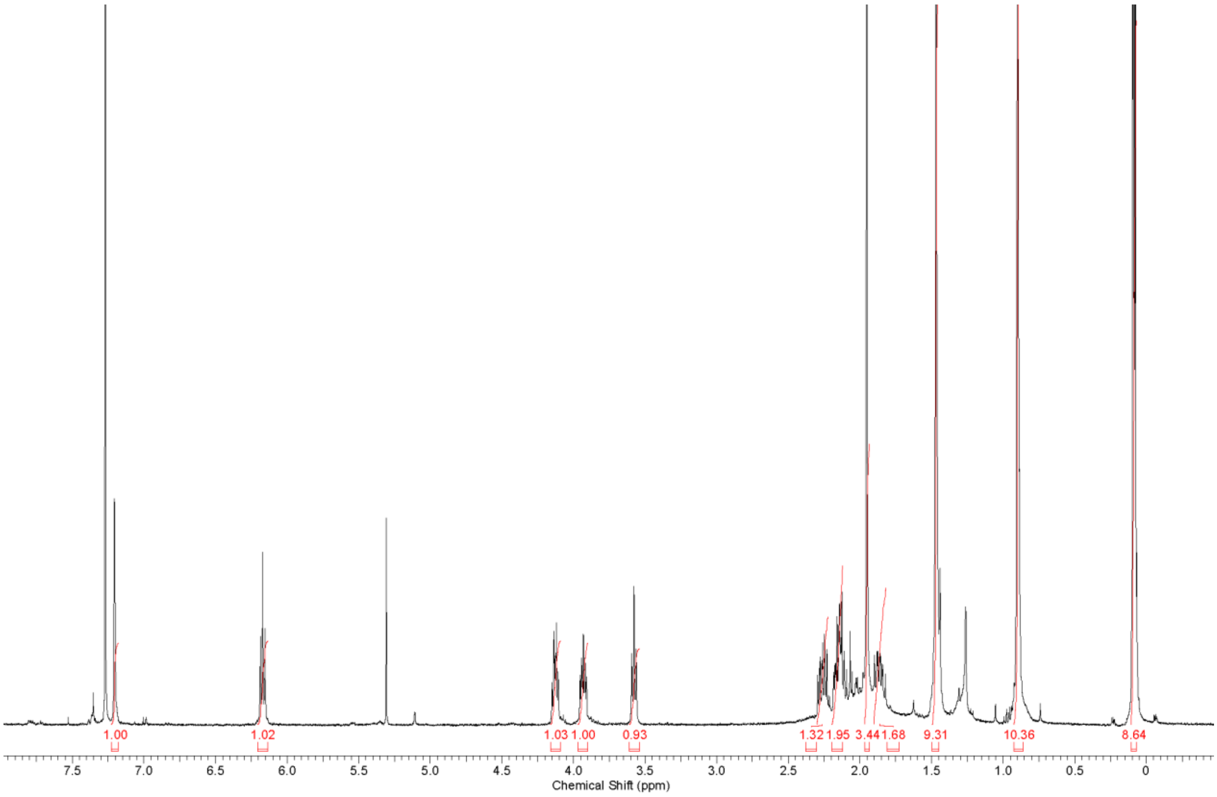

^{1}H NMR spectrum of **20** (500 MHz, $CDCl_3$).

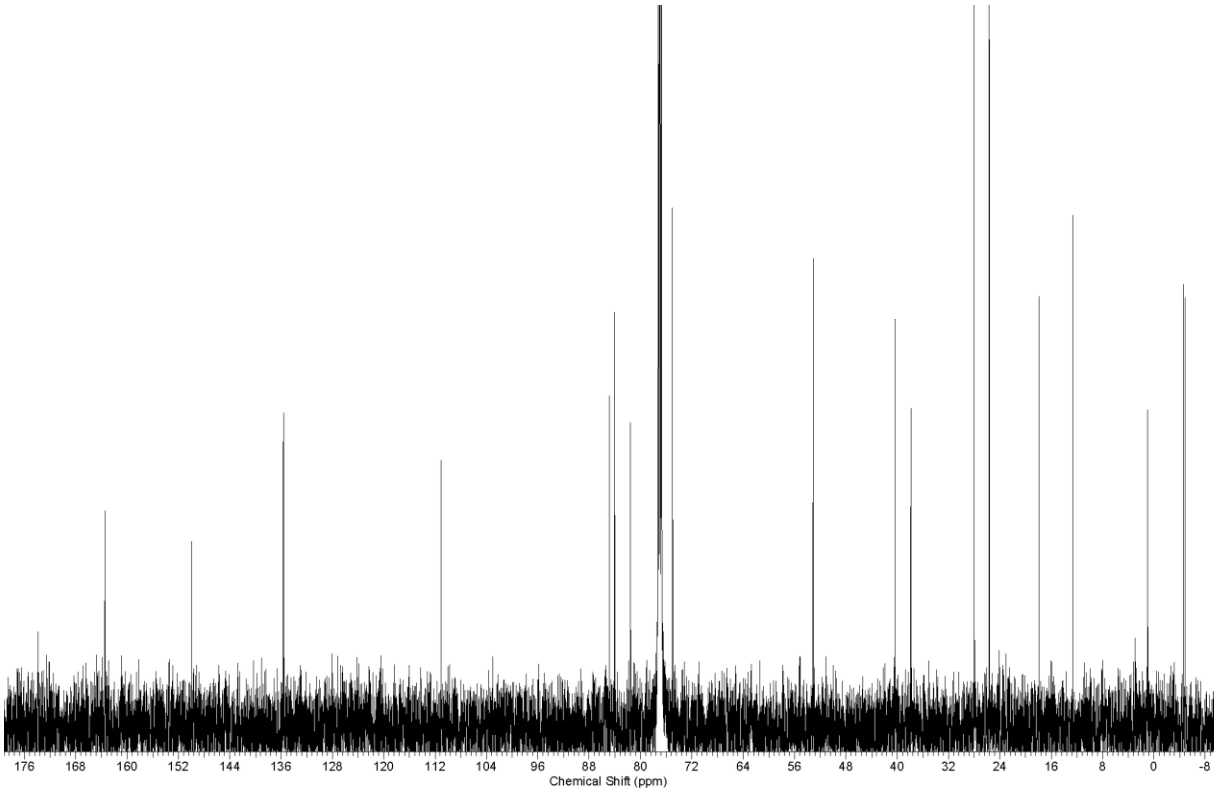

^{13}C NMR spectrum of **20** (126 MHz, $CDCl_3$).

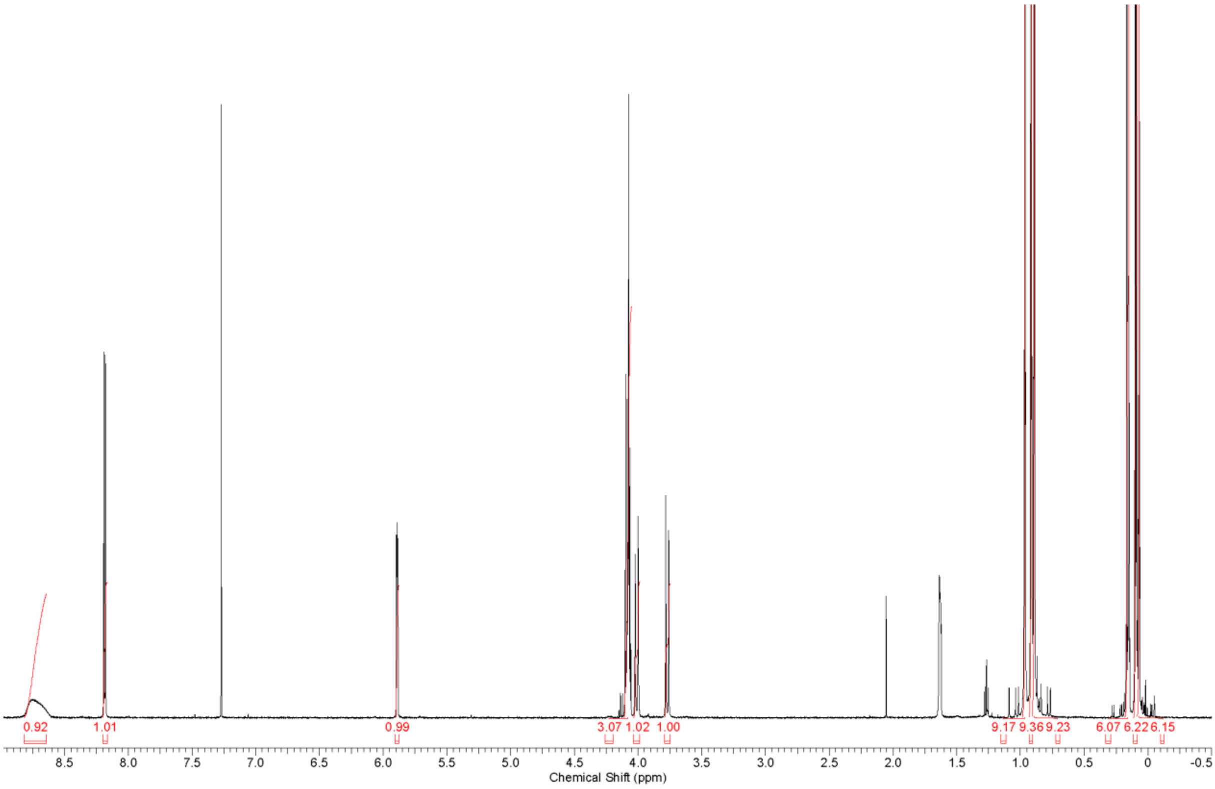

^{1}H NMR spectrum of **24** (500 MHz, $CDCl_3$).

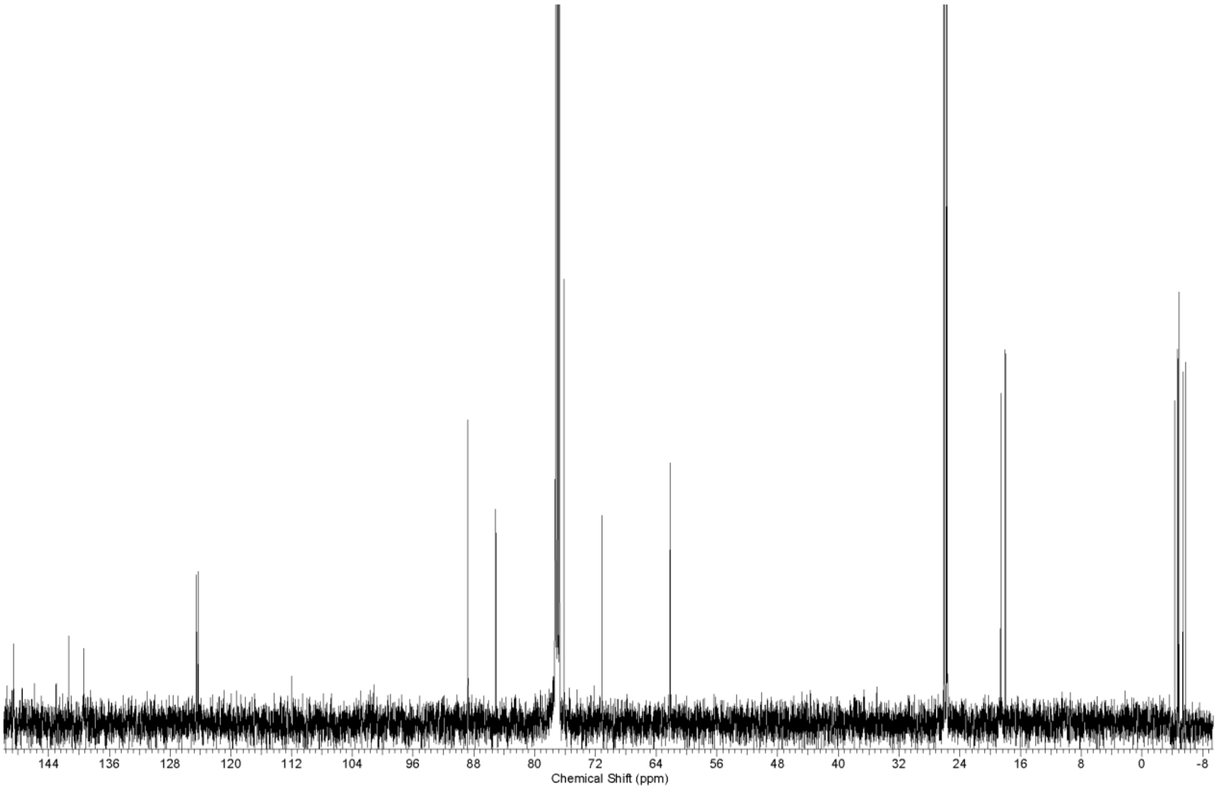

^{13}C NMR spectrum of **24** (126 MHz, $CDCl_3$).

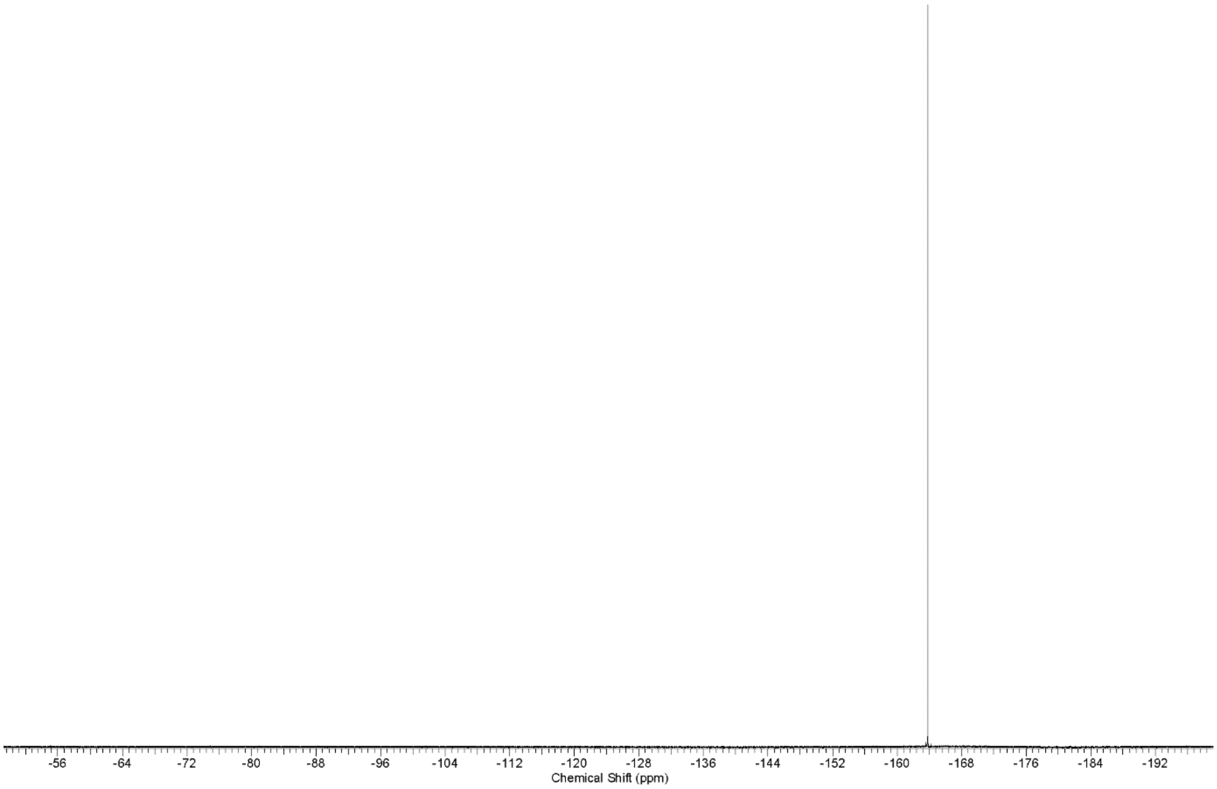

^{19}F NMR spectrum of **24** (376 MHz, $CDCl_3$).

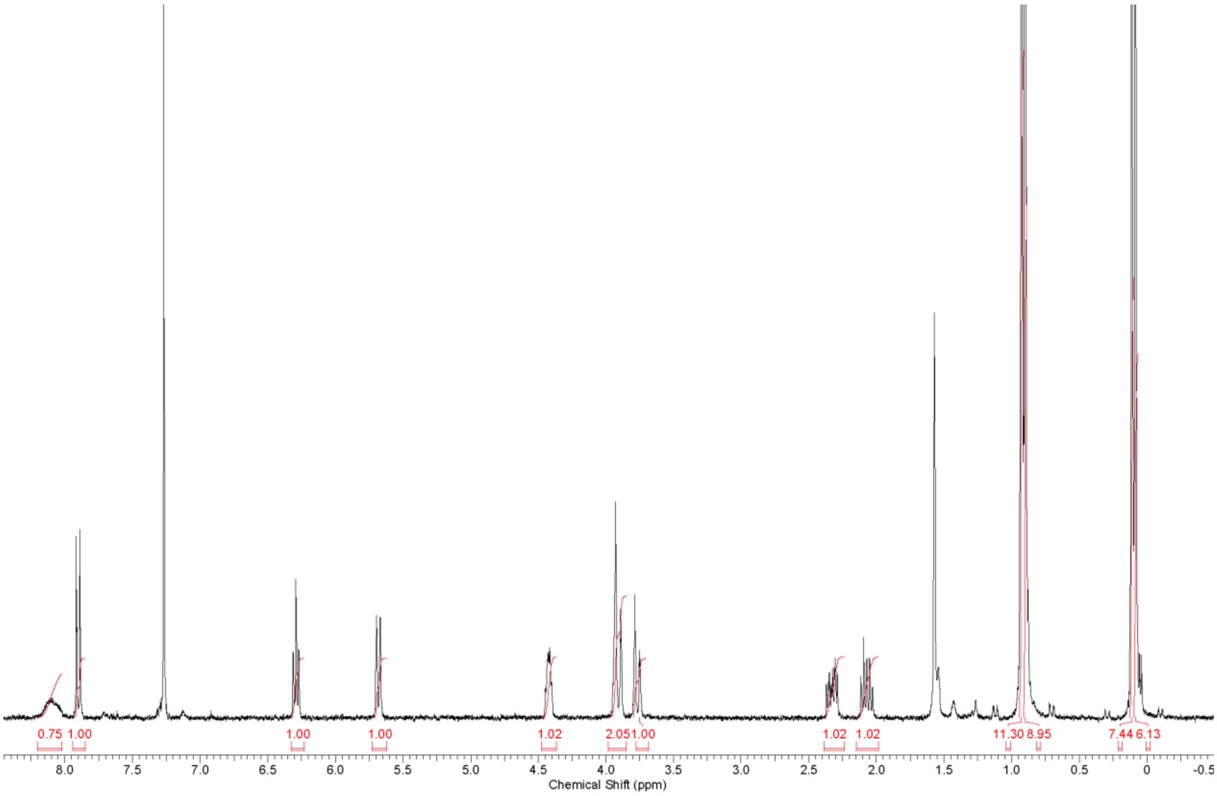

^{1}H NMR spectrum of **25** (300 MHz, $CDCl_3$).

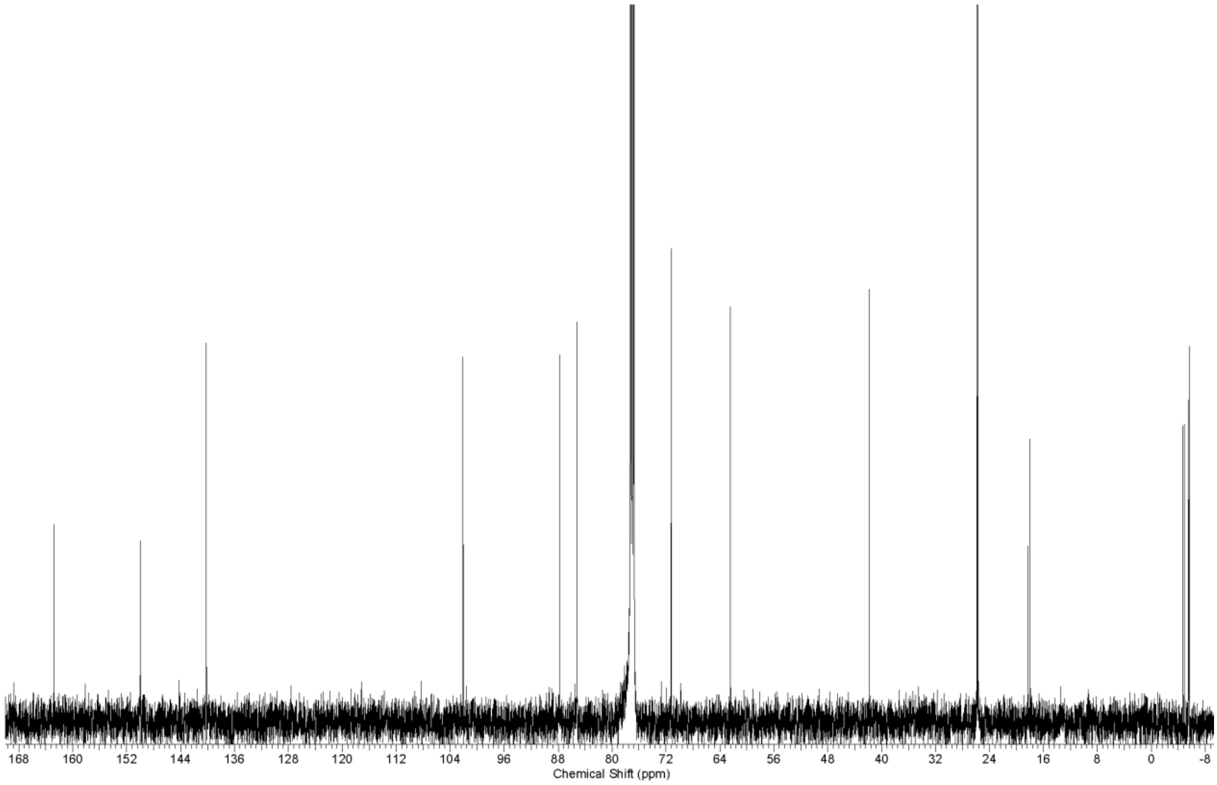

^{13}C NMR spectrum of **25** (126 MHz, $CDCl_3$).

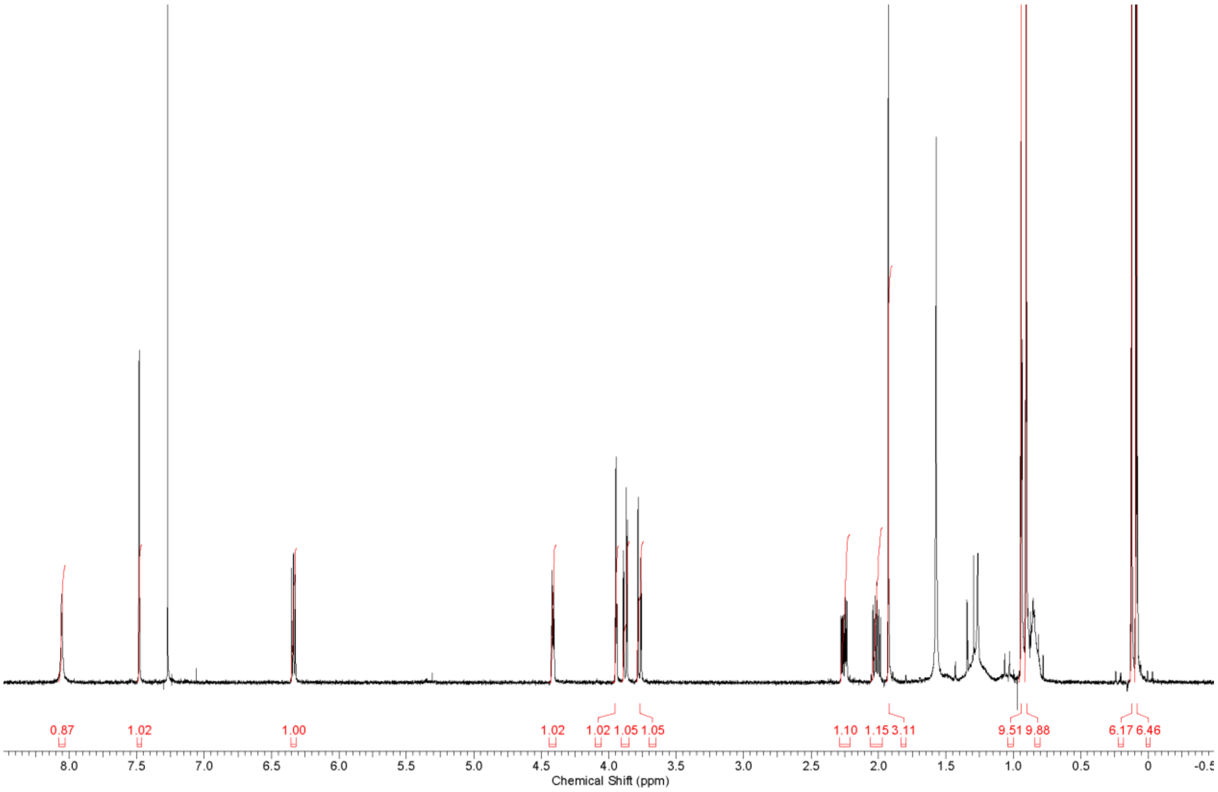

^{1}H NMR spectrum of **26** (300 MHz, $CDCl_3$).

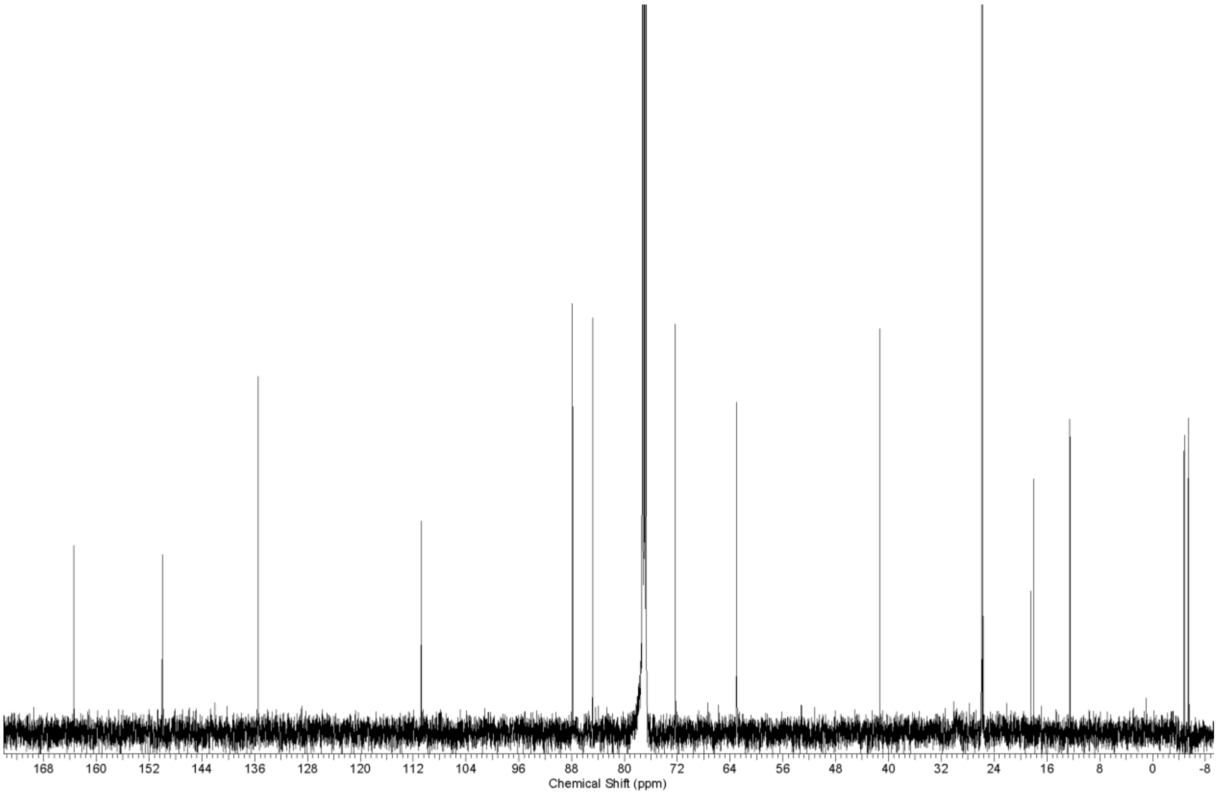

^{13}C NMR spectrum of **26** (126 MHz, $CDCl_3$).

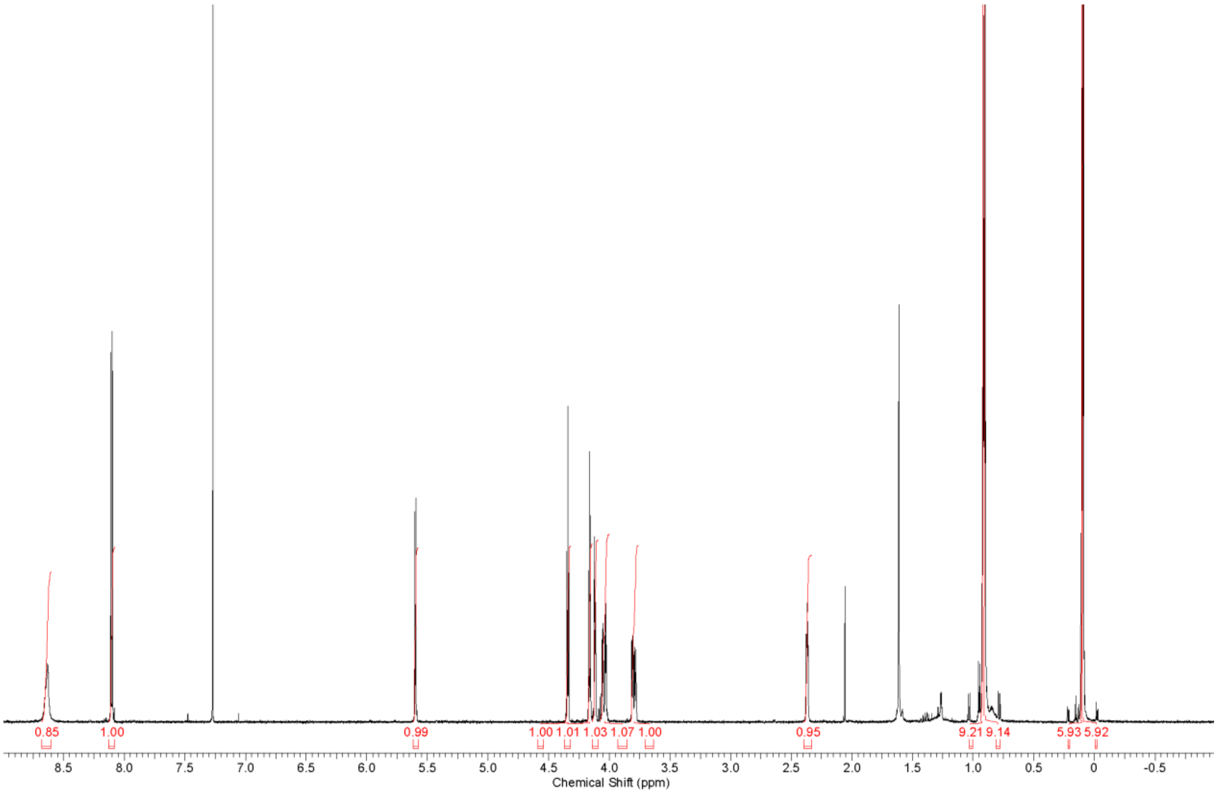

^{1}H NMR spectrum of **27** (500 MHz, $CDCl_3$).

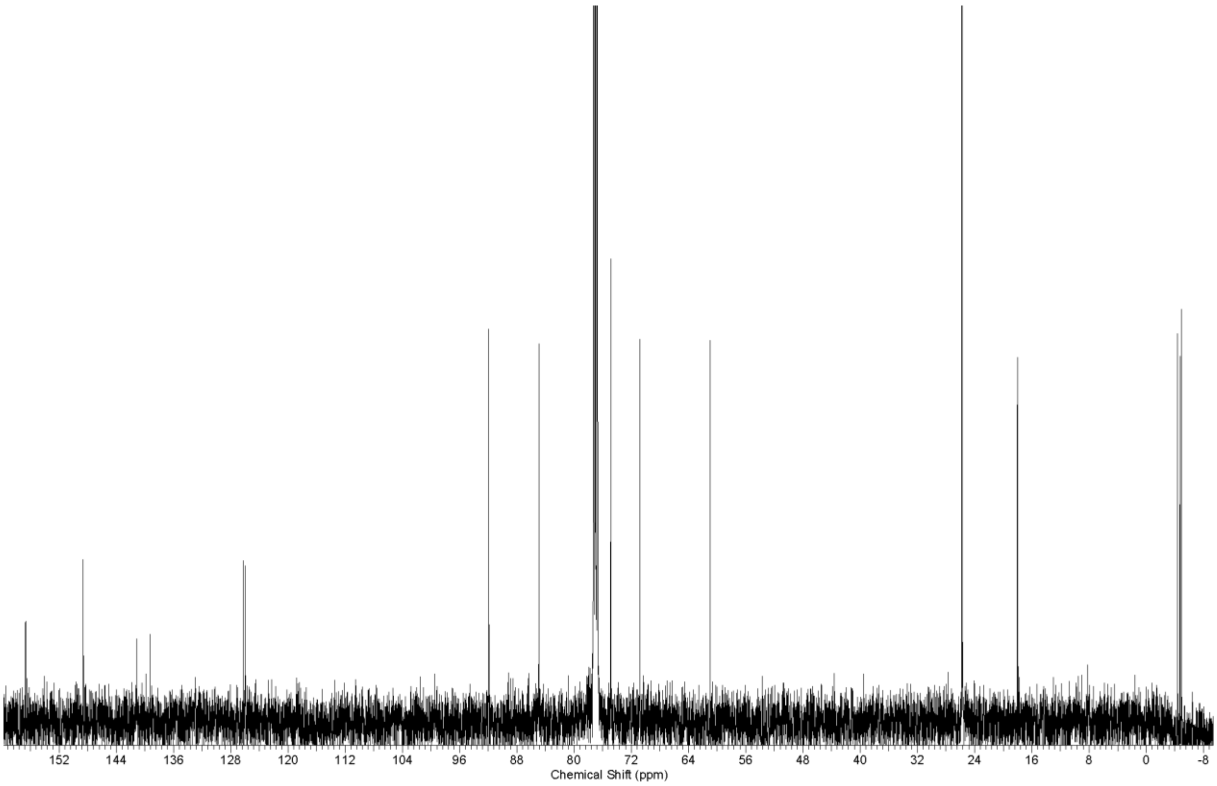

^{13}C NMR spectrum of **27** (126 MHz, $CDCl_3$).

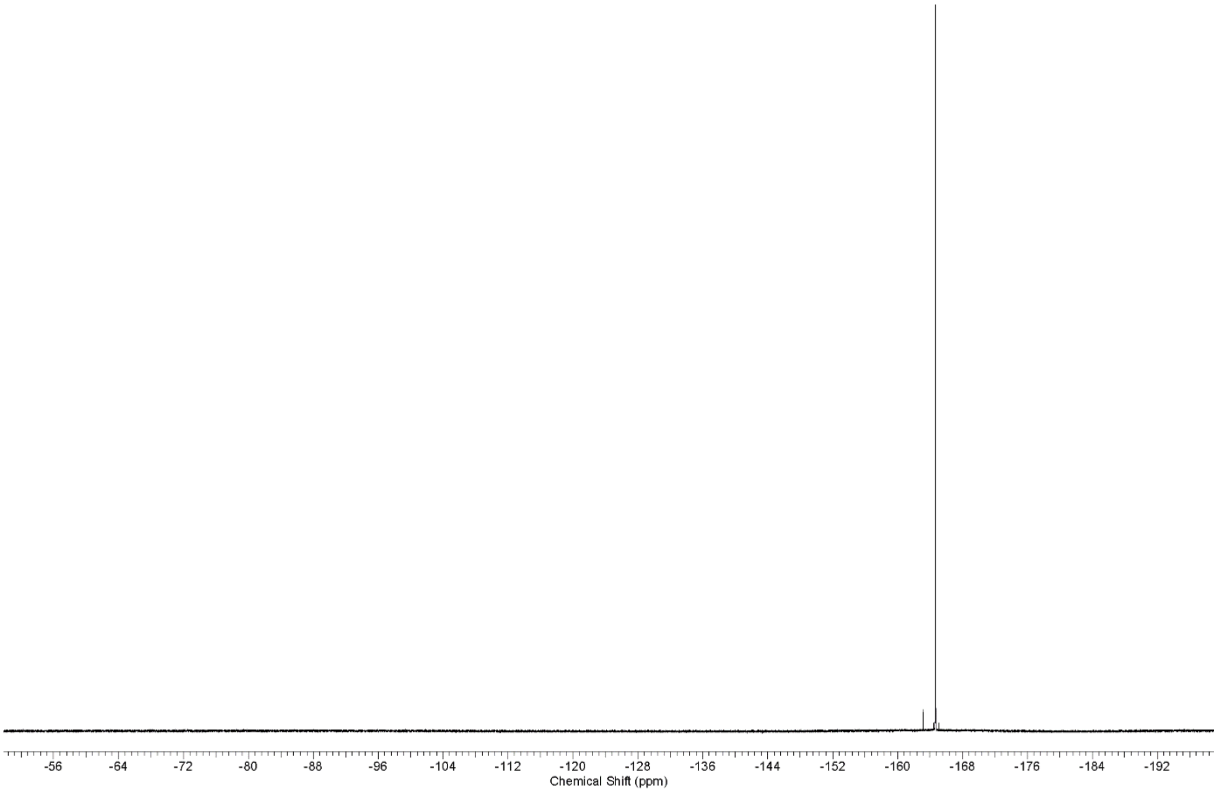

^{19}F NMR spectrum of **27** (376 MHz, $CDCl_3$).

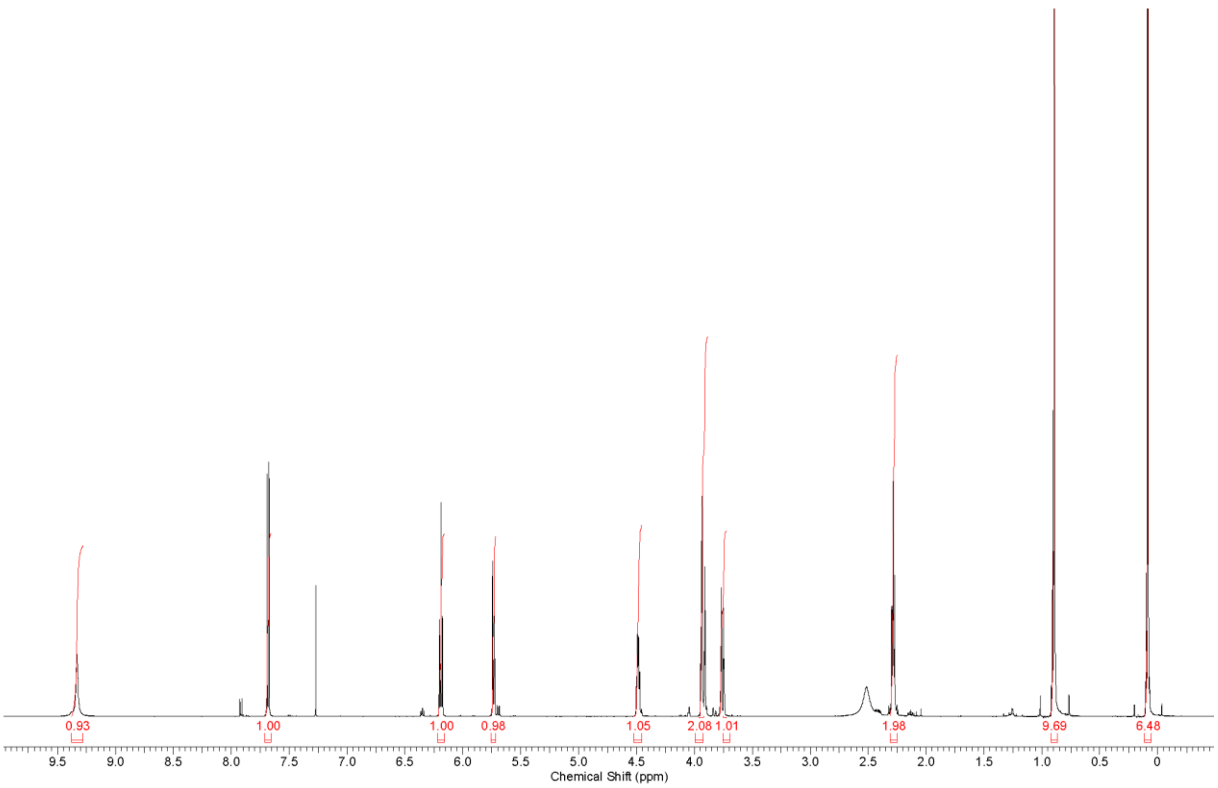

^{1}H NMR spectrum of **28** (500 MHz, $CDCl_3$).

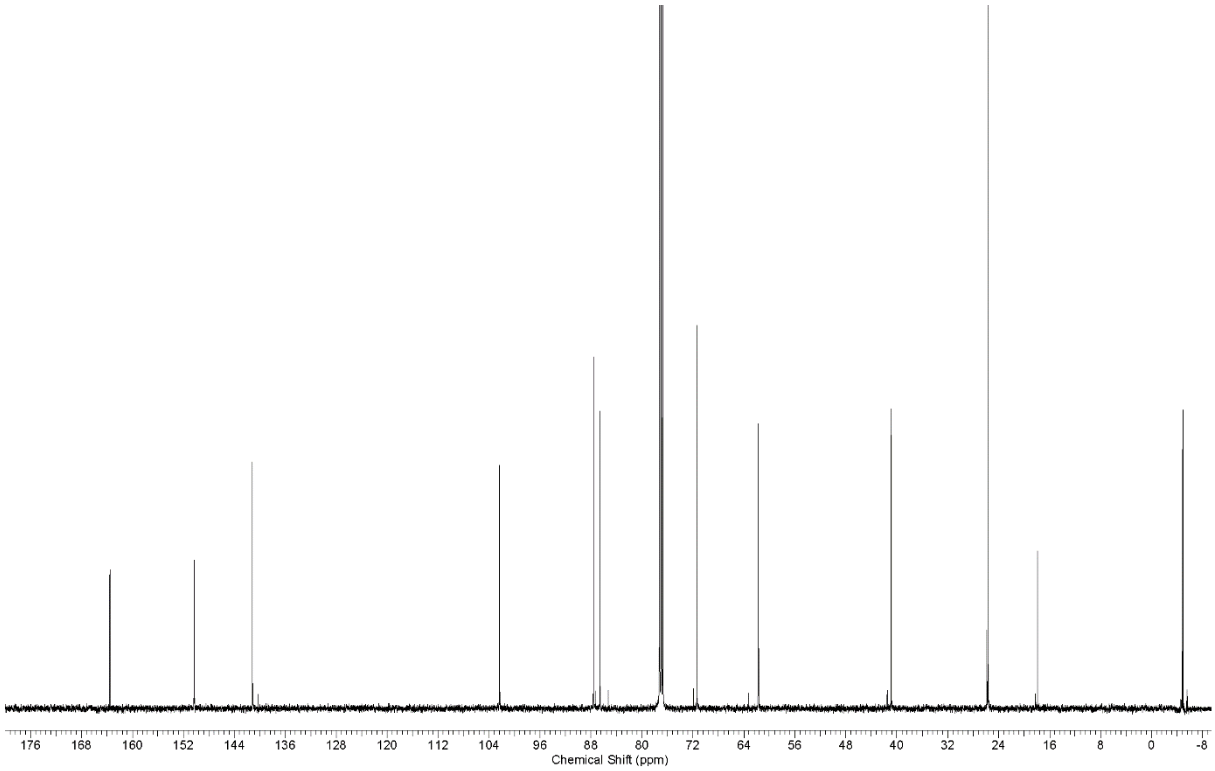

^{13}C NMR spectrum of **28** (126 MHz, $CDCl_3$).

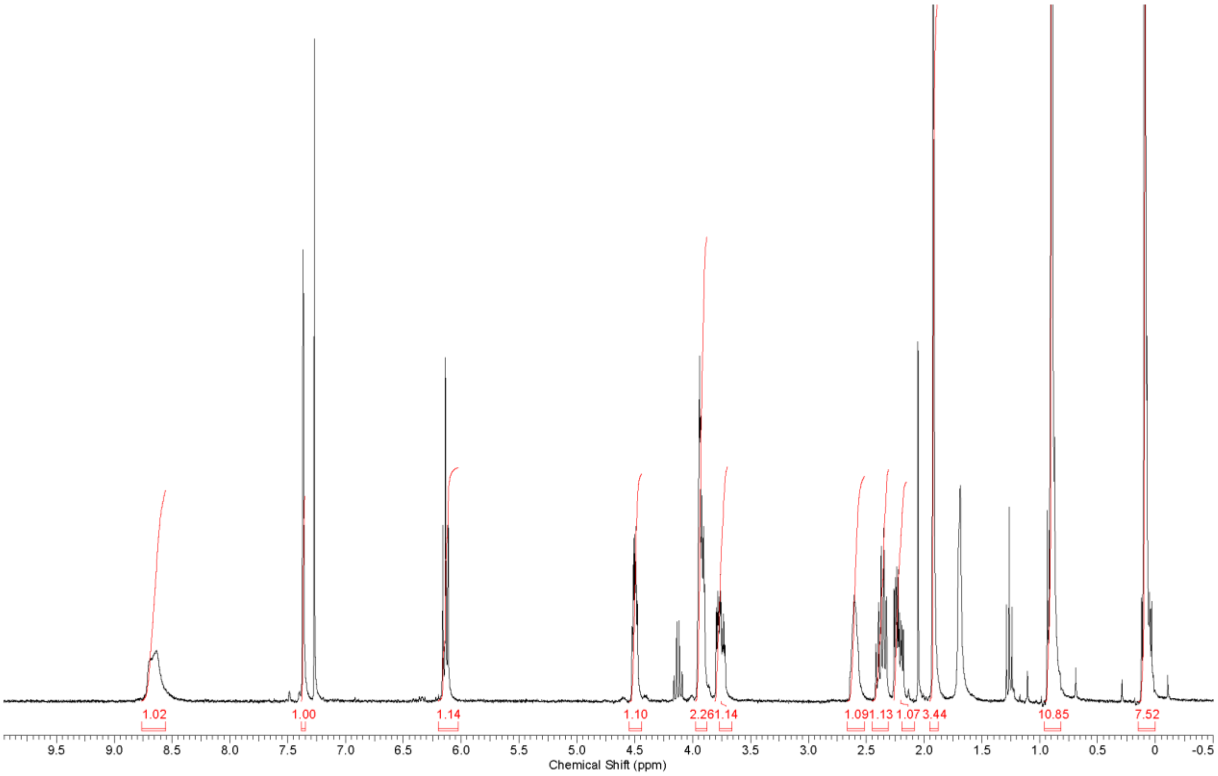

^{1}H NMR spectrum of **29** (500 MHz, $CDCl_3$).

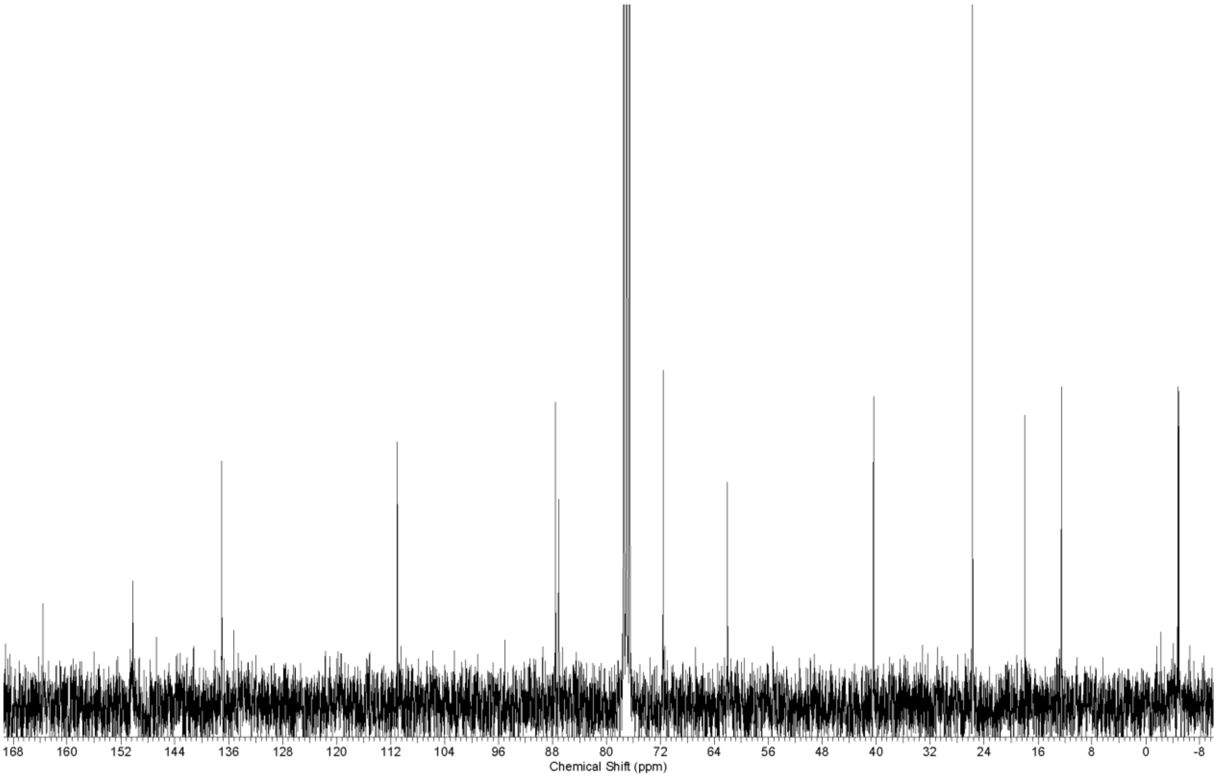

^{13}C NMR spectrum of **29** (126 MHz, $CDCl_3$).

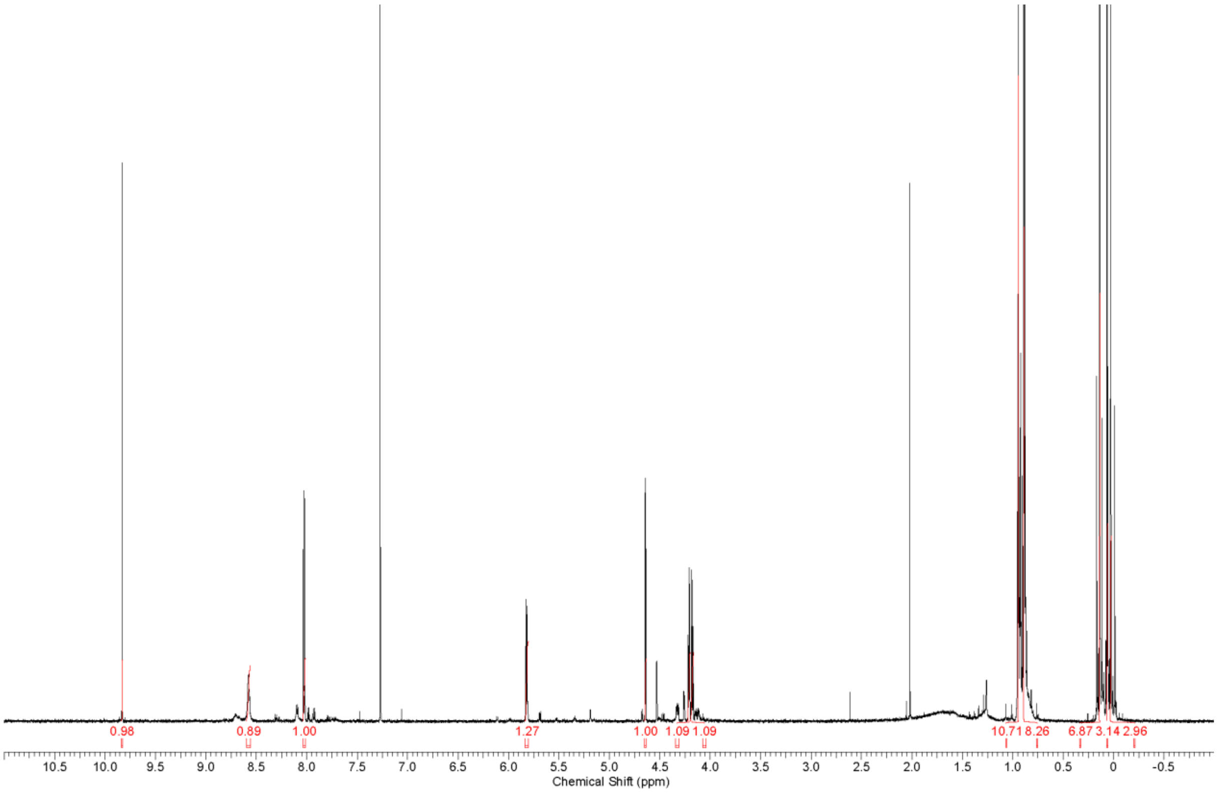

^{1}H NMR spectrum of **30** (500 MHz, $CDCl_3$).

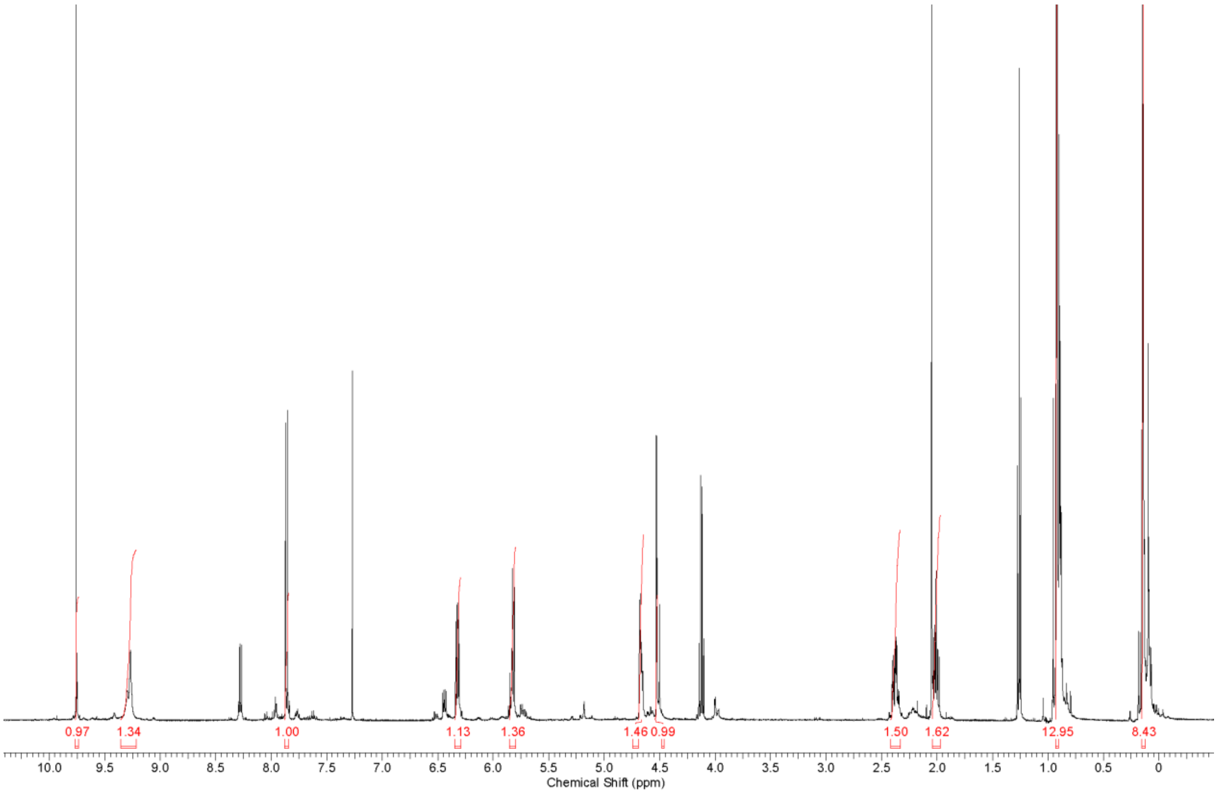

^{1}H NMR spectrum of **31** (500 MHz, $CDCl_3$).

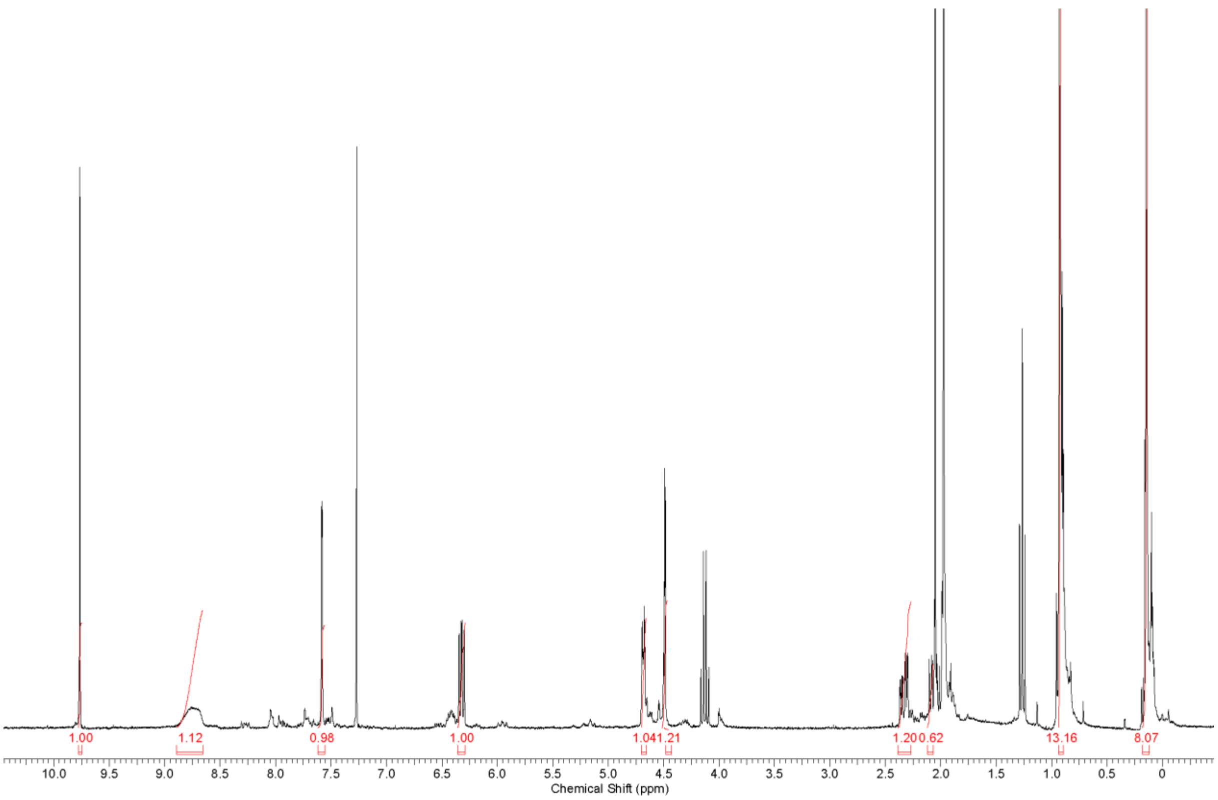

^{1}H NMR spectrum of **32** (500 MHz, $CDCl_3$).

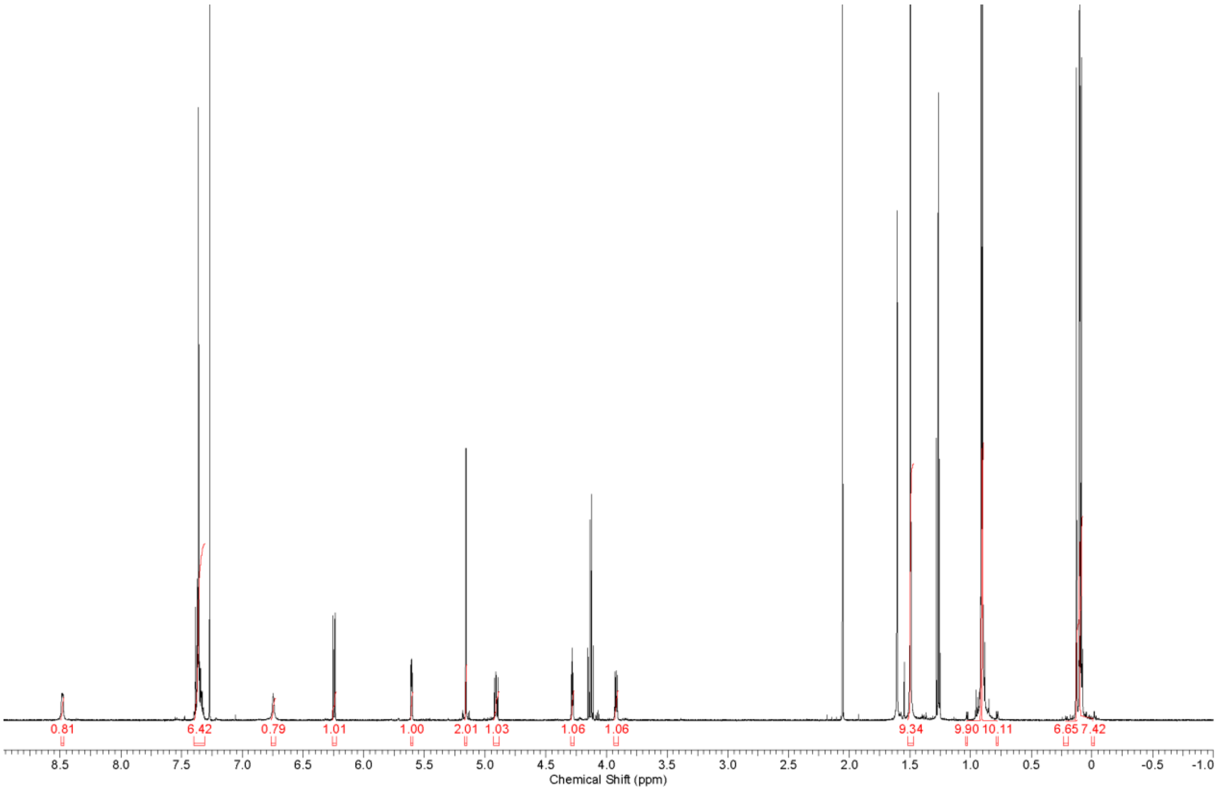

^{1}H NMR spectrum of **34** (500 MHz, $CDCl_3$).

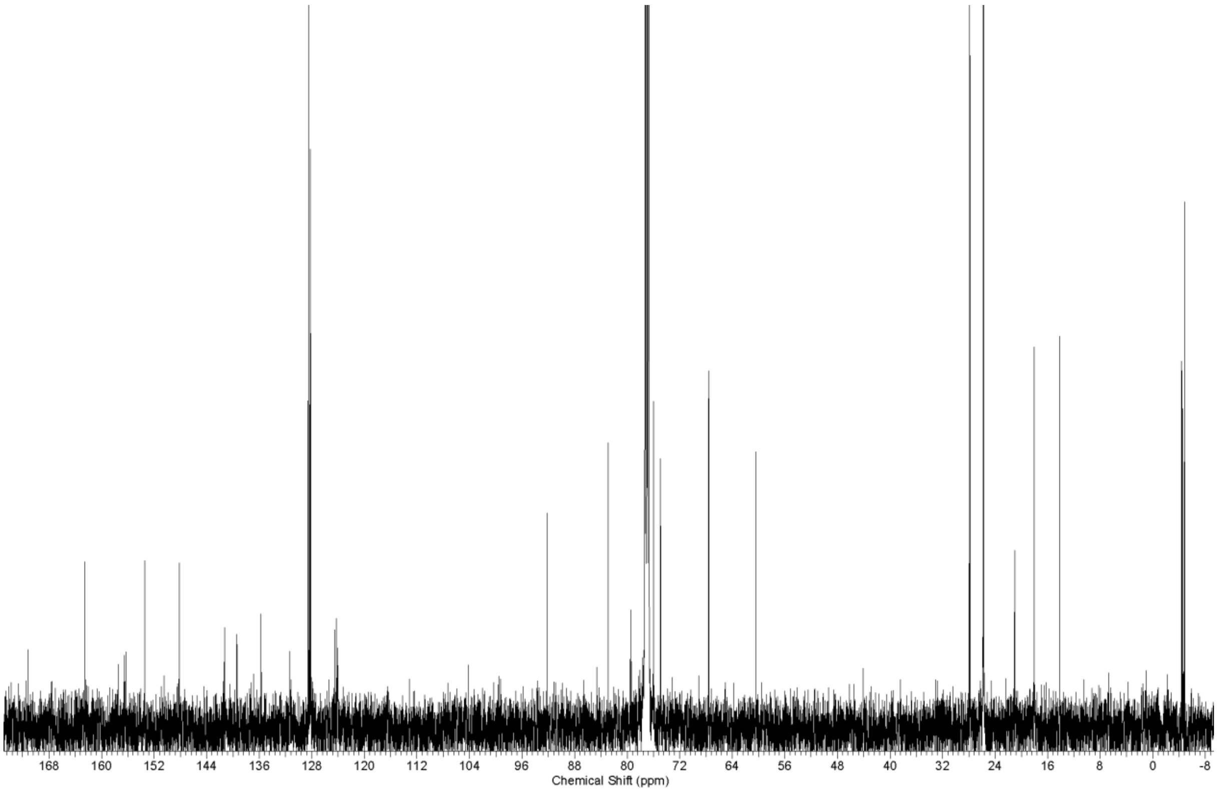

^{13}C NMR spectrum of **34** (126 MHz, $CDCl_3$).

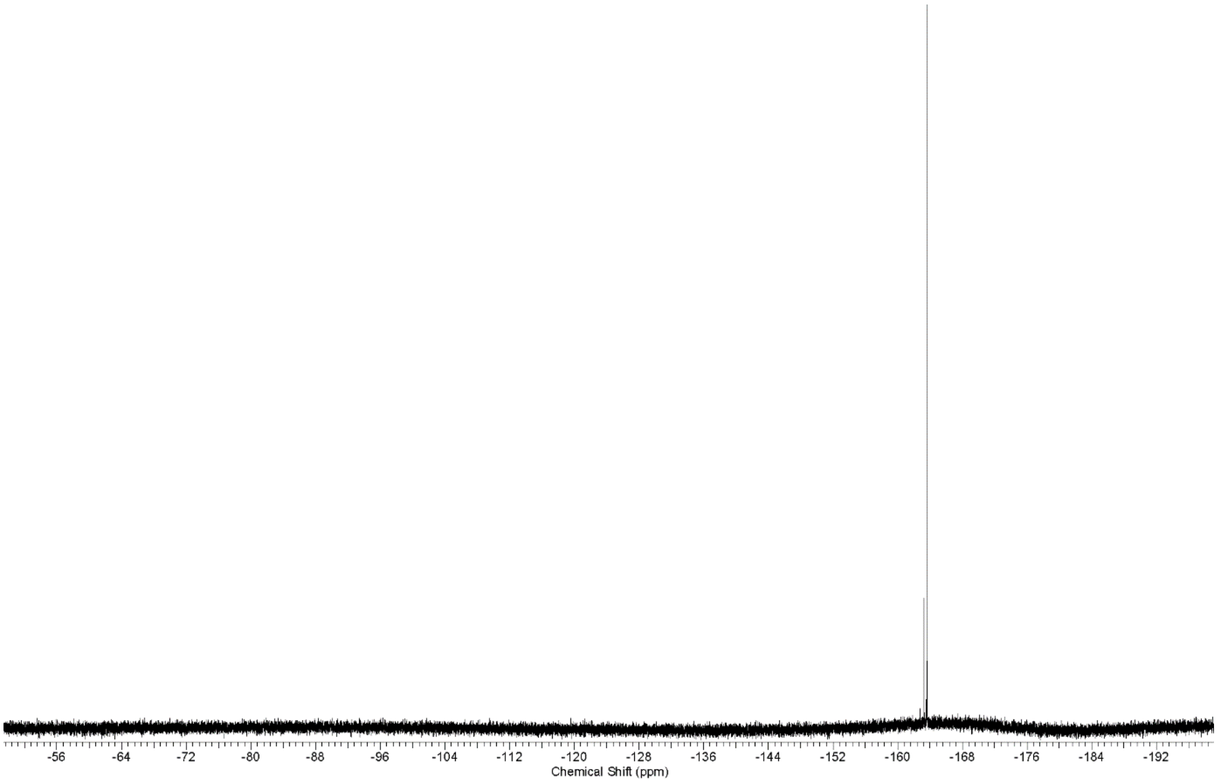

^{19}F NMR spectrum of **34** (376 MHz, $CDCl_3$).

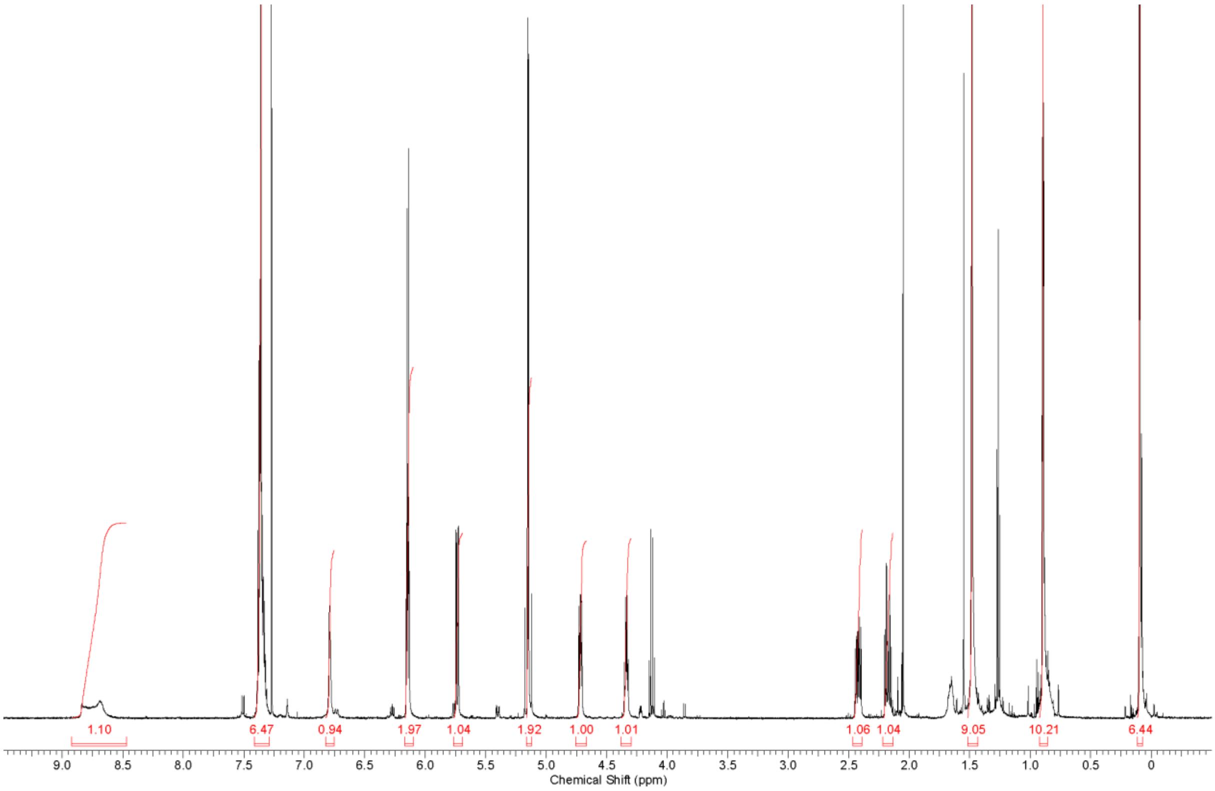

^{1}H NMR spectrum of **35** (500 MHz, $CDCl_3$).

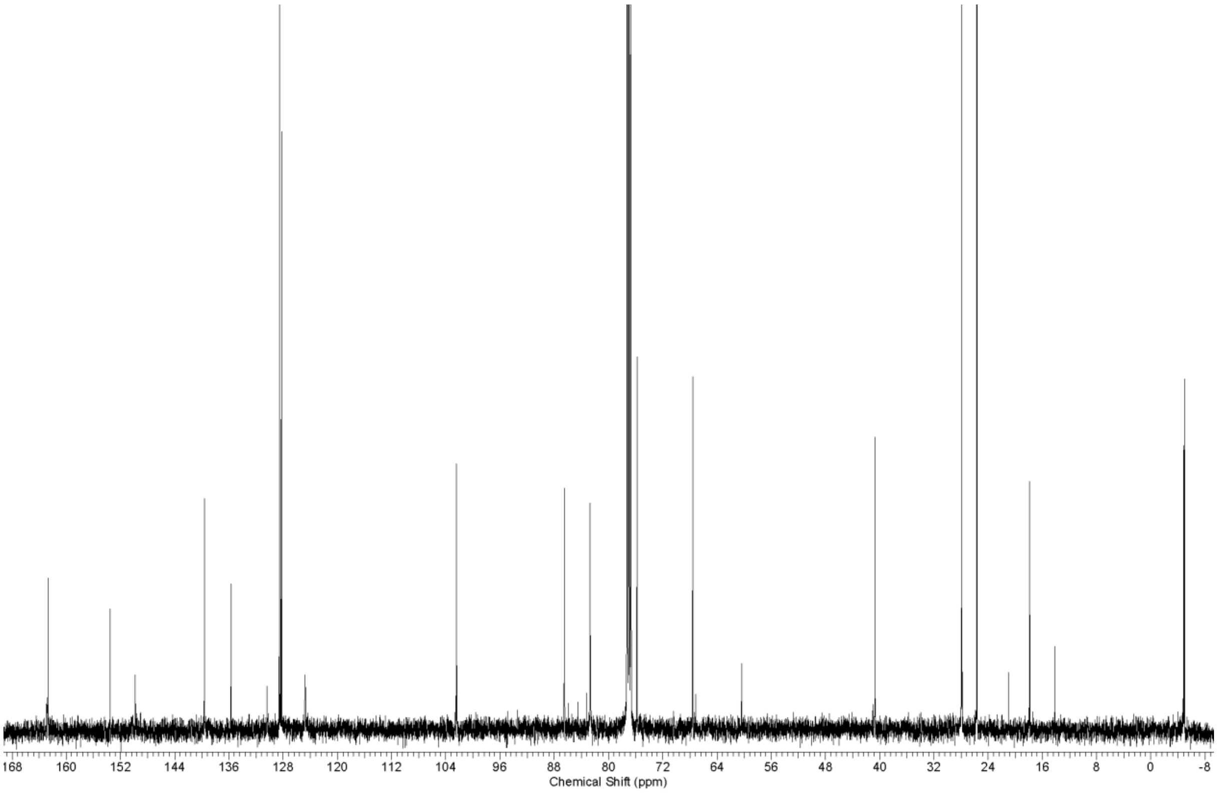

^{13}C NMR spectrum of **35** (126 MHz, $CDCl_3$).

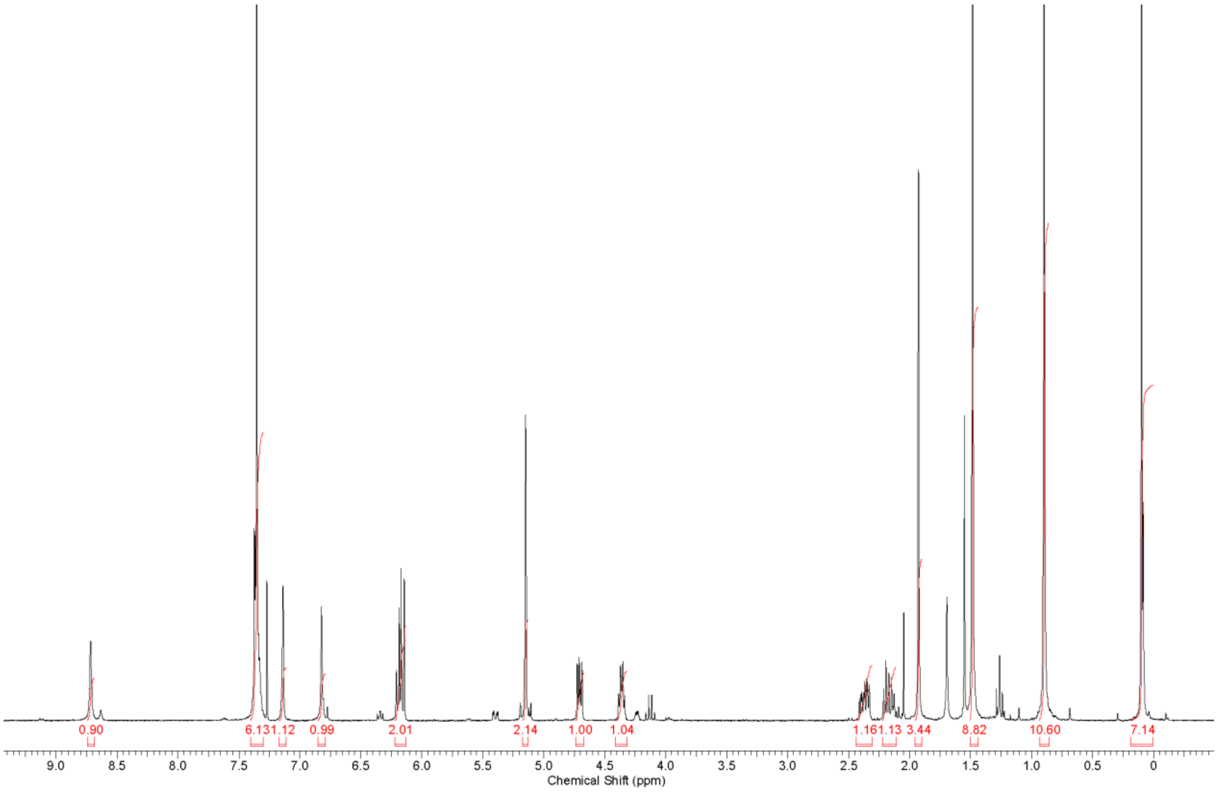

^{1}H NMR spectrum of **36** (300 MHz, $CDCl_3$).

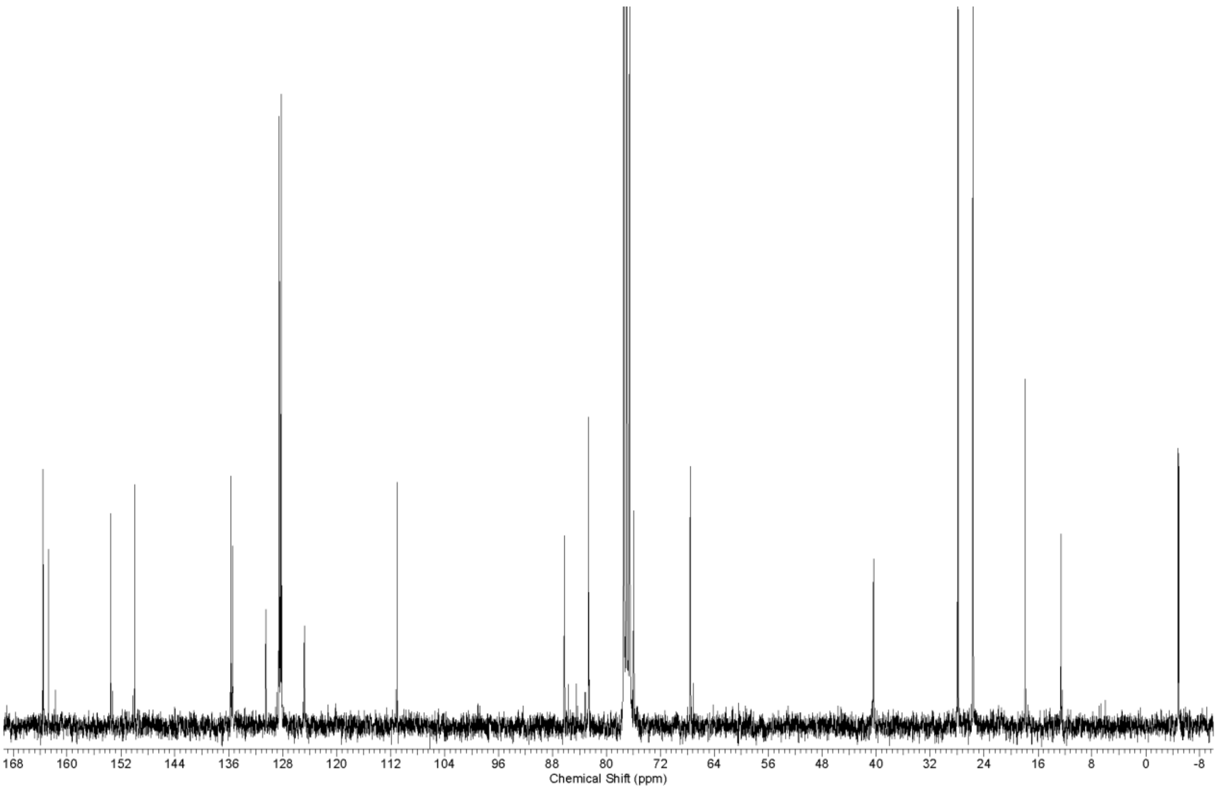

^{13}C NMR spectrum of **36** (126 MHz, $CDCl_3$).

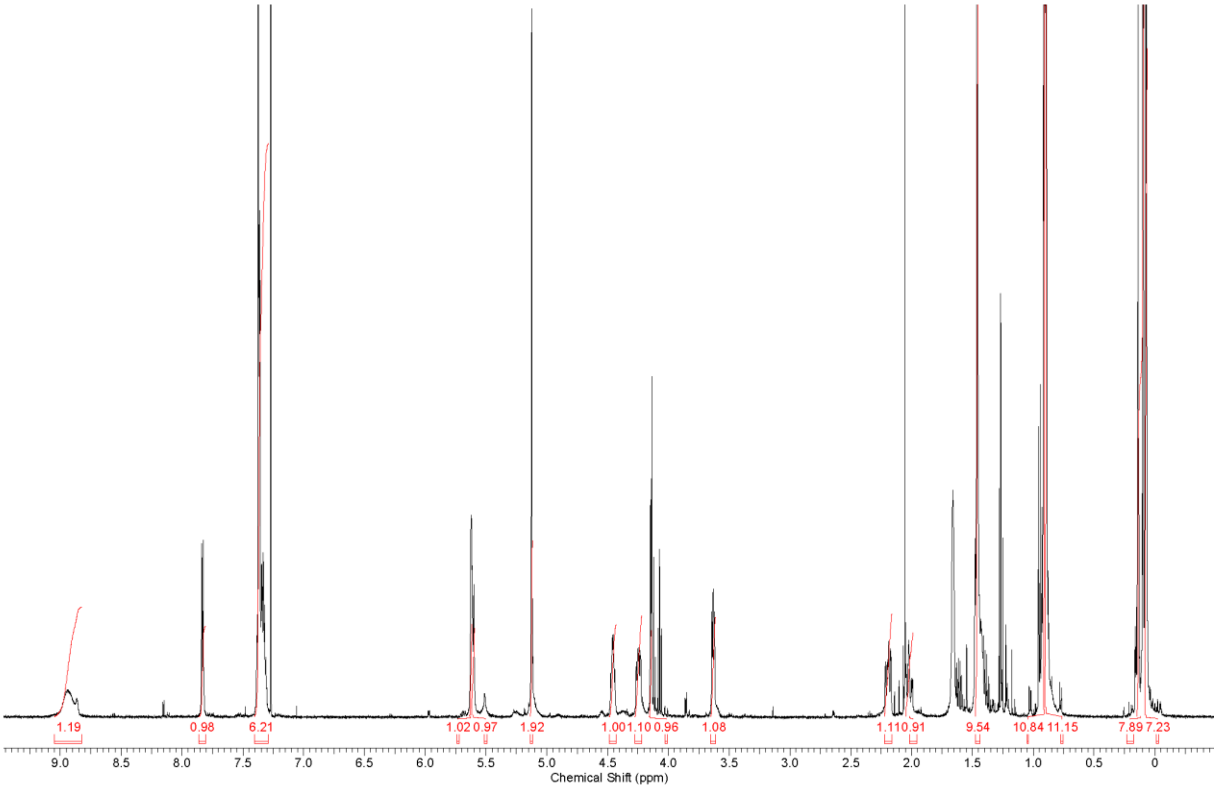

^{1}H NMR spectrum of **38** (500 MHz, $CDCl_3$).

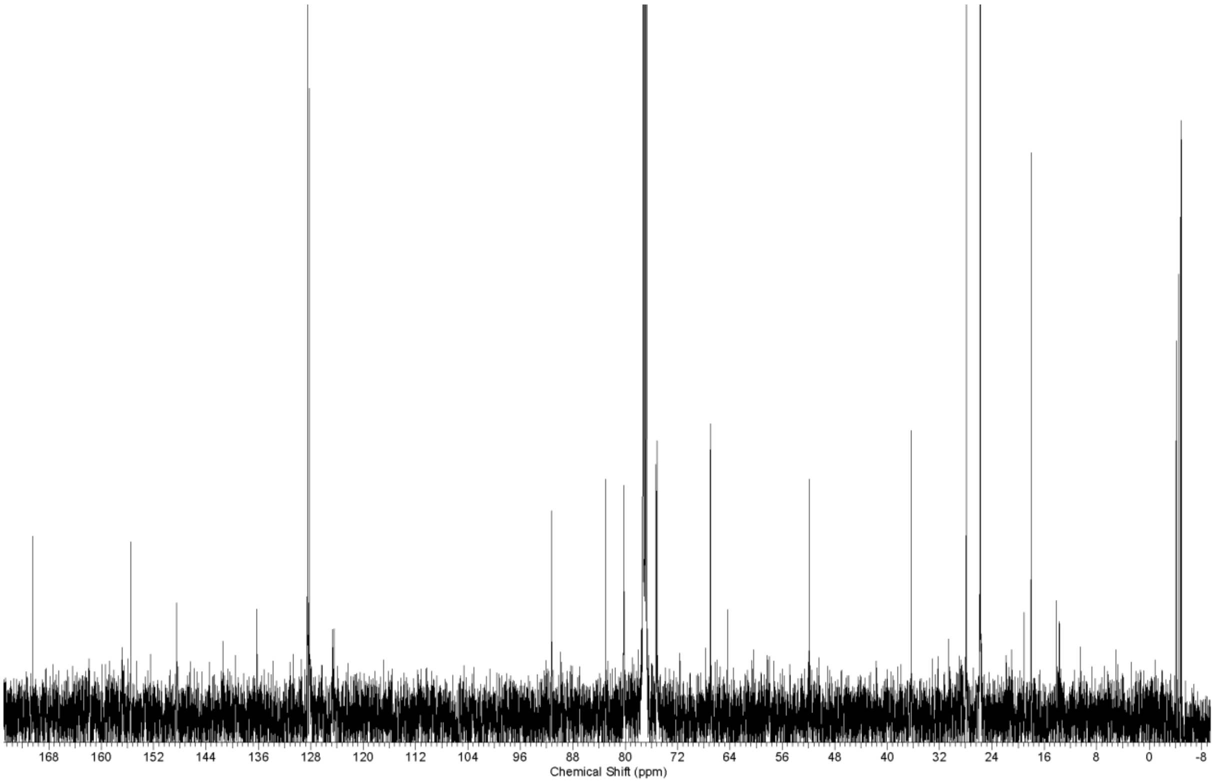

^{13}C NMR spectrum of **38** (126 MHz, $CDCl_3$).

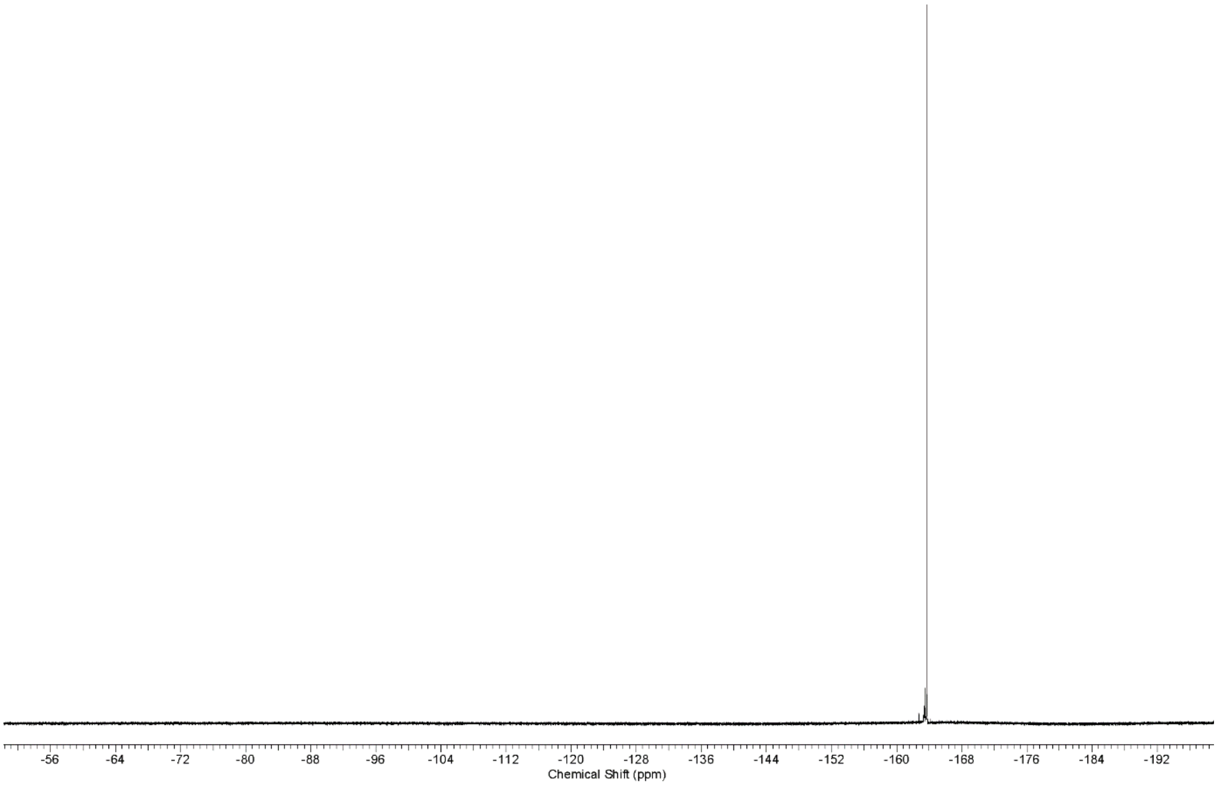

^{19}F NMR spectrum of **38** (376 MHz, $CDCl_3$).

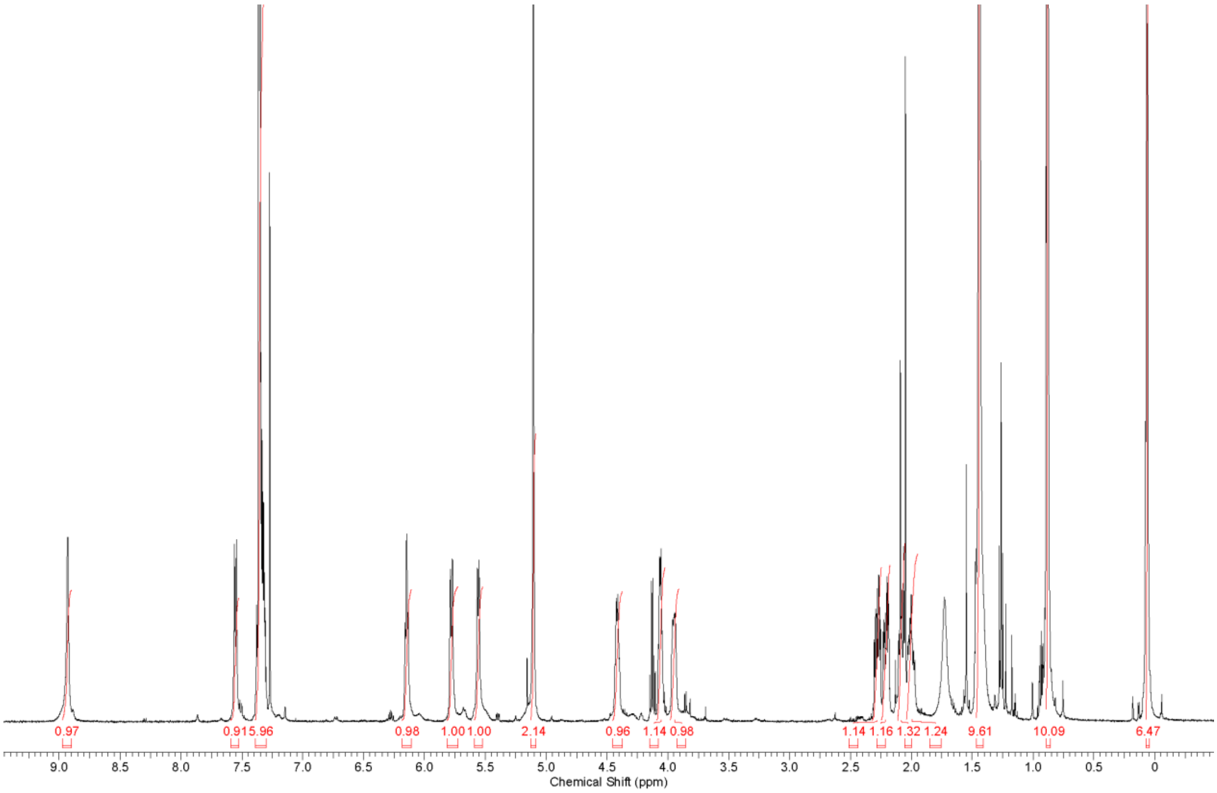

^{1}H NMR spectrum of **39** (500 MHz, $CDCl_3$).

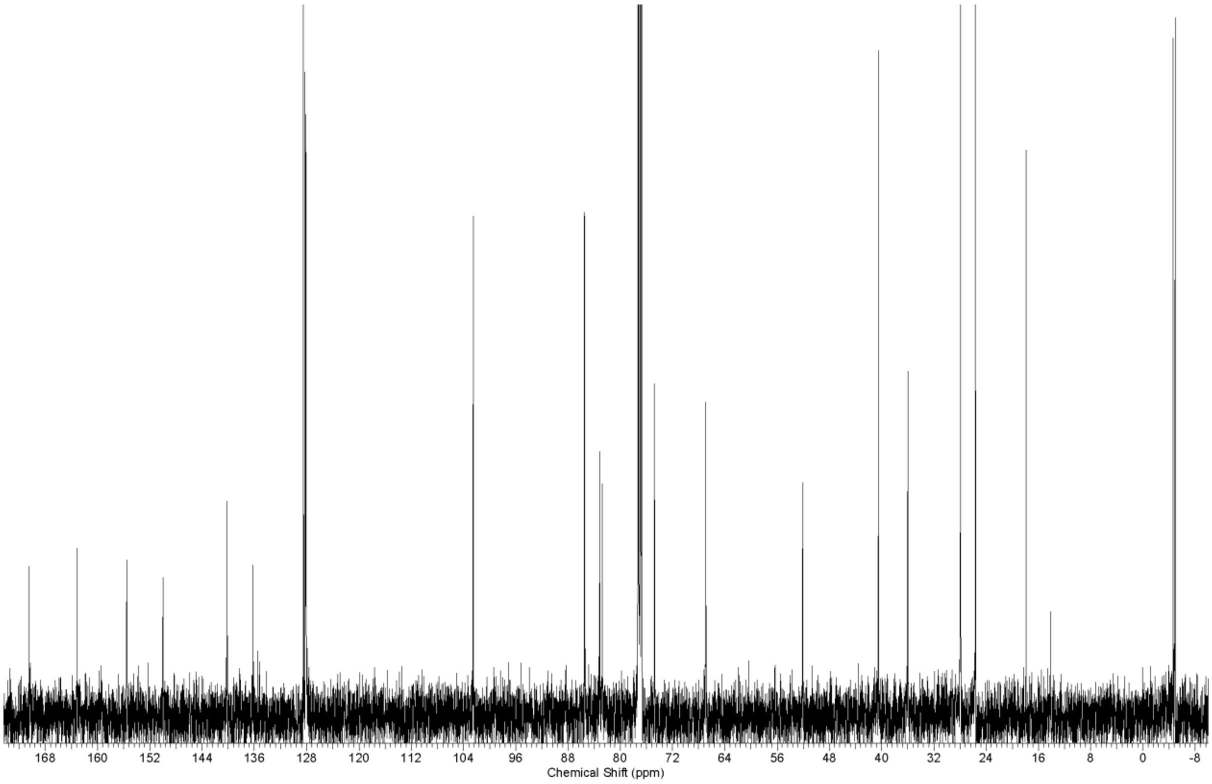

^{13}C NMR spectrum of **39** (126 MHz, $CDCl_3$).

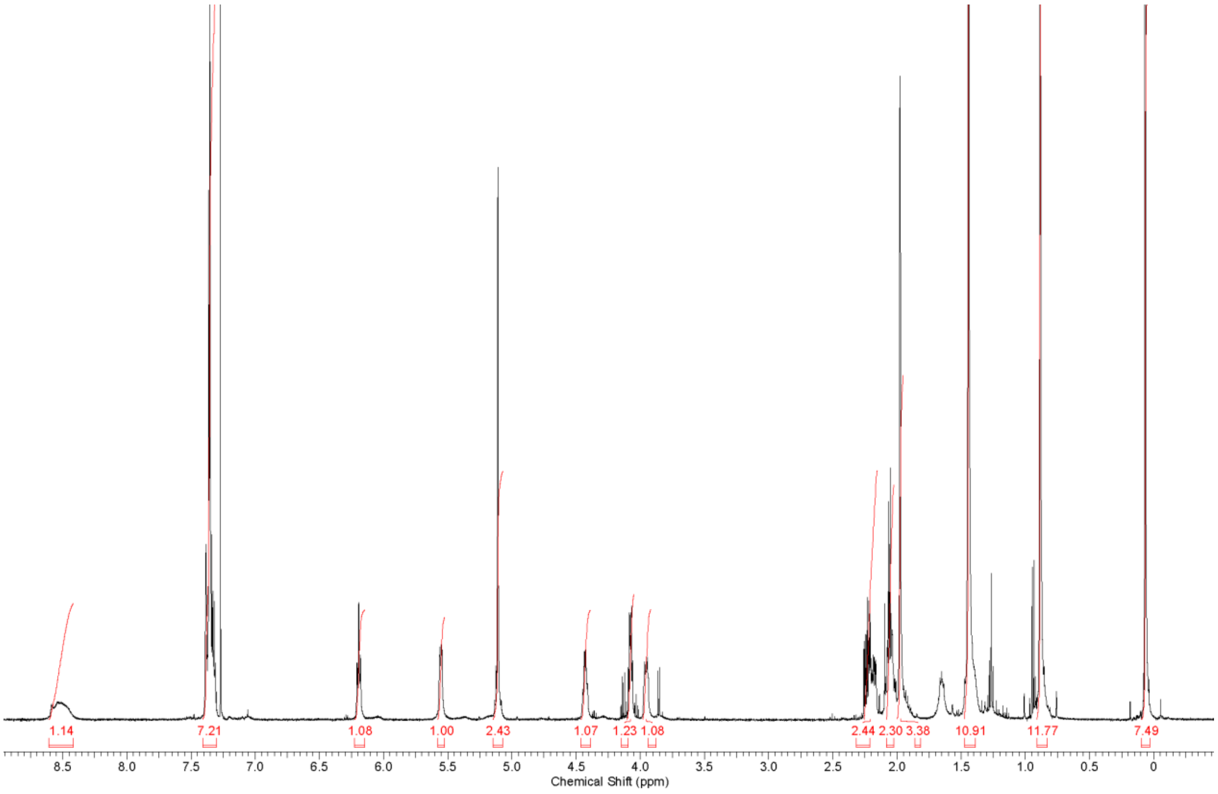

^{1}H NMR spectrum of **40** (500 MHz, $CDCl_3$).

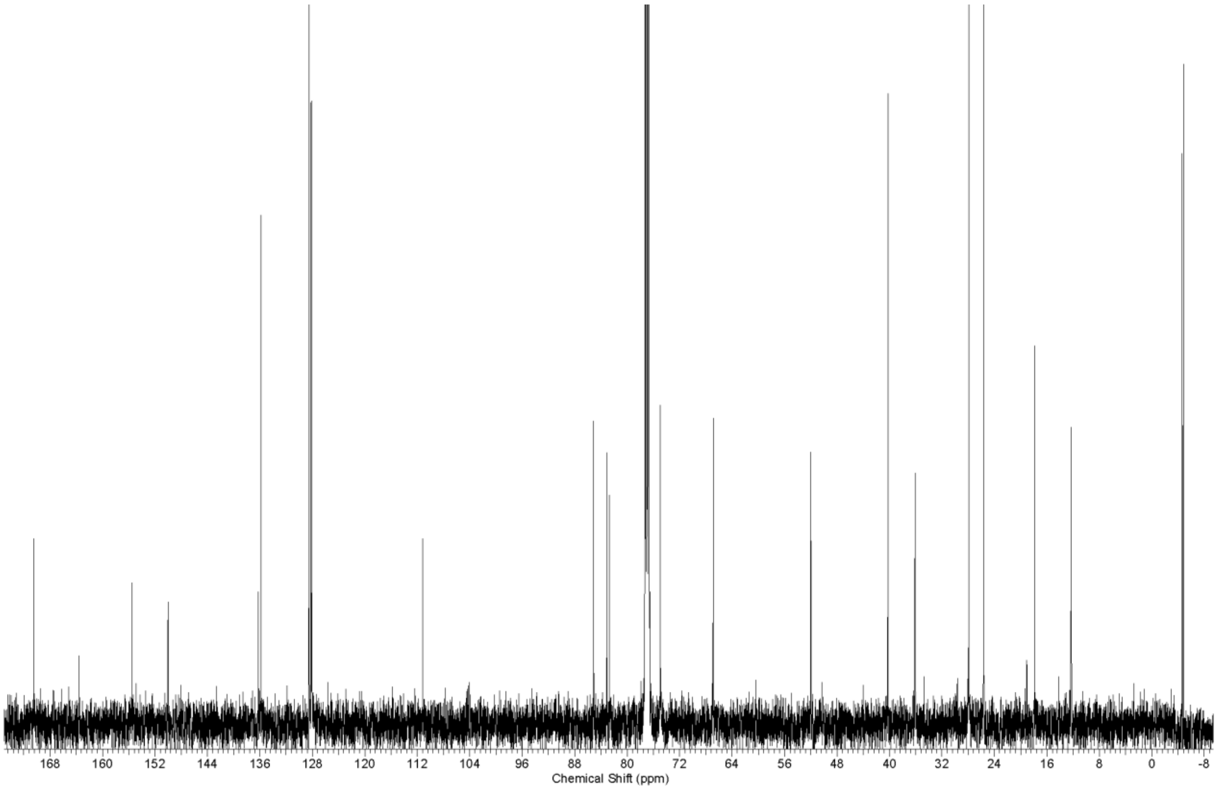

^{13}C NMR spectrum of **40** (126 MHz, $CDCl_3$).

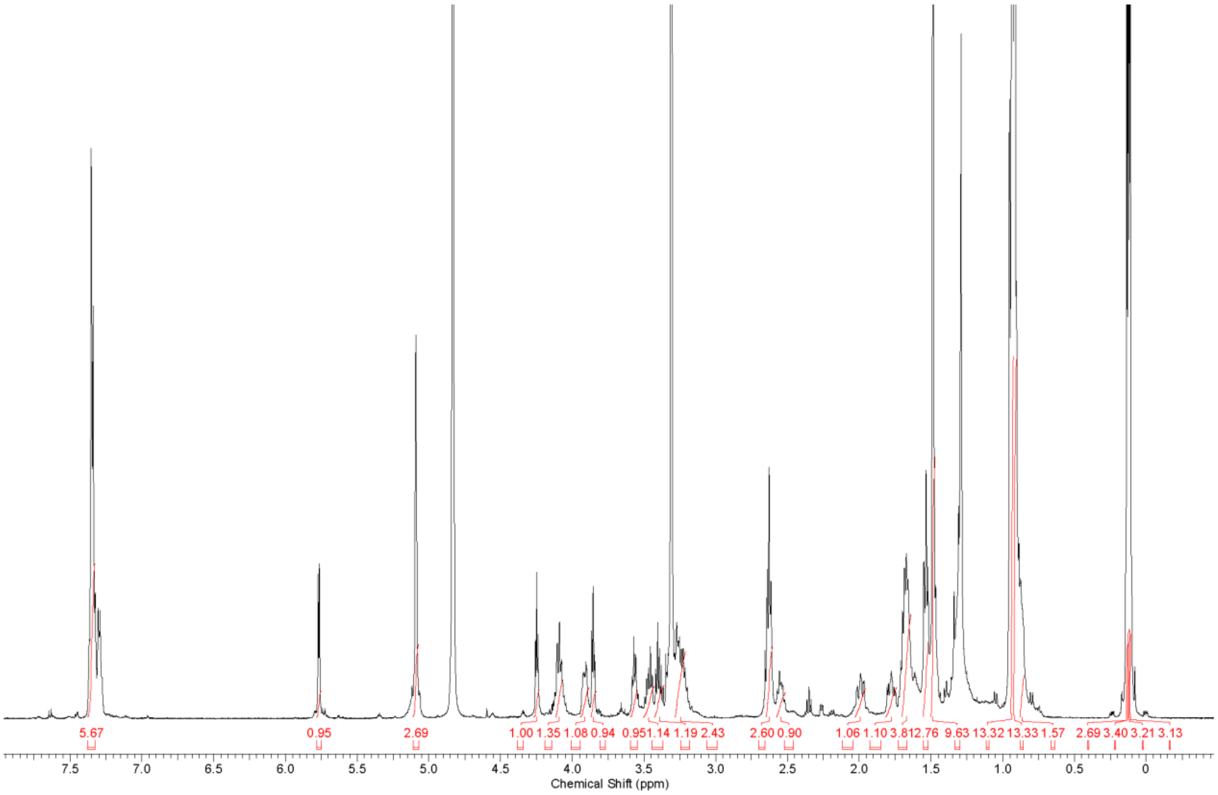

^{1}H NMR spectrum of **44** (500 MHz, CD_3OD).

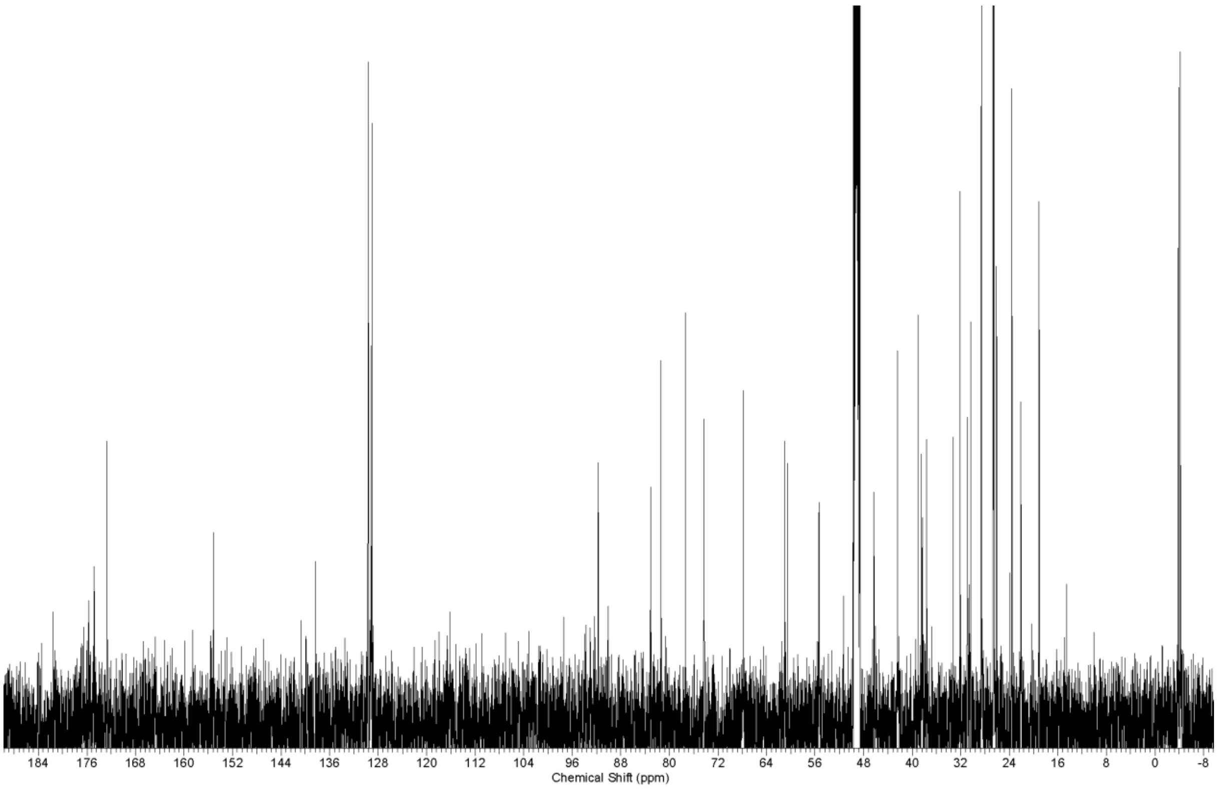

^{13}C NMR spectrum of **44** (126 MHz, CD_3OD).
